# Niraparib maintenance therapy using an individualised starting dose in patients with platinum-sensitive recurrent ovarian cancer (NORA): final overall survival analysis of a phase 3 randomised, placebo-controlled trial

**DOI:** 10.1016/j.eclinm.2024.102629

**Published:** 2024-05-07

**Authors:** Xiaohua Wu, Jianqing Zhu, Rutie Yin, Jiaxin Yang, Jihong Liu, Jing Wang, Lingying Wu, Ziling Liu, Yunong Gao, Danbo Wang, Ge Lou, Hongying Yang, Qi Zhou, Beihua Kong, Yi Huang, Lipai Chen, Guiling Li, Ruifang An, Ke Wang, Yu Zhang, Xiaojian Yan, Xin Lu, Weiguo Lu, Min Hao, Li Wang, Heng Cui, Qionghua Chen, Guzhalinuer Abulizi, Xianghua Huang, Xiaofei Tian, Hao Wen, Zhao Huang, Juan Dong, Charlie Zhang, Jianmei Hou, Mansoor R. Mirza

**Affiliations:** a Fudan University Shanghai Cancer Center, Shanghai, China; b Zhejiang Cancer Hospital, Hangzhou, China; c Department of Obstetrics and Gynecology, Key Laboratory of Birth Defects and Related Diseases of Women and Children, Ministry of Education, West China Second University Hospital, Sichuan University, Chengdu, China; d Peking Union Medical College Hospital, Chinese Academy of Medical Sciences and Peking Union Medical College, Beijing, China; e Sun Yat-sen University Cancer Center, Guangzhou, China; f Hunan Cancer Hospital, The Affiliated Cancer Hospital of Xiangya School of Medicine, Central South University, Changsha, China; g National Cancer Center/Cancer Hospital, Chinese Academy of Medical Sciences and Peking Union Medical College, Beijing, China; h The First Hospital of Jilin University, Changchun, China; i Peking University Cancer Hospital & Institute, Beijing, China; j Liaoning Cancer Hospital & Institute, Shenyang, China; k Harbin Medical University Cancer Hospital, Harbin, China; l The Third Affiliated Hospital of Kunming Medical University (Yunnan Cancer Hospital), Kunming, China; m Chongqing University Cancer Hospital, Chongqing, China; n Qilu Hospital of Shandong University, Jinan, China; o Hubei Cancer Hospital, Wuhan, China; p Affiliated Cancer Hospital of Guangzhou Medical University, Guangzhou, China; q Cancer Center, Union Hospital, Tongji Medical College, Huazhong University of Science and Technology, Wuhan, China; r The First Affiliated Hospital of Xi'an Jiaotong University, Xi'an, China; s Tianjin Medical University Cancer Institute and Hospital, Tianjin, China; t Xiangya Hospital, Central South University, Changsha, China; u The First Affiliated Hospital of Wenzhou Medical University, Wenzhou, China; v Obstetrics & Gynecology Hospital of Fudan University, Shanghai, China; w Women's Hospital, School of Medicine, Zhejiang University, Hangzhou, China; x The Second Hospital of Shanxi Medical University, Taiyuan, China; y Affiliated Cancer Hospital of Zhengzhou University, Henan Cancer Hospital, Zhengzhou, China; z Peking University People's Hospital, Beijing, China; aa The First Affiliated Hospital of Xiamen University, Xiamen, China; ab The Affiliated Cancer Hospital of Xinjiang Medical University, Urumqi, China; ac The Second Hospital of Hebei Medical University, Shijiazhuang, China; ad Shaanxi Provincial Cancer Hospital, Xi'an, China; ae Zai Lab (Shanghai) Co., Ltd, Shanghai, China; af Rigshospitalet, Copenhagen University Hospital, Copenhagen, Denmark

**Keywords:** PARP inhibitor, Recurrent ovarian cancer, Maintenance therapy, Overall survival

## Abstract

**Background:**

Niraparib significantly prolonged progression-free survival versus placebo in patients with platinum-sensitive, recurrent ovarian cancer (PSROC), regardless of germline *BRCA* mutation (g*BRCA*m) status, in NORA. This analysis reports final data on overall survival (OS).

**Methods:**

This randomised, double-blind, placebo-controlled, phase 3 trial enrolled patients across 30 centres in China between 26 September 2017 and 2 February 2019 (clinicaltrials.gov, NCT03705156). Eligible patients had histologically confirmed, recurrent, (predominantly) high-grade serous epithelial ovarian cancer, fallopian tube carcinoma, or primary peritoneal carcinoma (no histological restrictions for those with g*BRCA*m) and had received ≥2 prior lines of platinum-based chemotherapy. Patients were randomised (2:1) to receive niraparib or placebo, with stratification by g*BRCA*m status, time to recurrence following penultimate platinum-based chemotherapy, and response to last platinum-based chemotherapy. Following a protocol amendment, the starting dose was individualised: 200 mg/day for patients with bodyweight <77 kg and/or platelet count <150 × 10^3^/μL at baseline and 300 mg/day otherwise. OS was a secondary endpoint.

**Findings:**

Totally, 265 patients were randomised to receive niraparib (n = 177) or placebo (n = 88), and 249 (94.0%) received an individualised starting dose. As of 14 August 2023, median follow-up for OS was 57.9 months (IQR, 54.8–61.6). Median OS (95% CI) with niraparib versus placebo was 51.5 (41.4–58.9) versus 47.6 (33.3–not evaluable [NE]) months, with hazard ratio [HR] of 0.86 (95% CI, 0.60–1.23), in the overall population; 56.0 (36.1–NE) versus 47.6 (31.6–NE) months, with HR of 0.86 (95% CI, 0.46–1.58), in patients with g*BRCA*m; and 46.5 (41.0–NE) versus 46.9 (31.8–NE) months, with HR of 0.87 (95% CI, 0.56–1.35), in those without. No new safety signals were identified, and myelodysplastic syndromes/acute myeloid leukaemia occurred in three (1.7%) niraparib-treated patients.

**Interpretation:**

Niraparib maintenance therapy with an individualised starting dose demonstrated a favourable OS trend versus placebo in PSROC patients, regardless of g*BRCA*m status.

**Funding:**

Zai Lab (Shanghai) Co., Ltd; National Major Scientific and Technological Special Project for “Significant New Drugs Development” in 2018, China [grant number 2018ZX09736019].

**Research in context**

**Evidence before this study**

We searched PubMed for clinical trials of poly (ADP-ribose) polymerase (PARP) inhibitors in patients with platinum-sensitive recurrent ovarian cancer (PSROC) using the search terms ((poly (ADP-ribose) polymerase inhibitor) OR (PARP inhibitor) OR (niraparib) OR (olaparib) OR (rucaparib) OR (fuzuloparib)) AND (ovarian cancer) AND (maintenance) AND ((platinum-sensitive relapsed) OR (platinum-sensitive recurrent)), without date or language restrictions, and supplemented this search with recent narrative review articles and conference abstracts. In prior trials in PSROC patients, PARP inhibitors (olaparib in Study 19 and SOLO2, niraparib in NOVA, and rucaparib in ARIEL-3) as maintenance therapy have consistently demonstrated a favourable trend in overall survival (OS) versus placebo among those with *BRCA* mutations, but conflicting OS trends have been observed across these trials for patients without *BRCA* mutations.

**Added value of this study**

To our knowledge, NORA is the first phase 3 trial that demonstrated a favourable OS trend with a PARP inhibitor (niraparib) versus placebo in PSROC patients both with and without germline *BRCA* mutations, although the study was not designed to power OS analysis and a considerable proportion of patients in the placebo group received subsequent treatment with PARP inhibitors. Notably, NORA is also the first phase 3 trial of niraparib where a predominant proportion (94%) of patients used the individualised starting dose strategy, which improved the tolerability profile of niraparib compared with NOVA, in which a fixed starting dose was used for all patients. This improved tolerability profile might lead to better compliance, longer treatment duration, increased exposure, and thereby better efficacy, which may partly account for the different OS trends observed across NORA and NOVA for patients without *BRCA* mutations.

**Implications of all the available evidence**

The favourable OS trend with niraparib versus placebo in patients both with and without germline BRCA mutations from NORA continues to support the use of niraparib maintenance therapy with an individualised starting dose in the all-comer population in the recurrence setting for ovarian cancer.

## Introduction

Ovarian cancer is the second leading cause, just behind cervical cancer, for mortality due to gynaecological malignancies worldwide,^1^ largely because most patients present with advanced disease at diagnosis.^2^ Although most patients with advanced ovarian cancer respond to front-line platinum-based chemotherapy, approximately 70% of them experience recurrence within three years and typically require multiple subsequent lines of platinum-based chemotherapy.^3^ However, without any intervention in between, time to recurrence progressively shortens following each ensuing platinum regimen, with concomitant cumulative increase in toxicity.^4^

The advent of poly (adenosine diphosphate-ribose) polymerase (PARP) inhibitors such as niraparib, olaparib, and rucaparib has revolutionized the treatment landscape of ovarian cancer. They as maintenance therapy confer statistically significant and clinically meaningful improvements in progression-free survival (PFS) versus placebo after chemotherapy in patients with platinum-sensitive disease.^5^^,^^6^ In patients with platinum-sensitive, recurrent ovarian cancer (PSROC), a favourable trend in overall survival (OS) with PARP inhibitor maintenance therapy versus placebo has been consistently demonstrated among those with *BRCA* mutations across trials.^7^, ^8^, ^9^, ^10^ However, it is still a subject of debate whether PSROC patients without *BRCA* mutations can also receive OS benefits from PARP inhibitor maintenance therapy because conflicting OS trends across clinical trials of PARP inhibitors have been observed for this patient population.^7^^,^^9^^,^^10^ Considering these findings, the indications of PARP inhibitors as maintenance therapy for PSROC have been restricted to those with *BRCA* mutations only in the United States.^11^, ^12^, ^13^

Niraparib is a potent, highly selective PARP inhibitor that has demonstrated significant PFS benefits versus placebo in pivotal phase 3 trials (PRIMA, PRIME, NOVA, and NORA) in the maintenance setting for both newly diagnosed and recurrent ovarian cancer that is platinum-sensitive, regardless of biomarker status.^14^, ^15^, ^16^, ^17^ Notably, bodyweight and platelet count at baseline were identified as risk factors for the increased occurrence of grade ≥3 thrombocytopenia in niraparib-treated patients by a retrospective analysis of the NOVA study, a phase 3 trial in PSROC patients.^18^ In light of this finding, subsequent phase 3 trials of niraparib in ovarian cancer (NORA, PRIMA, and PRIME) incorporated in a body weight and platelet count-guided individualised starting dose strategy through protocol amendment or prospectively.^15^, ^16^, ^17^

NORA is a phase 3 randomised, double-blind, placebo-controlled trial to evaluate PFS benefit of niraparib versus placebo in the maintenance setting for PSROC in China.^16^ It is the first phase 3 trial in ovarian cancer that used the individualised starting dose strategy in a predominant proportion (94.0%) of enrolled patients, demonstrating improved safety and tolerability profiles of niraparib compared with the NOVA trial, in which all patients received a fixed starting dose.^14^^,^^16^ In the primary analysis, niraparib significantly prolonged PFS versus placebo (median, 18.3 versus 5.4 months; hazard ratio [HR], 0.32; 95% confidence interval [CI], 0.23–0.45; p < 0.0001), irrespective of germline *BRCA* mutation status.^16^ In an interim analysis conducted after 44.2% (117/265) of patients had OS events, niraparib demonstrated a favourable OS trend versus placebo, regardless of germline *BRCA* mutation status, providing more evidence for the beneficial effect of niraparib in improving long-term prognosis.^19^

This final analysis of the NORA study aims to update results for OS and other secondary efficacy endpoints and report long-term incidence of myelodysplastic syndrome (MDS) and acute myeloid leukaemia (AML).

## Methods

### Study design and patients

The NORA study was a randomised, double-blind, placebo-controlled, phase 3 trial, conducted across 30 study centres in China. Detailed descriptions of the methodology have been published previously.^16^ Briefly, eligible patients were women aged 18 years or older, had an Eastern Cooperative Oncology Group performance status of 0 or 1, and had histologically confirmed, recurrent, (predominantly) high-grade serous epithelial ovarian cancer, fallopian tube carcinoma, or primary peritoneal carcinoma (collectively defined as ovarian cancer), but there were no histological restrictions for those with germline *BRCA* mutations. Patients provided blood samples for germline *BRCA* mutation testing using the g*BRCA* testing assay (BGI Genomics, Shenzhen, China) at a central laboratory. All patients had received at least two prior lines of platinum-based chemotherapy. For the penultimate platinum-based chemotherapy, patients had achieved a complete or partial response and had disease progression at least six months after completion of this line of chemotherapy. For the most recent platinum-based chemotherapy, patients had received at least four cycles of treatment (carboplatin, cisplatin, or nedaplatin) and achieved a complete or partial response. Patients were excluded if they had been previously treated with any PARP inhibitor. The full exclusion and inclusion criteria are provided in the Appendix.

### Ethics statement

All patients provided written informed consent before their enrolment. The study was approved by the ethics committees of the leading centre (Fudan University Shanghai Cancer Center; approval number, 1707174-8-1901C) and all other participating centres, and conducted in compliance with the Declaration of Helsinki, Good Clinical Practice, and all applicable laws and regulations.

### Randomisation and masking

Within eight weeks after completion of their most recent platinum-based chemotherapy, patients were randomised (2:1) to receive niraparib or matched placebo, with stratification according to germline *BRCA* mutation status (positive or negative), time to recurrence following the penultimate platinum-based chemotherapy (6–<12 months or ≥12 months), and response to the most recent platinum-based chemotherapy (complete or partial). Permuted block randomisation with a block size of six was performed centrally using an interactive web response system, which provided each eligible patient with a computer-generated random number via an algorithm developed by statisticians who were not involved in the trial otherwise. These random numbers linked patients to their treatment allocation, which was blinded to all patients, investigators, study coordinators, and the study sponsor until the primary analysis of PFS. Before that, unblinding was allowed only for medical emergencies that required the knowledge of assigned treatment. Masking was achieved by using niraparib and placebo capsules (manufactured by Zai Lab [Suzhou] Co., Ltd., Suzhou, China) that were identical in appearance and packaging.

### Procedures

Patients received their assigned treatment orally once daily in 28-day treatment cycles until disease progression according to the Response Evaluation Criteria in Solid Tumors (RECIST) version 1.1, intolerable toxicity, patient withdrawal, or other protocol-specified reasons (provided in the Appendix), whichever occurred first. At the beginning of the trial, the starting dose was fixed at 300 mg (three 100-mg capsules) per day. After the protocol amendment on 12 December 2017 in light of retrospective analysis results of the NOVA study,^18^ an individualised starting dose was used: 200 mg (two 100-mg capsules) per day for those with a body weight less than 77 kg and/or a platelet count lower than 150,000 per μL at baseline and otherwise, 300 mg per day. Dose interruptions and/or reductions were permitted for the management of adverse events (Supplementary Table S1). The daily dose could be increased to 300 mg for those who started treatment with 200 mg per day but experienced no dose interruption or reduction in the first two treatment cycles. Treatment crossover was not permitted until the study was unblinded after the data cut-off date for the primary analysis. Upon study unblinding, patients in the niraparib group who were still receiving study treatment could continue niraparib treatment until they met the treatment discontinuation criteria specified in the protocol and patients from the placebo group who were receiving study treatment were provided the option to switch to niraparib treatment at the investigators’ discretion.

Tumour assessments according to RECIST version 1.1 were performed using contrast-enhanced computerised tomography or magnetic resonance imaging scans at baseline, every eight weeks until week 56, and every 12 weeks thereafter, until disease progression. Radiographic assessments were continued as scheduled until disease progression for those who discontinued study treatment for reasons other than disease progression. Safety was assessed through laboratory investigations, monitoring of vital signs, and physical examinations. Patients who discontinued treatment but remained in the study were continued to be followed every 90 days for survival, subsequent anti-cancer therapy, and new malignancies.

### Outcomes

The primary endpoint, which was PFS (time from randomisation to disease progression on study drug or death of any cause, whichever occurred earlier) assessed by blinded independent central review per RECIST version 1.1, has been previously reported.^16^ The secondary endpoints included OS (time from randomisation to death of any cause), chemotherapy-free interval (CFI, time from completion of the most recent platinum-based chemotherapy to subsequent anti-cancer therapy [excluding maintenance therapy]), time to first subsequent anti-cancer therapy (TFST, time from randomisation to first subsequent anti-cancer treatment or death, whichever came earlier), and safety. This final analysis reported OS as well as updated results for CFI and TFST. The incidence of MDS/AML was also analysed and reported.

### Statistical analysis

Assuming a true median PFS of 9.3 months for patients receiving niraparib and 5.0 months for patients receiving placebo and a true HR of 0.54 for disease progression or death with niraparib versus placebo, a sample size of 240 patients (niraparib, 160; placebo, 80) was determined as appropriate to provide an at least 95% power to detect a significant difference in PFS at a two-sided significance level of 5%.^16^ The primary analysis was planned to take place after the occurrence of 155 PFS events or 12 months after the randomisation of the last patient, whichever occurred earlier. The planned final analysis was scheduled to take place when OS events occurred in at least 50% of the intention-to-treat population or 48 months after the primary analysis, whichever occurred earlier.

The Kaplan–Meier method was used to generate survival curves for time-to-event endpoints (OS, CFI and TFST) and estimate median time to event. The stratified log-rank test was used to assess the statistical significance of differences in endpoints between treatment groups. HRs and their corresponding 95% confidence intervals (CIs) were estimated using Cox proportional hazards models, adjusting for the stratification factors. The validity of the proportional hazard assumption was assessed by examining the Schoenfeld residual plots and then introducing a time-dependent covariate into the Cox models. Since the study was not powered for the analysis of secondary efficacy endpoints, this final analysis was descriptive in nature and obtained p values were nominal and are not reported. A subgroup analysis by germline *BRCA* mutation status was conducted for OS, CFI, and TFST.

Progression-free survival 2 (PFS-2) was studied in a *post-hoc* exploratory analysis and analysed in the same approach for secondary efficacy endpoints. It was defined as time from randomisation to disease progression (as determined by healthcare providers based on clinical and/or radiographic assessment) on the first subsequent anti-cancer therapy or death of any cause, whichever occurred earlier. A subgroup analysis by germline *BRCA* mutation status was also conducted for PFS-2.

The incidences of MDS/AML and second primary malignancies other than MDS/AML were summarised descriptively.

Efficacy endpoints were evaluated in the intention-to-treat population comprising all randomised patients. OS was also evaluated for a *post-hoc* analysis in the per-protocol population comprising all randomised patients with no major protocol deviations that might significantly affect efficacy evaluations. The efficacy results reported pertain to the intention-to-treat population, unless specified otherwise. Safety analysis was conducted in the safety analysis set comprising all randomised patients who received at least one dose of the study treatment. All statistical analyses were conducted using the SAS software version 9.4 (SAS institute, Cary, NC, USA).

The study was overseen by an independent data monitoring committee. This study was registered with ClinicalTrials.gov (identifier, NCT03705156).

### Role of the funding source

The trial was designed by the sponsor, Zai Lab, which was also responsible for overseeing the collection, analysis, and interpretation of the data. This report was written by the authors, with medical writing support funded by the sponsor. The decision to submit the manuscript for publication was made by all authors. All authors had full access to the data and the corresponding author had the final responsibility to submit for publication.

## Results

**Fig. 1:**
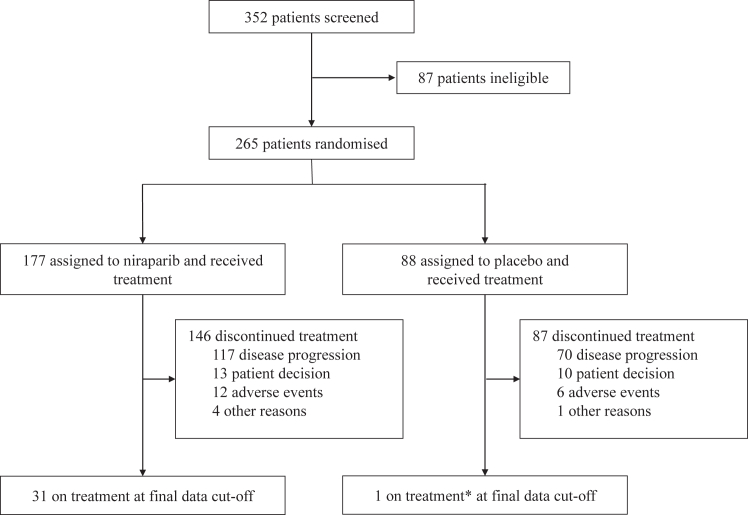
**Trial CONSORT diagram.** ∗The patient switched to niraparib after unblinding at the data cut-off for the primary analysis and was receiving treatment with niraparib at the final data cut-off.

From 26 September 2017 to 2 February 2019, 352 patients were screened and 265 of them were randomised to receive niraparib (n = 177) or placebo (n = 88) (Fig. 1). All patients had received at least one dose of the study treatment, and 249 (94.0%) patients received an individualised starting daily dose. After the data cut-off date for the primary analysis (1 February 2020), the study was unblinded. Demographics and baseline characteristics were well balanced between the two groups (Table 1).

**Table 1:** Demographics and baseline characteristics.

Characteristic	Niraparib (n = 177)	Placebo (n = 88)
Median age (range), years	53.0 (35–78)	55.0 (38–72)
Median weight (range), kg	61.0 (39–93)	60.5 (40–88)
Mean body mass index (SD), kg/m^2^	24.4 (3.68)	24.2 (3.36)
ECOG performance status, n (%)		
0	70 (40)	35 (40)
1	107 (61)	53 (60)
FIGO stage at diagnosis, n (%)		
I or II	26 (15)	17 (19)
III	128 (72)	64 (73)
IV	21 (12)	7 (8)
Missing	2 (1)	0
Histologic subtype, n (%)		
High-grade serous ovarian cancer	174 (98)	86 (98)
Endometrioid carcinoma	1 (1)	0
Mucinous carcinoma	0	0
Other	2 (1)	2 (2)
Secondary cytoreduction surgery received, n (%)	48 (27)	21 (24)
Time to recurrence after penultimate platinum-based chemotherapy, n (%)		
6 to <12 months	56 (32)	28 (32)
≥12 months	121 (68)	60 (68)
Best response to most recent platinum-based chemotherapy, n (%)		
Complete	91 (51)	46 (52)
Partial	86 (49)	42 (48)
Number of prior lines of chemotherapy, n (%)		
2	177 (100)	88 (100)
Previous bevacizumab use, n (%)	11 (6)	7 (8)
Germline *BRCA* mutation status, n (%)		
Germline *BRCA* mutation	65 (37)	35 (40)
Non-germline *BRCA* mutation	112 (63)	53 (60)

ECOG: Eastern Cooperative Oncology Group; FIGO: International Federation of Gynecology and Obstetrics; SD: standard deviation.

As of the data cut-off date for this final analysis (14 August 2023), 138 (52.1%) patients (93 niraparib, 45 placebo) had experienced OS events (i.e., died), 15 (5.7%) patients (7 niraparib, 8 placebo) were lost to follow-up, 13 (4.9%) patients (7 niraparib, 6 placebo) withdrew from the study by their own requests, and six (2.3%) patients (3 niraparib, 3 placebo) discontinued the study due to unblinding for subsequent treatment decisions. Then, 93 (35.1%) patients (67 niraparib, 26 placebo) remained in the study, and 32 (12.1%) patients were still receiving treatment with niraparib (Fig. 1), with 31 from the niraparib group and one from the placebo group, who switched to niraparib after the study unblinding.

In the niraparib group, 17 (9.6%) patients (6 [9.2%] with germline *BRCA* mutations and 11 [9.8%] without germline *BRCA* mutations) received niraparib for five or more years. Subsequent anti-cancer therapies after discontinuation of the study treatment are summarised in Supplementary Table S2. In the overall population, 131 (74.0%) patients in the niraparib group and 77 (87.5%) patients in the placebo group received at least one dose of subsequent anti-cancer therapy, and 46 (26.0%) patients in the niraparib group and 41 (46.6%) patients in the placebo group received at least one dose of subsequent PARP inhibitor therapy. In those with germline *BRCA* mutations, 20 (30.8%) of the niraparib arm and 20 (57.1%) of the placebo arm received at least one dose of subsequent PARP inhibitor therapy; in those without, 26 (23.2%) of the niraparib arm and 21 (39.6%) of the placebo arm did so.

**Fig. 2:**
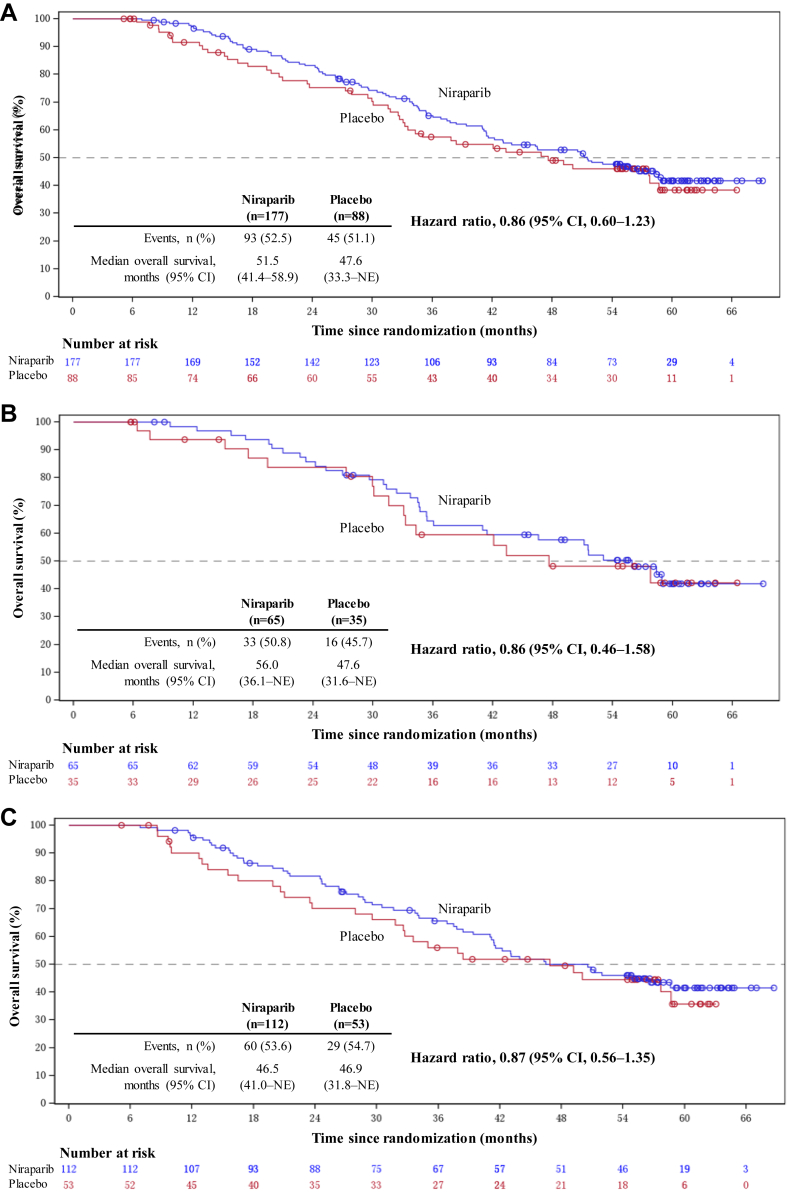
**Kaplan–Meier estimates of overall survival in (A) the overall population**∗**, (B) patients with germline *BRCA* mutations**^†^**, and (C) patients without germline *BRCA* mutations**^†^. ∗The following stratification factors were considered: germline *BRCA* mutation status, time to recurrence following the penultimate platinum-based chemotherapy, and response to the most recent platinum-based chemotherapy. ^†^The following stratification factors were considered: time to recurrence following the penultimate platinum-based chemotherapy and response to the most recent platinum-based chemotherapy. CI: confidence interval; NE: not evaluable.

At this final analysis, median follow-up duration (IQR) for OS was 57.9 months (54.8–61.6) in the overall population, 58.4 months (55.4–61.6) in the niraparib group, and 57.0 months (48.3–61.5) in the placebo group. Median OS in the overall population was 51.5 months (95% CI, 41.4–58.9) with niraparib and 47.6 months (33.3–not evaluable [NE]) with placebo (HR, 0.86; 95% CI, 0.60–1.23; Fig. 2A). In patients with germline *BRCA* mutations, median OS was 56.0 months (95% CI, 36.1–NE) with niraparib and 47.6 months (95% CI, 31.6–NE) with placebo (HR, 0.86; 95% CI, 0.46–1.58; Fig. 2B). In those without germline *BRCA* mutation, median OS was 46.5 months (95% CI, 41.0–NE) with niraparib and 46.9 months (95% CI, 31.8–NE) with placebo (HR, 0.87; 95% CI, 0.56–1.35; Fig. 2C). The OS results obtained from the per-protocol analysis (Supplementary Figure S1) are consistent with these results derived from the intention-to-treat analysis.

**Fig. 3:**
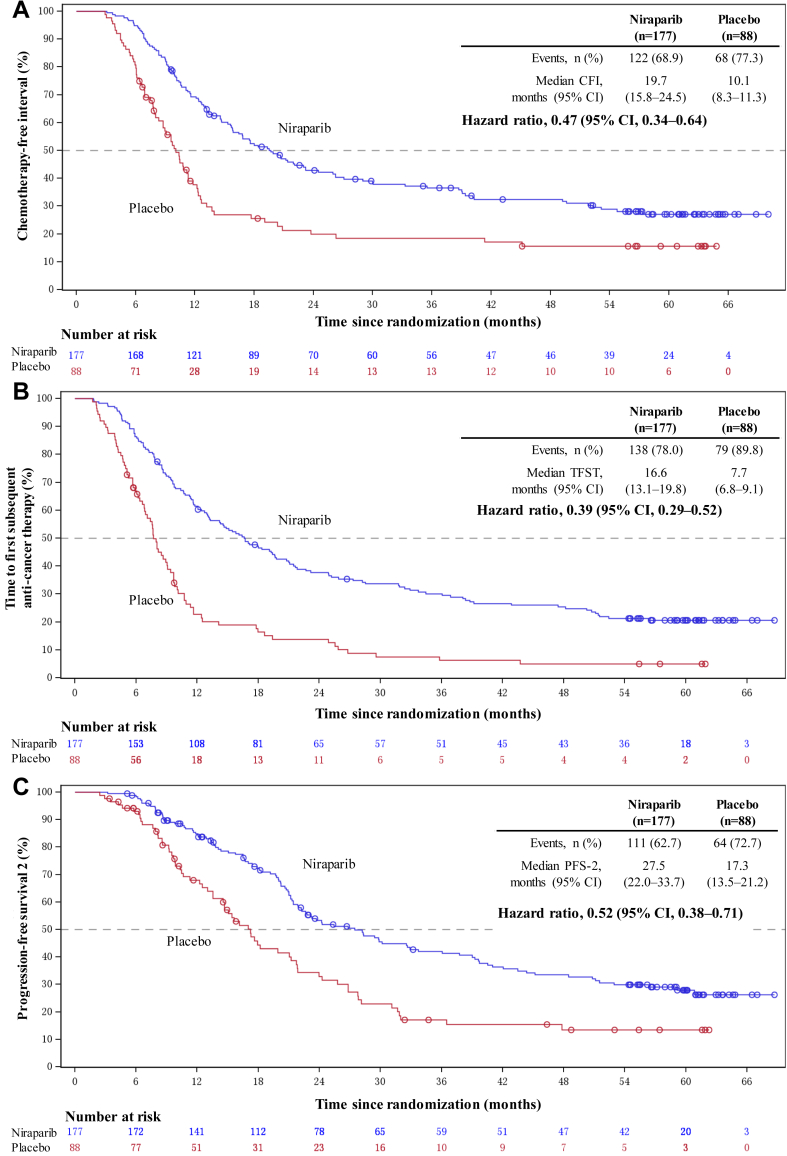
**Kaplan–Meier estimates of (A) chemotherapy-free interval**∗**, (B) time to first subsequent anti-cancer therapy**∗**, and (C) progression-free survival 2 in the overall population**∗. ∗The following stratification factors were considered: germline *BRCA* mutation status, time to recurrence following the penultimate platinum-based chemotherapy, and response to the most recent platinum-based chemotherapy. CI: confidence interval; CFI: chemotherapy-free survival; PFS-2: progression-free survival 2; TFST: time to first subsequent anti-cancer therapy.

In the overall population, median CFI was 19.7 months (95% CI, 15.8–24.5) with niraparib and 10.1 months (95% CI, 8.3–11.3) with placebo (HR, 0.47; 95% CI, 0.34–0.64; Fig. 3A); median TFST was 16.6 months (95% CI, 13.1–19.8) with niraparib and 7.7 months (95% CI, 6.8–9.1) with placebo (HR, 0.39; 95%, CI 0.29–0.52; Fig. 3B); and median PFS-2 was 27.5 months (95% CI, 22.0–33.7) with niraparib and 17.3 months (95% CI, 13.5–21.2) with placebo (HR, 0.52; 95% CI, 0.38–0.71; Fig. 3C). The Kaplan–Meier estimations of CFI, TFST, and PFS-2 by germline *BRCA* mutation status are provided in Supplementary Figures S2–S4. In both patients with and without germline *BRCA* mutations, niraparib showed a favourable trend versus placebo in CFI, TFST, and PFS-2.

As of the data cut-off for this final analysis, the safety profile of niraparib remained unchanged compared with that at the primary analysis, with no new safety signals identified.^16^ MDS/AML was reported in three (1.7%) of 177 niraparib-treated patients and none of placebo-treated patients. One patient with germline *BRCA* mutations was diagnosed with MDS during niraparib treatment and received niraparib treatment for 30.5 months before the diagnosis. Another patient with germline *BRCA* mutations was diagnosed with AML during niraparib treatment and received niraparib treatment for 19.4 months before the diagnosis. One patient without germline *BRCA* mutations was diagnosed with MDS 14.5 months after treatment discontinuation. This patient received niraparib treatment for 36.7 months and received six cycles of subsequent platinum-based chemotherapy. Second primary malignancies other than MDS/AML occurred in three (1.7%) of 177 niraparib-treated patients (one case each for chronic eosinophilic leukaemia, gastric cancer, and rectal cancer) and three (3.4%) of 88 placebo-treated patients (one case each for ductal carcinoma in situ of left breast, gastric cancer, and rectal cancer). Chronic eosinophilic leukaemia was diagnosed in a patient with germline *BRCA* mutations during niraparib treatment, who received niraparib treatment for 50.5 months before the diagnosis.

## Discussion

In the phase 3 NORA trial among patients with PSROC who had received at least two prior lines of platinum-based chemotherapy, niraparib maintenance therapy with an individualised starting dose demonstrated a favourable OS trend compared with placebo (median OS, 51.5 versus 47.6 months; HR, 0.86; 95% CI, 0.60–1.23), although 46.6% of the placebo group received subsequent PARP inhibitor therapy. This favourable OS trend with niraparib versus placebo was observed in both patients with germline *BRCA* mutations (median OS, 56.0 versus 47.6 months; HR, 0.86; 95% CI, 0.46–1.58) and without (median OS, 46.5 versus 46.9 months; HR, 0.87; 95% CI, 0.56–1.35). Niraparib also showed favourable results versus placebo in intermediate outcomes including CFI, TFST, and PFS-2, regardless of germline *BRCA* mutation status.

Statistically significant OS improvement is difficult to demonstrate in ovarian cancer trials because much improved survival in this patient population in recent decades necessitates prolonged follow-up periods and large sample sizes for proper OS evaluation and crossovers and post-progression therapies further confound the interpretation of OS results.^20^, ^21^, ^22^, ^23^, ^24^ In fact, the Ovarian Cancer Consensus Conference of the Gynecologic Cancer InterGroupa has recommended that PFS, supported by additional measures of clinical benefits, rather than OS be the preferred endpoint when the expected median OS is longer than 12 months in patients with recurrent ovarian cancer.^23^ Therefore, our NORA study chose PFS as the primary endpoint and subsequently was not powered for OS analysis.

Notably, despite the facts that the NORA study was not designed to power OS analysis and a higher proportion of the placebo group received subsequent anti-cancer therapies (especially, 46.6% of the placebo group versus 26.0% of the niraparib group received subsequent PARP inhibitor therapy), a favourable OS trend with niraparib versus placebo was still demonstrated, regardless of germline *BRCA* mutation status. This provides further evidence for the OS benefit from niraparib maintenance therapy for PSROC. Moreover, niraparib also showed a favourable trend over placebo in intermediate outcomes (CFI, TFST, and PFS-2) in NORA, regardless of germline *BRCA* mutation status, further endorsing the association of niraparib maintenance therapy with better long-term prognosis in this population.

Across previous trials in the maintenance setting for PSROC, an OS trend in favour of PARP inhibitors (niraparib in NOVA, rucaparib in ARIEL-3, and olaparib in Study 19 and SOLO2) versus placebo has been consistently observed in patients with *BRCA* mutations, even without adjustment for post-progression therapies, despite varying study designs, changes in treatment landscape, and different study populations across studies.^7^, ^8^, ^9^, ^10^ In patients with germline *BRCA* mutations from the NORA study, niraparib, although not statistically significantly, reduced the risk of death versus placebo by 14%, which aligns with the findings from previous trials of PARP inhibitors.^7^, ^8^, ^9^, ^10^ Thus, the NORA study further attests to the OS benefit that PARP inhibitor maintenance therapy confers to patients with PSROC who have *BRCA* mutations.

Unlike in patients with *BRCA* mutations, OS results in those without *BRCA* mutations have appeared to be divergent across previous trials of PARP inhibitors (olaparib, niraparib, and rucaparib) in the maintenance setting for PSROC.^7^^,^^9^^,^^10^ In the phase 2 Study 19 trial, a favourable OS trend was observed with olaparib versus placebo (median OS, 24.5 versus 26.6 months; HR, 0.83; 95% CI, 0.55–1.24).^7^ However, in the phase 3 NOVA trial, niraparib did not lead to an OS benefit trend versus placebo (median OS, 31.0 versus 34.8 months; HR, 1.06; 95% CI, 0.81–1.37).^10^ Similarly, the phase 3 ARIEL-3 trial also failed to show a favourable OS trend with rucaparib versus placebo in patients without *BRCA* mutations.^9^ The reasons behind these inconsistent OS trends in patients without *BRCA* mutations may warrant further investigation.

In our NORA study, niraparib demonstrated a favourable OS trend versus placebo in patients with PSROC who had no *BRCA* mutations. Compared with the phase 3 NOVA trial, two pivotal features of the NORA trial may contribute to this observed OS benefit trend. Firstly, compared with NOVA, the tolerability profile of niraparib was improved in NORA due to the use of the individualised starting dose strategy, as indicated by the rates of treatment discontinuation due to treatment-emergent adverse events (TEAEs) in these two studies.^14^^,^^16^ Although the proportion of the placebo group who discontinued treatment due to TEAEs was slightly higher in NORA than in NOVA (5.7% versus 2.2%) at the primary analysis, treatment discontinuation due to TEAEs in the niraparib group occurred much less frequently (4.0% versus 14.7%) in NORA than in NOVA.^14^^,^^16^ The improved tolerability profile of niraparib in NORA may lead to better compliance, longer treatment duration, and thereby increased exposure. Increased exposure to PARP inhibitors is associated with better efficacy,^25^, ^26^, ^27^, ^28^ especially in patients without *BRCA* mutations.^25^^,^^26^^,^^29^ Furthermore, all patients without germline *BRCA* mutations from NORA received two and only two prior lines of chemotherapy, whereas approximately one third (33.4%, 117/350) of patients without germline *BRCA* mutations in NOVA received three or more prior lines of chemotherapy.^14^ The earlier use of PARP inhibitor maintenance therapy has been shown to be associated with greater OS benefit in the recurrence setting.^8^

Since December 2022, the United States Food and Drug Administration has restricted the indications of PARP inhibitors (niraparib, rucaparib, and olaparib) as maintenance therapy for PSROC to patients with *BRCA* mutations only.^11^, ^12^, ^13^ However, the indications of these PARP inhibitors as maintenance therapy for PSROC have not changed in European Union, China, and Japan among others. Specifically, the European Medicines Agency has reviewed OS results from NOVA and kept indications of niraparib intact,^30^^,^^31^ indicating that patients without *BRCA* mutations are still perceived to benefit from prolonged PFS time with niraparib maintenance therapy. Our final analysis of the NORA trial, which demonstrates a favourable OS trend with niraparib versus placebo, regardless of germline *BRCA* mutation status, provides further evidence supporting the use of niraparib maintenance therapy in both patient populations with and without *BRCA* mutations in the recurrence setting and adds more weight to the argument that patients without *BRCA* mutations can also receive an OS benefit from PARP inhibitor maintenance therapy.

This study has some limitations. Firstly, the homologous recombination status was not assessed because no commercial HRD assay was available in China at that time. Secondly, we did not evaluate patient-reported outcomes, which would have been helpful to understand the impact of niraparib treatment on patients’ quality of life. Thirdly, this trial enrolled Asian patients exclusively, which may affect the generalisability of its findings to other ethnic groups. However, it should be noted that race does not significantly affect the pharmacokinetics of niraparib.^12^^,^^32^

In summary, the NORA trial demonstrated a significant PFS improvement and a favourable OS trend with niraparib versus placebo using an individualised starting dose as maintenance therapy in patients with PSROC, regardless of germline *BRCA* mutation status. Similarly, niraparib showed a favourable trend versus placebo for the intermediate outcomes of CFI, TFST, and PFS-2 in this patient population. These favourable results in PFS and OS as well as other intermediate outcomes associated with niraparib continue to support the use of niraparib maintenance therapy for all-comer population in the recurrence setting.

## Contributors

CZ, JMH, MRM, and XHW were involved in study concept and design. ZH, JD, CZ, JMH, MRM, and XHW contributed to data curation, formal analysis, and development of study methodology. Provision of study materials or patients, data collection: XHW, JQZ, RTY, JXY, JHL, JW, LYW, ZLL, YNG, DBW, GL, HYY, QZ, BHK, YH, LPC, GLL, RFA, KW, YZ, XJY, XL, WGL, MH, LW, HC, QHC, GA, XHH, XFT, and HW. All authors were involved in review and editing of the manuscript, and data interpretation. XHW and JMH had directly accessed and verified the underlying data reported in the manuscript. All authors had full access to all the data in the study and had final responsibility for the decision to submit for publication.

## Data sharing statement

De-identified individual participant data that underline the results reported in this article will be made available to researchers who provide a methodologically sound proposal to achieve aims stated in the proposal upon approval. Proposals should be directed to the corresponding author and approved by the study sponsor.

## Declaration of interests

ZH, JD, CZ, and JMH report employment with and hold shares and stock options in Zai Lab. MRM is a member of board of directors and holds stocks/shares of Karyopharm Therapeutics and Sera Prognostics and has participated in advisory boards for Allarity Therapeutics, AstraZeneca, Biocad, Biontech, Boehringer Ingelheim, Clovis, Daiichi-Sankyo, Eisai, Genmab, GSK, Immunogen, Incyte, Karyopharm, Merck/MSD, Mersana, Novartis, Regeneron, Roche, SeaGen, Takeda, Tesaro, and Zai Lab. All other authors declare no competing interest.

## Appendix A Supplementary data

Supplementary Materials

Trial protocol
