# Supplementary material for: Niraparib maintenance therapy using an individualised starting dose in patients with platinum-sensitive recurrent ovarian cancer (NORA): final overall survival analysis of a phase 3 randomised, placebo-controlled trial
Source: eClinicalMedicine. 2024 May 7;72:102629. doi: 10.1016/j.eclinm.2024.102629 (PMC11090914; doi:10.1016/j.eclinm.2024.102629)
Supplement: Supplementary Materials [file mmc1.pdf]

Niraparib maintenance therapy using an individualised starting dose in patients with platinum-sensitive recurrent ovarian cancer (NORA): Final overall survival analysis of a phase 3 randomised, placebo-controlled trial

Contents

Supplementary methods 2

- Full eligibility criteria 2
  - Inclusion criteria 2
  - Exclusion criteria 2
- Criteria for treatment discontinuation and study discontinuation 3
  - Treatment discontinuation 3
  - Study discontinuation 3
- Dose modifications for the management of adverse events 4
- Supplementary Table S1: Dose adjustment scheme for ZL-2306 (Niraparib). 5

Supplementary Results 6

- Supplementary Table S2: Subsequent anti-cancer therapies in the overall population. 6
- Supplementary Figure S1: Kaplan-Meier estimates of overall survival in (A) the overall population (per protocol), (B) patients with germline *BRCA* mutations (per protocol), and (C) patients without germline *BRCA* mutations (per protocol). 7
- Supplementary Figure S2: Kaplan-Meier estimates of chemotherapy-free interval in (A) patients with germline *BRCA* mutations and (B) patients without germline *BRCA* mutations. 8
- Supplementary Figure S3: Kaplan-Meier estimates of time to first subsequent anti-cancer therapy in (A) patients with germline *BRCA* mutations and (B) patients without germline *BRCA* mutations. 9
- Supplementary Figure S4: Kaplan-Meier estimates of progression-free survival 2 in (A) patients with germline *BRCA* mutations and (B) patients without germline *BRCA* mutations. 10

Supplementary methods

Full eligibility criteria

Inclusion criteria

Enrolled patients must meet all of the following criteria:

1. Written informed consent before any study-related procedure.
2. Agree to receive g*BRCA* mutation test (result must be known before randomization).
3. Women aged 18 years or older.
4. Histologically confirmed epithelial ovarian cancer, fallopian tube cancer or primary peritoneal cancer.
5. High-grade (Grade 3) serous or predominantly high-grade serous ovarian cancer (no histological restrictions for patients with ovarian cancer carrying germline BRCA mutations).
6. Having received at least two lines of platinum-based chemotherapy, and meet following criteria:
   a. After the penultimate platinum-based chemotherapy, the patient shall meet the following requirements:
      - Clinically CR or PR to this line of chemotherapy.
      - Relapse after this course of chemotherapy must be PSROC, i.e., disease does not progress until 6 months after the course of chemotherapy (on medical records 6-12 months or ≥ 12 months).

   b. After the most recent line of platinum-based chemotherapy, the patient shall meet the following requirements:
      - Having received at least 4 cycles of platinum-containing chemotherapy (must be carboplatin or cisplatin or nedaplatin).
      - Clinical response to this line of chemotherapy must be CR (i.e., after this line of chemotherapy, there is no imaging measurable or unmeasurable lesion according to RECIST v1.1, and CA-125 is within the normal range) or PR (after this line of chemotherapy at least 30% reduction in total sum of diameter of targe lesion compared to the baseline before chemotherapy).
      - CA-125 level is within the normal range after the chemotherapy, or decreases > 90% during the course of chemotherapy and remains stable for at least 7 days (increase in CA-125 level before enrollment should not exceed 15% relative to the level after chemotherapy).
      - No measurable lesion> 2cm (measurable disease >2cm shall be discussed with the sponsor based on evidence of PR).

   c. The patient shall be randomized within 8 weeks after completion of most recent line platinum-containing chemotherapy.
7. ECOG performance status 0 or 1.
8. Adequate organ function, including
   a. Neutrophil count ≥1500/µL
   b. Platelet count ≥100,000/µL
   c. Hemoglobin ≥10g/dL
   d. Serum creatinine ≤ 1·5 × ULN, or creatinine clearance rate ≥ 60mL/min (using Cockcroft-Gault equation)
   e. Total bilirubin ≤1·5 × ULN, or direct bilirubin ≤1·0 × ULN.
   f. AST and ALT ≤ 2·5 × ULN, or ≤ 5 × ULN in case of hepatic metastases.
9. Women of childbearing age with a negative pregnancy test at the time of enrollment and have promised to take adequate and effective contraceptive measures or abstinence during the study period and within 3 months after the last dose of study drug. Or women without potential fertility, defined as below:
   a. Having received surgical sterilization operation (such as hysterectomy, bilateral ovariectomy, or bilateral tubectomy); or
   b. ≥60 years of age; or
   c. ≥40 and <60 years of age, more than 12 months of menopause, and follicle-stimulating hormone (FSH) are within the post-menopause reference range.
10. Ability to comply with the protocol.
11. The level of any toxicity due to previous chemotherapy has decreased to ≤CTCAE grade 1 or baseline level, except for ≤CTCAE grade 2 stable sensory neuropathy or alopecia.

Exclusion criteria

A patient shall be excluded from the study if any one of following criteria is met:

1. Known hypersensitivity to active or inactive components of ZL-2306 (niraparib) or drugs with similar chemical structures.
2. Previous treatment with PARP inhibitor.

3. Drainage of ascites during last 2 cycles of last chemotherapy prior to enrollment
4. Symptomatic uncontrollable brain or leptomeningeal metastases. No imaging scan is required to confirm the absence of brain metastases; patients with spinal cord compression who have received targeted treatment and evidence of clinical stability of the disease for at least >28 days can still be considered for enrollment (in case of controlled central nervous system metastasis, relevant treatment such as radiotherapy or chemotherapy must be given at least 1 month before the study; no new CNS-related symptoms or symptoms indicating disease progression, and either are taking a stable dose of steroid, or do not need hormone therapy).
5. Having received a major surgery 3 weeks before entering the study, or not yet recovered from the surgical effect.
6. Having received a palliative radiotherapy covering >20% of bone marrow within 1 week prior to enrollment.
7. Invasive cancers other than ovarian cancer within 2 years prior to enrollment (except for fully treated basal or squamous cell skin cancer).
8. A previous or current diagnosis of myelodysplastic syndrome (MDS) or acute myeloid leukemia (AML).
9. Other serious or uncontrolled diseases, including but not limited to:
    a. Uncontrollable nausea and vomiting, inability to swallow study drug, and any gastrointestinal disease that may interfere with the absorption and metabolism of the drug
    b. Active viral infections, such as human immunodeficiency virus, hepatitis B virus, hepatitis C virus and so on
    c. Uncontrolled ventricular arrhythmias and recent myocardial infarction within 3 months
    d. Uncontrolled major seizure disorder, unstable spinal cord compression, superior vena cava syndrome or any other psychiatric disorders that prohibits obtaining informed consent
    e. Immunodeficiency (except for splenectomy), or other diseases that investigators believe may expose patients to high-risk toxicity.
10. Any previous or current disease, treatment or lab abnormality that might interfere with the results of the study, the patient’s full participation during the study, or the investigator believes that the patient is not suitable for participating in this study; platelet or red blood cell transfusion is not allowed within four weeks before initiation of study treatment.
11. Pregnancy or breast feeding, or planning a pregnancy during the study.

Criteria for treatment discontinuation and study discontinuation

Treatment discontinuation

Patients may be discontinued from treatment for the following reasons:

- Adverse event
    - Any treatment-related CTCAE grade-3/4 adverse events, which have not reverted to CTCAE grade 1 or lower within 4 weeks (28 days) of dose interruption
    - Dose reduction is at the investigator's discretion, and must comply with the dose interruption regulations (≤28 days); the maximum number of 2 does reduction is allowed. If any CTCAE grade-3/4 adverse events still recur at the lowest allowable dose, the investigator must discuss with the sponsor whether treatment should be discontinued according to the patient's condition.
    - If the adverse event is thrombocytopenia, and the platelet count have not reverted to >100,000/µL within 4 weeks (28 days) of dose interruption, the patient must be discontinued;
- Disease progression as assessed according to RECIST 1.1 criteria
- Risk to patient as judged by the investigator and/or Sponsor
- Serious violation of the study protocol as judged by the investigator and/or Sponsor
- Patient request
- The patient becomes pregnant

The patients who discontinue from the treatment due to reasons listed above will continue to receive follow-up assessments (such as CFI, TFST and OS), unless the patients discontinued from the study because of the conditions listed below.

Study discontinuation

All subjects may discontinue from this study at any time, regardless of whether a reason is provided or not; and they will not be discriminated or unfairly treated, and their medical treatment will not be affected.
Patients who discontinue from the study under one of following conditions will not be subject to the further data collection:

- Withdrawal of informed consent

- Death by any cause
- Lost to follow-up

Subjects should complete the end-of-therapy visit (EOT) when they discontinue study treatment or withdraw from the study. The research center shall immediately inform the inspector of the Sponsor and record the date and cause of treatment discontinuation and the withdrawal from study.

If a patient withdraws voluntarily early and does not want to return to the site to complete the final visit, the investigator should make every effort to contact the patient. The investigator shall ask the reason for the early withdrawal, and if feasible, request them to return all unused study drugs and supplies as much as possible and to complete the last visit, as well as follow up and treat any unresolved adverse events.

In any case, the patient's final condition shall be recorded as comprehensively as possible, especially the patient's survival status (secondary study endpoint).

If a patient withdraws from the trial and requests not to disclose their future information, no examination is necessary. However, the Sponsor may still retain and use the pre-existing information.

The study will not replace subjects in the event of any early withdrawal.

Dose modifications for the management of adverse events

The patients will be randomized to receive ZL-2306 (niraparib) or matched placebo in a 2: 1 ratio. The initial dose is determined based on the subject's baseline body weight or baseline platelet counts (but dose modification after initiation will not be based on body weight):

- For patients with baseline body weight ≥77 kg and baseline platelet count ≥150,000 /µL, the starting dose is ZL-2306 (niraparib) 300 mg (3 × 100 mg capsules) or matched dose of placebo (3 capsules);
- For patients with baseline body weight <77 kg or baseline platelet count <150,000/µL, the starting dose is ZL-2306 (niraparib) 200 mg (2 × 100 mg capsules) or matched dose of placebo (2 capsules). For patients with a starting dose of 200 mg (2 capsules), if there is no dose interruption or reduction during the first two cycles of treatment, the dose can be adjusted to 300 mg (3 capsules).

Each subject will orally take the prescribed dose of ZL-2306 (Niraparib) or matched placebo once a day; the number of capsules taken can be adjusted accordingly for any dose modification during the study. It is recommended to take the medicine at the same time every day, preferably in the morning, with water or food. The entire capsule shall be swallowed, not broken, chewed or opened; eating and drinking are allowed before and after taking the medicine. The patients should be informed that if they missed the planned dose (for example, if they forgot to take it) or vomited after taking the drug, they should not take the drug again, but take it at the next scheduled time. Any study drug intteruption/discontinuation and dose reduction as well as their reasons shall be recorded in detail in the eCRF.

In the event of an adverse event caused by the trial treatment, the investigator can modify the dosage according to the dose adjustment principle specified in the protocol, that is, temporary interruption (up to 28 days) or dose reduction (as shown in **Table S1**).

- For patients with an initial dose of 300 mg/d, the dose can be reduced to 200 mg/d or further reduced to 100 mg/d, which is the minimum dose and no further reduction is allowed (unless the investigator believes that the patient may continue to benefit from further reduction, and it requires permission from the sponsor's medical monitor);
- For patients with an initial dose of 200 mg/d, it can be reduced to 100 mg/d which is the minimum dose and no further reduction is allowed (unless the investigator believes that the patient may continue to benefit from further reduction, and it requires permission from the sponsor's medical monitor).

Supplementary Table S1: Dose adjustment scheme for ZL-2306 (Niraparib).

Dose level	Starting dose: 300 mg QD	Starting dose: 200 mg QD
Initial dose level	300 mg QD	200 mg QD[1]
Initial dose level -1	200 mg QD[3]	100 mg QD[2,3]
Initial dose level -2	100 mg QD[2,3]	NA

[1] For patients with a starting dose of 200 mg, on the premise that there is no dose interruption or dose reduction due to adverse reactions in the first two cycles of treatment, the starting dose may be increased to 300 mg.

[2] If an adverse reaction requiring dose modification occurs at the 100 mg dose level, unless the investigator believes that the patient may continue to benefit from continued medication or further reductions, and permission from the sponsor’s medical monitor is required.

[3] After reduction of the study drug from 200 mg QD to 100 mg QD, raising dose to 200 mg QD can be considered if well tolerated for at least one treatment cycle. After the study drug is reduced from the initial dose of 300 mg QD to 200 mg QD, the dose may be raised back to 300 mg QD if well tolerated for at least 2 cycles. Any increase in the dose of study drug after the dose reduction should be discussed with the sponsor’s medical monitor.

Supplementary Results

Supplementary Table S2: Subsequent anti-cancer therapies in the overall population.

Subsequent anti-cancer therapy, n (%)	Niraparib (n=177)	Placebo (n=88)	Total (n=265)
At least one subsequent anti-cancer therapy	131 (74·0)	77 (87·5)	208 (78·5)
At least one surgery	28 (15·8)	18 (20·5)	46 (17·4)
At least one radiotherapy	8 (4·5)	7 (8·0)	15 (5·7)
At least one systemic therapy	129 (72·9)	76 (86·4)	205 (77·4)
At least one dose of PARP inhibitor[a]	46 (26·0)	41 (46·6)	87 (32·8)
At least one dose of bevacizumab	43 (24·3)	22 (25·0)	65 (24·5)
At least one dose of chemotherapy	122 (68·9)	68 (77·3)	190 (71·7)
At least one dose of targeted therapy[b]	28 (15·8)	17 (19·3)	45 (17·0)
At least one dose of immunotherapy[c]	9 (5·1)	3 (3·4)	12 (4·5)
At least one dose of endocrine therapy[d]	4 (2·3)	2 (2·3)	6 (2·3)
At least one dose of antibody drug conjugates	1 (0·6)	0	1 (0·4)

[a] PARP inhibitor therapies includes olaparib, niraparib, fluzoparib and pamiparib. [b] Targeted therapy includes apatinib, anlotinib, lapatinib and lenvatinib. [c] Immunotherapy includes toripalimab, pembrolizumab, camrelizumab and sintilimab. [d] Endocrine therapy includes tamoxifen, letrozole and megestrol. PARP=poly(adenosine diphosphate-ribose) polymerase.

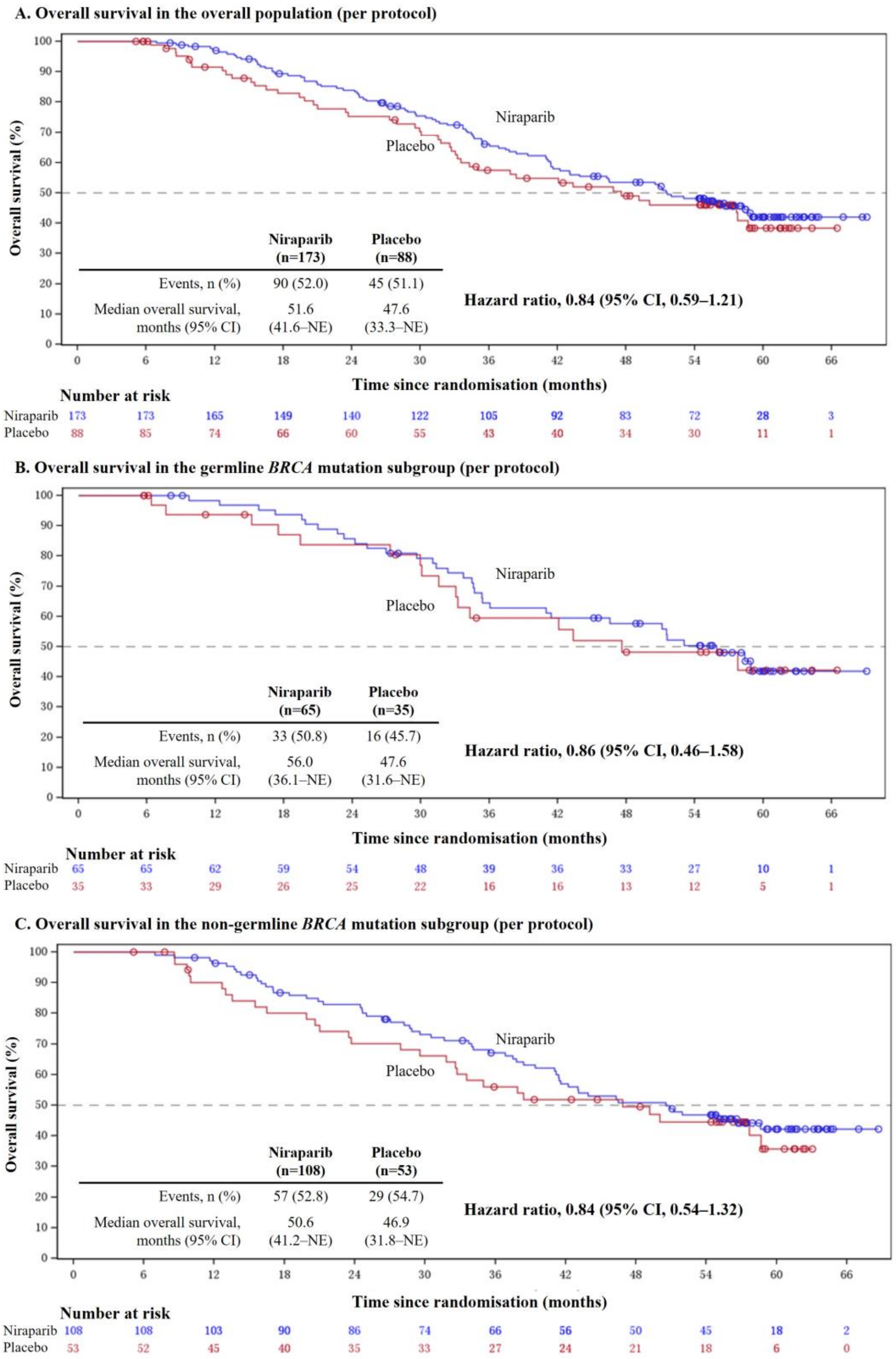

Supplementary Figure S1: Kaplan-Meier estimates of overall survival in (A) the overall population (per protocol)*, (B) patients with germline *BRCA* mutations (per protocol)†, and (C) patients without germline *BRCA* mutations (per protocol)†.

*The following stratification factors were considered: germline *BRCA* mutation status, time to recurrence following the penultimate platinum-based chemotherapy, and response to the most recent platinum-based chemotherapy. †The following stratification factors were considered: time to recurrence following the penultimate platinum-based chemotherapy and response to the most recent platinum-based chemotherapy. CI: confidence interval; NE: not evaluable.

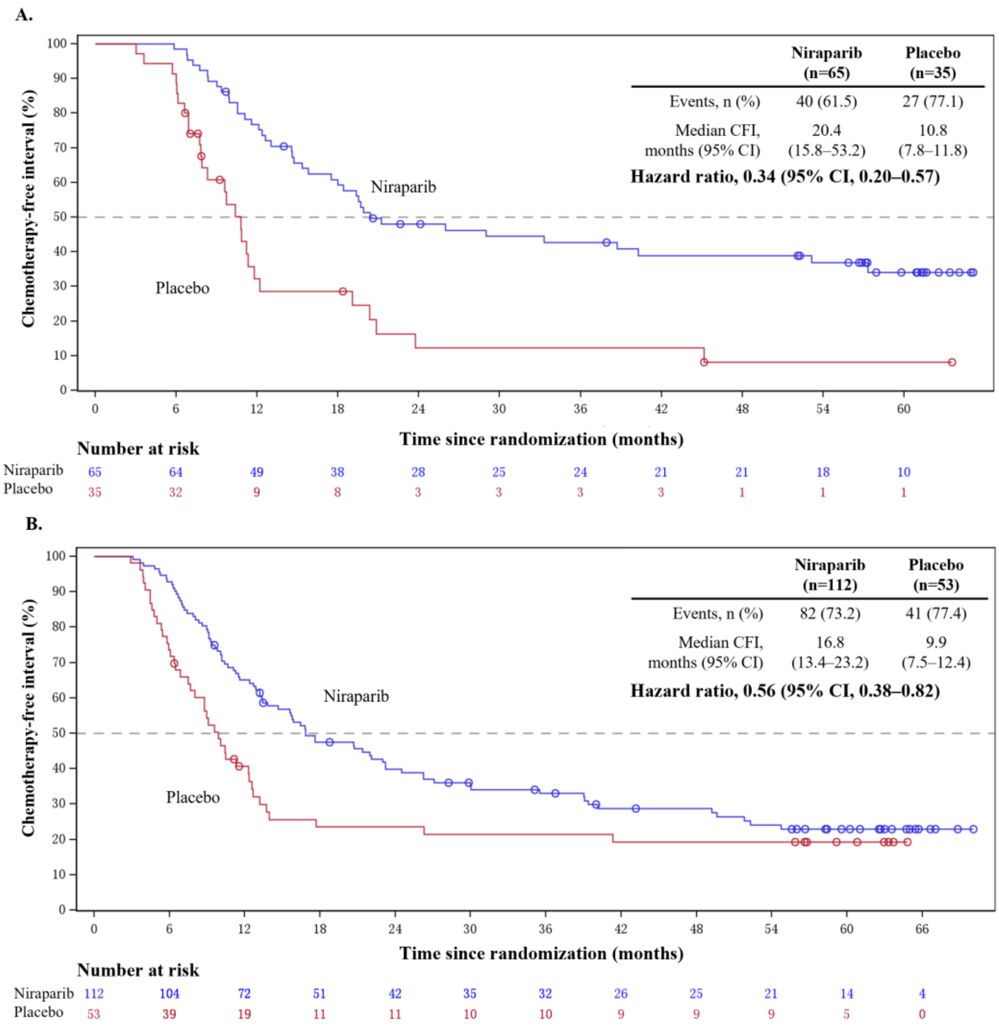

Supplementary Figure S2: Kaplan-Meier estimates of chemotherapy-free interval in (A) patients with germline *BRCA* mutations[*] and (B) patients without germline *BRCA* mutations[*].

[*]The following stratification factors were considered: time to recurrence following the penultimate platinum-based chemotherapy and response to the most recent platinum-based chemotherapy.
CI: confidence interval; CFI: chemotherapy-free interval.

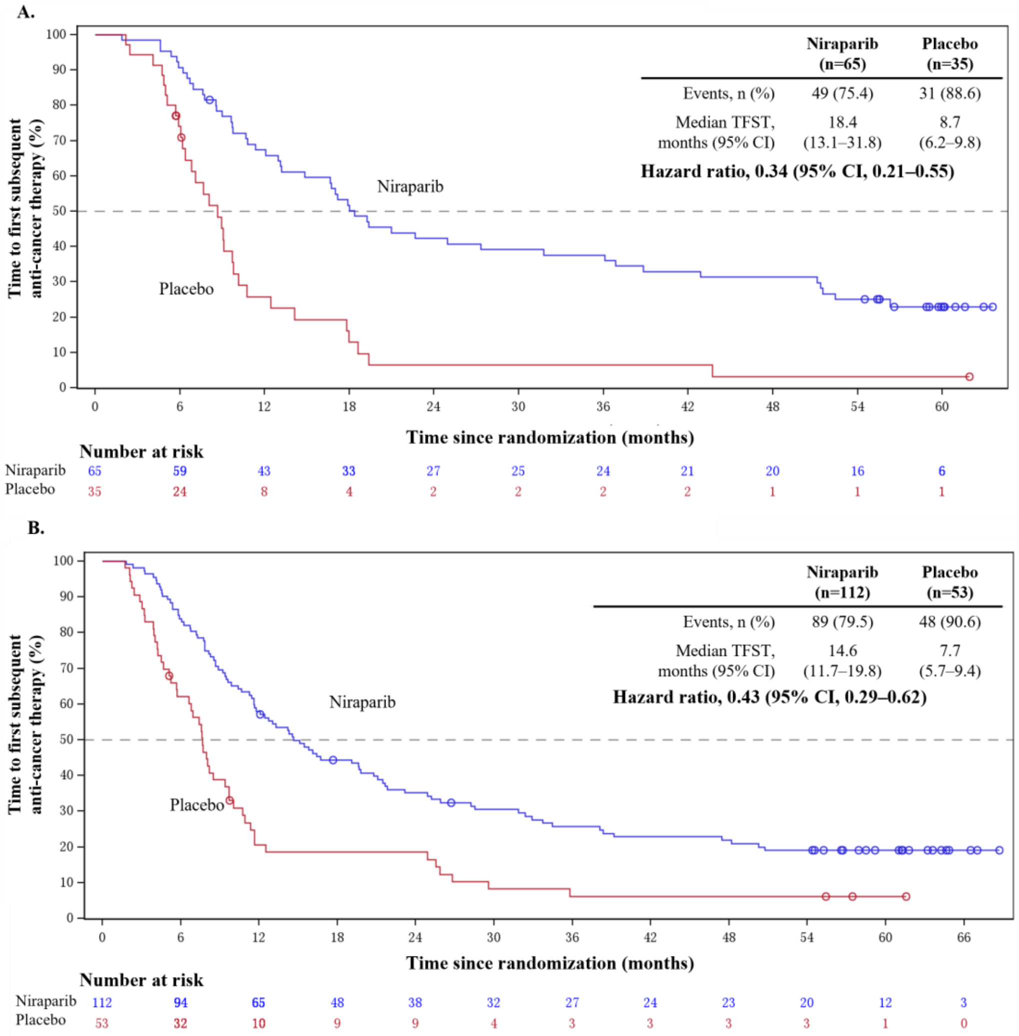

Supplementary Figure S3: Kaplan-Meier estimates of time to first subsequent anti-cancer therapy in (A) patients with germline *BRCA* mutations* and (B) patients without germline *BRCA* mutations*.

*The following stratification factors were considered: time to recurrence following the penultimate platinum-based chemotherapy and response to the most recent platinum-based chemotherapy.

CI: confidence interval; TFST: time to first subsequent anti-cancer therapy.

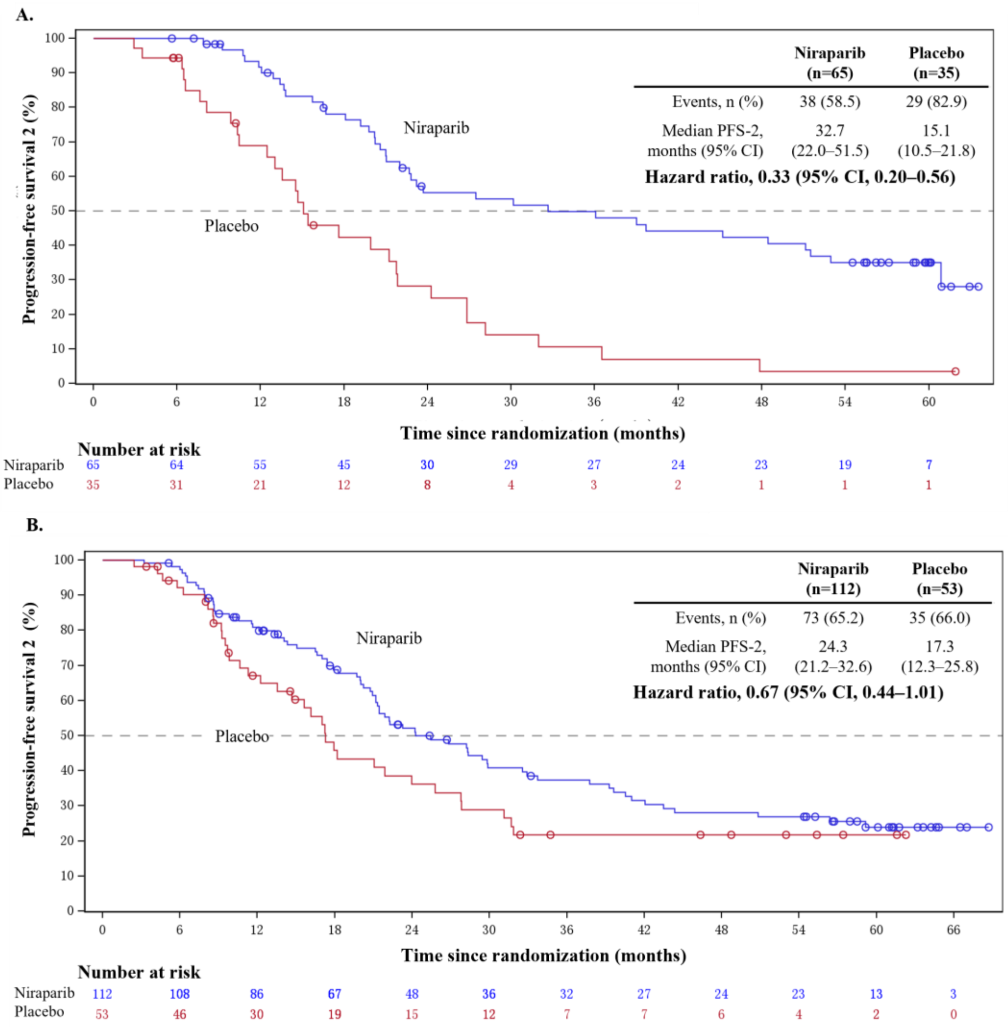

Supplementary Figure S4: Kaplan-Meier estimates of progression-free survival 2 in (A) patients with germline *BRCA* mutations* and (B) patients without germline *BRCA* mutations*.

*The following stratification factors were considered: time to recurrence following the penultimate platinum-based chemotherapy and response to the most recent platinum-based chemotherapy.

CI=confidence interval. PFS-2=progression-free survival 2.
