# Supplementary material for: Niraparib maintenance therapy using an individualised starting dose in patients with platinum-sensitive recurrent ovarian cancer (NORA): final overall survival analysis of a phase 3 randomised, placebo-controlled trial
Source: eClinicalMedicine. 2024 May 7;72:102629. doi: 10.1016/j.eclinm.2024.102629 (PMC11090914; doi:10.1016/j.eclinm.2024.102629)
Supplement: Trial protocol [file mmc2.docx]

Clinical Study Protocol

| Study Title: | A Phase III, Randomized, Double-Blind, Placebo-Controlled, Multicenter Study on Efficacy and Safety of ZL-2306 (niraparib) as Maintenance Treatment for Patients with Platinum-Sensitive Recurrent Ovarian Cancer, Carcinoma of Fallopian Tube or Primary Peritoneal Cancer (collectively referred to as Ovarian Cancer) |
| --- | --- |
| Project No.: | ZL-2306-001 |
| Version No. and Version Date: | Feb 10, 2020/ version 5.0 |
| Study Drug Name: | ZL-2306 (niraparib) |
| Study Phase: | Pivotal Study |
| Sponsor: | Zai Lab (Shanghai) Co., Ltd. |
| Signature of Sponsor: |  |
| Principal Investigator: | Professor Wu Xiaohua Fudan University Shanghai Cancer Center |

Confidentiality Declaration

Confidential information on the study drug in this study is the properties of Zai Lab (Shanghai) Co., Ltd. This document is intended only for review by investigators, study consultants or relevant personnel, institutional review board/independent ethics committee. It is not permitted to disclose any content of this document to any third party, without prior approval in written from the Sponsor.

**Signature Page**

**Protocol Title:** A phase III, randomized, double-blind, placebo-controlled, multicenter study on efficacy and safety of ZL-2306 (niraparib) as maintenance treatment for patients with platinum-sensitive recurrent ovarian cancer, carcinoma of fallopian tube or primary peritoneal cancer (collectively referred to as Ovarian Cancer)

**Clinical Study Protocol No.:** ZL-2306-001

I have read and agreed with the whole content of this protocol.

I have agreed that:

- I will endeavor to perform the study strictly according to the study protocol, guidelines on Good Clinical Practice and all other applicable laws and regulations.
- I will properly keep all information provided by Zai Lab (Shanghai) Co., Ltd. in accordance with the confidentiality requirements. When the information needs to be submitted to the Institutional Review Board (IRB) or the Independent Ethics Committee (IEC), it is necessary to indicate that the information is confidential, to ensure that confidential information contained in this document will not be used for any purpose other than clinical study assessment or implementation.

|  |  |  |  |  |  |
| --- | --- | --- | --- | --- | --- |
|  |  |  |  |  |  |
|  | Yongjiang Hei |  |  |  |  |
|  | < Name of Sponsor> |  | Signature |  | Date |
|  |  |  |  |  |  |
|  |  |  |  |  |  |
|  | Chief Medical Officer |  |  |  |  |
|  | <Title> |  |  |  |  |
|  |  |  |  |  |  |
|  |  |  |  |  |  |
|  |  |  |  |  |  |
|  |  |  |  |  |  |
|  | <Name of Principal Investigator > | | Signature |  | Date |
|  |  |  |  |  |  |
|  |  |  |  |  |  |
|  |  |  |  |  |  |
|  | <Title> |  |  |  |  |

**Summary of Protocol**

| Sponsor/Company Name: | Zai Lab (Shanghai) Co., Ltd. |
| --- | --- |
| Study Drug: | ZL-2306 (niraparib) |
| Active Ingredient: | ZL-2306 (niraparib) p-toluenesulfonate monohydrate |
| **Title:** | A phase III, randomized, double-blind, placebo-controlled, multicenter study on efficacy and safety of ZL-2306 (niraparib) as maintenance treatment for patients with platinum-sensitive recurrent ovarian cancer, carcinoma of fallopian tube or primary peritoneal cancer (collectively referred to as Ovarian Cancer) |
| **Protocol No.:** | ZL-2306-001 |
| **Principal Investigator:** | Professor Wu Xiaohua |
| **Study Sites:** | Approximately 30 Sites |
| **Study Duration:** | 3 years |
| **Study Stage:** | Pivotal Study |
| **Study Objectives:**  Primary Objective:  The primary objective of this study is to evaluate the efficacy of ZL-2306 (niraparib) as maintenance treatment for platinum-sensitive recurrent (PSR) ovarian cancer (OC) by comparing progression-free survival (PFS) between ZL-2306 (niraparib) and placebo.  Secondary Objective:  The secondary objective of this study is to evaluate additional clinical benefits compared to placebo, including chemotherapy-free interval (CFI), time to first subsequent treatment (TFST), overall survival (OS), safety and tolerability. | |
| **Study Design:**  This phase III, 2:1 randomized, double-blind, placebo-controlled, multicenter clinical trial, is designed to evaluate the efficacy and safety of ZL-2306 (niraparib) as maintenance treatment for patients with PSR ovarian cancer, fallopian tube cancer or primary peritoneal cancer (collectively referred to as Ovarian Cancer). The subjects enrolled shall meet the following inclusion criteria:   - Histologically diagnosed high-grade serous or predominantly high-grade serous epithelial OC (no histological restrictions for OC patients harboring germline BRCA mutations); - Having received at least 2 prior lines of platinum-based chemotherapy and achieved response to the penultimate line platinum-based regimen (CR or PR) but relapsed after 6 months (platinum sensitive recurrence); - Having received at least 4 cycles of last-line (the most recent line) platinum-based (must be carboplatin or cisplatin or nedaplatin) combination after relapse and achieved response (CR or PR), without any measurable lesion > 2cm (eligibility of lesion > 2cm shall be discussed with the sponsor based on evidence of PR), and CA-125 shall be within the normal range (or decreased by more than 90% and remained stable for at least 7 days) following the most recent platinum based chemotherapy.   The primary objective of this study is to evaluate the efficacy of ZL-2306 (niraparib) as maintenance treatment for PSROC by comparing the difference in tumor progression-free survival (PFS) between ZL-2306 (niraparib) and placebo. A total of 240 PSROC patients will be randomized to receive ZL-2306 (niraparib) or placebo in a 2:1 ratio.  Subjects are required to submit peripheral blood samples before randomization for detection of g*BRCA* mutation in a designated central laboratory. g*BRCA* mutation status will be taken as one of the factors for randomized stratification, and others include time to relapse following penultimate platinum (relapse after 6-12 months, or ≥12 months), and response to the most recent platinum (CR or PR).  After randomization, the subjects will begin maintenance treatment with ZL-2306 (niraparib) or matched placebo on the first day of the first cycle. The initial dose is determined based on the subject's baseline weight and platelet count (but dose modification after initiation will not be based on body weight):   - For patients with baseline body weight ≥77 kg and baseline platelet count ≥150 × 103/µL, the starting dose is ZL-2306 (niraparib) 300 mg (3× 100 mg capsules) or matched dose of placebo (3 capsules); - For patients with baseline body weight <77 kg or baseline platelet count <150 × 103/µL, the starting dose is ZL-2306 (niraparib) 200 mg (2× 100 mg capsules) or matched dose of placebo (2 capsules). For patients with a starting dose of 200 mg (2 capsules), if there is no dose interruption or dose reduction during the first two cycles of treatment, the dose can be adjusted to 300 mg (3 capsules).   The drug will be taken orally before or after meal (at the same time of the day if possible) once daily (QD); a study treatment cycle is defined as continuous administration for 28 days. Patients will continue to receive the assigned treatment until disease progression based on the Response Evaluation Criteria in Solid Tumors [RECIST v1.1] criteria, death, intolerable toxicity, withdrawal of consent or lost to follow-up.  Treatment interruption or dose reduction due to any grade of adverse reaction is allowed at any time during the study; but the case will be withdrawn from the study if continuous drug interruption is more than 28 days. Dose modification for adverse reaction is allowed:   - For patients with an initial dose of 300mg/d, it can be reduced to 200mg/d or further reduced to 100mg/d, which is the minimum dose and no further reduction is allowed (unless the investigator believes that the patient may continue to benefit from further reduction, and it requires permission from the sponsor’s medical monitor); - For patients with an initial dose of 200mg/d, it can be reduced to 100mg/d which is the minimum dose and no further reduction is allowed (unless the investigator believes that the patient may continue to benefit from further reduction, and it requires permission from the sponsor’s medical monitor).   The evaluation of efficacy and safety will be conducted according to the visit schedule, and the time frame will not be affected by dose interruption or reduction.  Clinical visits are arranged for each treatment cycle. Contrast-enhanced CT or MRI scans of the subject's abdomen / pelvis and clinically indicated sites will be performed and evaluated based on the RECIST v1.1 guidelines. The imaging evaluations are scheduled at the end of every 2 cycles (8 weeks ± 7 days) till the end of the 14^th^ cycle (56 weeks), and then at the end of every 3 cycles until disease progression. The tumor imaging evaluation shall be performed in accordance with the time frames specified above, and the counting of treatment cycles will not be affected by any treatment interruption. If the end of treatment is not due to disease progression, death, withdrawal of informed consent or loss to follow-up, the tumor imaging evaluation is still to be performed according to the pre-defined interval, until disease progression, or subsequent anti-cancer treatment.  A independent Data Monitoring Committee (DMC) will be established to regularly perform systematic safety assessment to safeguard the interests and safety of the subjects in the study. The DMC will be responsible for making recommendations to the sponsor to continue or terminate the study based on a periodic assessment of the safety data.  Criteria for the discontinuation of treatment/study:  The treatment may be discontinued at any time if any of the following conditions occur:   - Any treatment-related adverse event (TRAE) CTCAE grade 3 or 4 fails to reverted to CTCAE grade 1 or less within 28 days of treatment interruption (unless the investigator believes that the patient may continue to benefit from continued medication, and it requires permission from the sponsor’s medical monitor) - Any CTCAE grade 3 or 4 TRAE recur despite the fact that the dose has been reduced to the minimum (100mg/d). - Blood platelet count fails to recover to the level of >100,000/µL within 28 days of treatment interruption. - Disease progression according to RECIST v1.1. - Risk to the patient as judged by the investigator or Sponsor. - Poor compliance by the investigator’s or Sponsor’s judgment. - The patient's request - The patients becomes pregnant   Patients who discontinue treatment will continue to receive follow-up assessment (such as CFI, TFST and OS), except for any one of the following conditions:   - The patient withdraws informed consent - Death from any cause - Lost to follow-up - The patient has been unblinded   Unblinding procedure  Subjects, investigators, study coordinators and Sponsor’s study team will be blinded to treatment assignment from randomization to database lock.  Before the primary efficacy endpoint (PFS) results are available in this study, subjects and investigators shall not be unblinded, unless for emergent medical reasons such as treatment-related adverse event (AE)/ serious adverse event (SAE). The study physician of Sponsor may at any time assist the investigator in making the patient unblinded; and once the patient is unblinded, the investigator shall record the cause in the electronic case report form (eCRF) and the unblinded patient shall permanently withdraw from the study.  After the primary analysis results are available in this study, unblinding will be performed for all subjects. For subjects who remained on ZL-2306 (niraparib), based on the investigator’s assessment and discussion with the sponsor, if there is clinical benefit, the patient may continue receiving ZL-2306 (niraparib). For subjects receiving the placebo, based on the investigator’s assessment and discussion with the sponsor, if there is a possibility of clinical benefit from receiving ZL-2306 (niraparib), the patients were allowed to receive ZL-2306 (niraparib) after unblinding.  After unblinding, all patients who continue receiving ZL-2306 (niraparib) will undergo safety monitoring and efficacy assessment according to the medical practices of their respective hospitals until progressive disease or the occurrence of intolerable adverse events or other circumstances requiring treatment discontinuation as specified in the study protocol (see Section 4.5.1 of this protocol). The sponsor has the right to request safety or efficacy data if necessary. | |
| **Planned Number of Subjects:** | No less than 240 |
| **Diagnosis and Main Inclusion Criteria:** | 1. Written informed consent before any study-related procedure. 2. Known g*BRCA* mutation status before randomization. 3. Women aged 18 years or older. 4. Histologically confirmed epithelial ovarian cancer, fallopian tube cancer or primary peritoneal cancer. 5. High-grade (Grade 3) serous or predominantly high-grade serous ovarian cancer (no histological restrictions for patients with ovarian cancer carrying germline BRCA mutations). 6. Having received at least two prior lines of platinum-based chemotherapies, and meet following criteria: 7. After the penultimate line of platinum-based chemotherapy, the patient shall meet the following requirements:  - CR or PR to this line of chemotherapy. - Relapse after this course of chemotherapy must be PSROC, i.e., disease does not progress until 6 months after the course of chemotherapy (on medical records 6-12 months or ≥ 12 months).  1. After the most recent line of platinum-based chemotherapy, the patient shall meet the following requirements:  - Having received at least 4 cycles of platinum-containing chemotherapy (must be carboplatin or cisplatin or nedaplatin). - Clinical response to this line of chemotherapy must be CR (i.e., after this line of chemotherapy, there is no measurable or unmeasurable lesion according to RECIST v1.1, and CA-125 is within the normal range) or PR (after this line of chemotherapy at least 30% reduction in sum of diameters of target lesions compared to the baseline before chemotherapy). - CA-125 level is within the normal range after the chemotherapy, or decreases > 90% during the course of chemotherapy, and remains stable for at least 7 days (increase of CA-125 level before enrollment should not exceed 15% compared with the level after chemotherapy). - No measurable lesion> 2cm (eligibility of lesion > 2cm shall be discussed with the sponsor based on evidence of PR).  1. The patient shall be randomized within 8 weeks after completion of last-line chemotherapy. 2. ECOG performance status 0 or 1. 3. Adequate organ function, including:  - Neutrophil count ≥1,500/µL - Platelet count ≥100,000/µL - Hemoglobin ≥10g/dL - Serum creatinine ≤ 1.5 × ULN, or creatinine clearance rate ≥ 60mL/min (using Cockcroft-Gault equation) - Total bilirubin ≤1.5 × ULN, or direct bilirubin ≤1.0 × ULN. - AST and ALT ≤ 2.5 × ULN, or ≤ 5 × ULN in case of hepatic metastases.  1. Women of childbearing age with a negative pregnancy test at the time of enrollment and having promised to take adequate and effective contraceptive measures or abstinence during the study period and within 3 months after the last dose of study drug. Or women without childbearing potential, defined as below:  - Having received surgical sterilization operation (such as hysterectomy, bilateral ovariectomy, or bilateral tubectomy); or - ≥60 years of age; or - ≥40 and <60 years of age, more than 12 months of menopause, and follicle-stimulating hormone (FSH) are within the post-menopause reference range.  1. Ability to comply with the protocol. 2. The level of any toxicity due to previous chemotherapy has decreased to ≤CTCAE grade 1 or baseline level, except for ≤CTCAE grade 2 stable sensory neuropathy or alopecia. |
| **Exclusion Criteria:** | 1. Known hypersensitivity to active or inactive components of ZL-2306 (niraparib) or drugs with similar chemical structures. 2. Previous treatment with PARP inhibitor. 3. Drainage of Ascites during the last two cycles of the last chemotherapy regimen prior to enrollment. 4. Symptomatic, uncontrollable brain or leptomeningeal metastases. It’s not required to confirm brain metastases by radiologic scan; patients with spinal cord compression who have received definitive treatment and evidence of clinically stable disease for at least 28 days can still be considered for enrollment (in case of controlled central nervous system metastasis, relevant treatment such as radiotherapy or chemotherapy must be given at least 1 month before the study; no new CNS-related symptoms or symptoms indicating disease progression, and either taking a stable dose of steroid, or no steroids). 5. Having received a major surgery 3 weeks before entering the study, or not yet recovered from the surgical effect. 6. Having received a palliative radiotherapy encompassing > 20% of the bone marrow within 1 week prior to enrollment. 7. Invasive cancers other than ovarian cancer within 2 years prior to enrollment (except fully treated basal or squamous cell skin cancer). 8. A previous or current diagnosis of myelodysplastic syndrome (MDS) or acute myeloid leukemia (AML). 9. Other serious or uncontrolled diseases, including but not limited to:  - Uncontrollable nausea and vomiting, inability to swallow study drug, and any gastrointestinal disease that may interfere with the absorption and metabolism of the drug - Active viral infections, such as human immunodeficiency virus, hepatitis B virus, hepatitis C virus and so on - Uncontrolled ventricular arrhythmias and occurrence of myocardial infarction in the past 3 months - Uncontrolled major seizure disorder, unstable spinal cord compression, superior vena cava syndrome or other psychiatric disorders that may prohibits obtaining patients' informed consent - Immunodeficiency (except for splenectomy), or other diseases that investigators believe may expose patients to high-risk toxicity.  1. Any previous or current disease, treatment or laboratory test abnormality that may interfere with the results of the study, affect the patient’s full participation in the study, or the investigator believes that the patient is not suitable for participating in this study; platelet or red blood cell transfusion is not allowed within four weeks prior to initiation of study drug. 2. Pregnancy or breast feeding or planning a pregnancy during the study. |
| **Study Drug and Specification:** | - Formulation and appearance of ZL-2306 (niraparib): pink capsules for oral use - Active ingredients: ZL-2306 (niraparib) Niraparib Tosylate Monohydrate - Indication: anti-cancer drug - Packing: 100mg capsule, packed in the aluminum-plastic blister pack |
| **Evaluation Criteria:**  Efficacy:  Primary endpoint is PFS, defined as the time from randomization to disease progression or death of all causes, whichever occurs first; disease progression will be accessed by an independent central imaging evaluation based on RECIST v1.1.  Secondary endpoints for efficacy evaluation include:  Chemotherapy-free interval (CFI) refers to the time from the most recent platinum-based treatment to the beginning of next anti-cancer treatment (excluding maintenance therapy);  Time to first subsequent treatment (TFST) refers to the time from the date of randomization to the start date of the first subsequent anti-cancer treatment;  Overall survival (OS) refers to the time from the date of randomization to the date of death by any cause.  Safety:  Safety evaluation of ZL-2306 (niraparib) involves AE, clinical laboratory parameters (hematology, serum biochemistry, etc.), vital signs, electrocardiogram, physical examination and concomitant medication, etc. These data will be classified based on type, frequency, severity, correlation with the treatment and laboratory test abnormality, and according to the NCI Common Terminology Criteria for Adverse Events (NCI CTCAE version 4.03, issued in 2010, http://ctep.cancer.gov/reporting/ctc.html)  Adverse events of special interest (AESI) in this study are:   - Myelodysplastic syndrome (MDS) or acute myeloid leukemia (AML) - Secondary cancer (newly diagnosed malignant tumors other than MDS/AML) - Pneumonitis - Embryo-fetal toxicity   The investigator must report serious adverse event (SAE) to the relevant parties within 24 hours of knowledge; report AESI to the sponsor within 24 hours (report directly according to the SAE process, if it meets SAE criteria) | |
| **Statistical Method:**  The primary endpoint of this study is PFS, which will be analyzed once. The overall false positive rate will be controlled at the 2-sided level of 0.05. The study will continue until approximately 155 progression events occur or until 12 months after the last case is randomized, whichever occurs first. For the primary end point PFS, a log-rank test will be used and adjusted according to randomization stratification factors. In addition, a Cox proportional hazard model that takes account of treatment grouping and stratification factors will be used to evaluate the hazard ratio and its 95% confidence interval. For the PFS endpoint, the Kaplan-Meier method will also be used to plot and describe the data.  CFI, TFST and OS will be statistically analyzed using the same method as described above for PFS.  Treatment emergent adverse events (TEAEs) and concomitant medication will be summarized by treatment group. Clinical laboratory indicators, vital signs and ECG data will be summarized by treatment group and study visit. The analysis will describe the observations and changes from the baseline for each visit. | |
| **Schedule and Content of Study Visits:** see [Table 1](#_Hlk486577971) | |

Table 1 – Schedule for Study Visits

| Visits (V) | Screening period | V1 baseline | V2 | V3 | V4 | V5 | V6 | Follow-up cycles ^2^ | End of treatment  (within 7 days after termination) | Follow-up visits after treatment |
| --- | --- | --- | --- | --- | --- | --- | --- | --- | --- | --- |
| Treatment cycle^1^ (28 days/cycle) |  | 1 |  |  |  |  | 2 | 3, 4, 5… |  |  |
| Weeks | -4 to -1 | 1 | 2 | 3 | 3 | 4 | 1 | n^th^ cycle, 1^st^ week |  |  |
| Days | -28 to -1 | 1 | 8 | 15 | 18 | 22 | 1 | n^th^ cycle, 1^st^ day |  |  |
| Visit window (days) |  |  | ±2 | ±2 | ±1 | ±2 | ±5 | ±5 |  |  |
| Informed consent | x |  |  |  |  |  |  |  |  |  |
| Demographic | x |  |  |  |  |  |  |  |  |  |
| Medical, surgical, cancer, treatment history | x |  |  |  |  |  |  |  |  |  |
| Record on whether the patient has received examination of g*BRCA*^3^mutation previously | x |  |  |  |  |  |  |  |  |  |
| Blood collection for examination of g*BRCA*^4^ by central laboratory | x |  |  |  |  |  |  |  |  |  |
| Serum/urine pregnancy test ^5^ | x |  |  |  |  |  |  |  |  |  |
| Randomization ^6^ |  | x |  |  |  |  |  |  |  |  |
| Physical examination | x | x |  | x |  |  | x | x | x |  |
| Vital signs, height,^7^ weight | x | x |  | x |  |  | x | x | x |  |
| ECOG performance status | x | x |  |  |  |  | x | x | x |  |
| AE monitoring ^8^ | x | x |  | x |  |  | x | x | x |  |
| Inquiry about previous treatment and concomitant medications | x | x |  | x |  |  | x | x | x |  |
| Blood coagulation and serum biochemistry | x | x^9^ |  | x |  |  | x | x | x |  |
| CBC^10^ | x | x^9^ | x | x | x^11^ | x | x | x | x |  |
| Serum CA-125^12^ | x | x^9^ |  |  |  |  |  | x | x |  |
| Urinalysis | x |  |  |  |  |  |  |  |  |  |
| 12-lead ECG | x | x^18^ |  |  |  |  | x^18^ |  | x^18^ |  |
| Tumor evaluation (RECIST) ^13^ | x |  |  |  |  |  |  | x | x | x |
| Chest CT/MRI^14^ | x |  |  |  |  |  |  |  |  |  |
| Distribution and re-collection of study drug |  | x |  |  |  |  | x | x | x^15^ |  |
| Evaluation of anti-cancer efficacy |  |  |  |  |  |  |  |  |  | x^16^ |
| Survival evaluation |  |  |  |  |  |  |  |  |  | x^16^ |
| Bone marrow aspiration/biopsy, collection of whole blood samples for diagnosis of MDS/AML |  | x ^17^ | | | | | | | | |

^1^ The treatment cycle will be 28 days, and visit will be made on the first day of each cycle, unless otherwise specified.

^2^ Visits continue every 4 weeks until study treatment discontinuation.

^3^ If a g*BRCA* examination has been performed in the local laboratory previously, the results shall be recorded in the eCRF at screening

^4^ Peripheral blood will be collected and sent to the central laboratory for g*BRCA* mutation detection.

^5^ Female of childbearing age must be confirmed pregnancy test negative within 7 days prior to first dose.

^6^ Randomization must be completed on the day of the first dose or within 72 hours before dosing

^7^ Height will be measured only during the screening.

^8^ SAE recorded until 30 days after discontinuation of study treatment.

^9^ If the screening test is performed within 7 days of the first day of administration, there is no need to repeat the test, including complete blood count (CBC), blood coagulation and serum biochemistry, CA-125

^10^ If treatment discontinuation or dose modification is due to hematological toxicity, weekly CBC shall be performed until the AE is relieved. In order to ensure the safety of the new dose, CBC shall be continued at least once a week when medication is resumed (that is, the re-administration day as the first day, at days1, 8, 15,18,22,29, see table 11) for 4 weeks, and then return to once every 4 weeks. For patients whose initial dose is 200 mg and meet the criteria for dose increment, after starting a new dose, CBC shall also be performed at least once a week as shown in Table 11 (that is, the re-administration day as the first day, at days 1, 8, 15, 18, 22, 29), and then once every 4 weeks. CBC can be performed in local hospitals, other tests and examinations shall be performed at the research center according to the scheduled date.

^11^ If the platelet test result is <100,000 / μL on the 15^th^ day of the first cycle (V3), the patient must stop the study drug, and V4 (on the 18^th^ day of cycle 1) is not necessary, but subsequent visits shall be performed as usual, including D22 of cycle 1 (V5) and all subsequent scheduled visits.

^12^ CA-125 level at the time of screening must be with normal range or reduced by> 90% compared to the level before the most recent line of platinum-based chemotherapy; and abnormal CA-125 does not necessarily represent disease progression. CA-125 testing in conjunction with imaging evaluation shall be performed every 8 weeks (2 treatment cycles) until disease progression; if the disease does not progress by the end of cycle 14, then once every 12 weeks until the disease progression. If the patient's image evaluation after chemotherapy is performed within 28 days before the first dose, it may not be repeated during the screening period.

^13^ CT or MRI scans of the abdominal/pelvic cavity and clinically indicated sites shall be performed and evaluated according to the RECIST once every 8 weeks (2 cycles) until disease progression; if no progression by the end of the 14^th^ cycle, then once every 12 weeks until the disease progression. All images shall be assessed by blinded independent central review.

^14^ Chest CT/MRI (If the site is not included in the baseline tumor RECIST assessment, a baseline assessment will be required; if no tumor is detected in baseline chest CT/MRI, it is not necessary for subsequent routine evaluation on this site in the absence of clinical signs; if a tumor is found at baseline, then chest scan shall be included in subsequent visits). If the patient's image evaluation after chemotherapy is performed within 28 days before the first dose, it may not be repeated during the screening period

^15^ No new drug dispensed.

^16^ Once every three months after the treatment discontinuation.

^17^ Bone marrow aspiration/biopsy shall be performed by local hematologists for all MDS/AML cases.

^18^ 12-lead ECG shall be performed within 72 hours before randomized; a repeat ECG is not necessary before day 1 of the 1^st^ cycle in the absence of symptoms.

**Table of Contents**

[1. Introduction 17](#_Toc152238061)

[1.1 Background 17](#_Toc152238062)

[1.1.1 Ovarian Cancer 17](#_Toc152238063)

[1.1.2 BRCA mutation, PARP and PARP inhibitor ZL-2306 (Niraparib) 18](#_Toc152238064)

[1.1.3 Pre-clinical Research of Niraparib 19](#_Toc152238065)

[1.1.4 Clinical Research of Niraparib 22](#_Toc152238066)

[1.2 Research Principle and Risk/Benefit Evaluation 27](#_Toc152238067)

[2 Research Objectives 28](#_Toc152238068)

[2.1 Primary Objective 28](#_Toc152238069)

[2.2 Secondary Objective 28](#_Toc152238070)

[3 Research Plan 29](#_Toc152238071)

[3.1 Design of Research 29](#_Toc152238072)

[3.2 Discussion on Design of Research 32](#_Toc152238073)

[3.3 End of study 33](#_Toc152238074)

[4 Population of Research 34](#_Toc152238075)

[4.1 Inclusion Criteria 34](#_Toc152238076)

[4.2 Exclusion Criteria 35](#_Toc152238077)

[4.3 Previous and Concomitant Medication 37](#_Toc152238078)

[4.3.1 Restrictions on Concomitant Treatment 37](#_Toc152238079)

[4.4 Handling of Subjects Enrolled Incorrectly 38](#_Toc152238080)

[4.5 Discontinuation of Medication 39](#_Toc152238081)

[4.5.1 Termination of Treatment 39](#_Toc152238082)

[4.5.2 Withdrawal from Study 39](#_Toc152238083)

[4.6 Identification Number of Subjects and Randomized Assignment 41](#_Toc152238084)

[4.6.1 Identification Number of Subjects 41](#_Toc152238085)

[4.6.2 Randomization Scheme 41](#_Toc152238086)

[4.7 Planned Numbers of Patients and Research centers 41](#_Toc152238087)

[5 Study Treatment 42](#_Toc152238088)

[5.1 Study Treatment Regime 42](#_Toc152238089)

[5.2 Properties of Study Drugs 43](#_Toc152238090)

[5.3 Package and Label 43](#_Toc152238091)

[5.4 Treatment Compliance 43](#_Toc152238092)

[5.5 Study drug reception, storage and management responsibilities 44](#_Toc152238093)

[5.6 Access to Study Drug upon Completion of Trial 44](#_Toc152238094)

[6 Visit Schedule and Evaluation 44](#_Toc152238095)

[6.1 Evaluation of Study Visit 44](#_Toc152238096)

[6.1.1 Screening period (visit 1, from day -28 to day -1) 45](#_Toc152238097)

[6.1.2 First Cycle 47](#_Toc152238098)

[6.1.3 Day 1 of the second cycle (C2D1) 48](#_Toc152238099)

[6.1.4 Day 1 of subsequent cycles in order 48](#_Toc152238100)

[6.2 Termination of Study Treatment (Visit will be made within 7 days after the last dose of study drug) 48](#_Toc152238101)

[6.3 Assessment after Completion of Study Treatment (subjects are not required to return to the research center to complete the visit) 49](#_Toc152238102)

[6.4 Unplanned Follow-up Visit 49](#_Toc152238103)

[7 Efficacy & Safety Assessment and Exploratory Research 49](#_Toc152238104)

[7.1 Efficacy Assessment 49](#_Toc152238105)

[7.1.1 Primary Study Endpoint: PFS 51](#_Toc152238106)

[7.1.2 Secondary Study Endpoints 52](#_Toc152238107)

[7.2 Safety Evaluation 53](#_Toc152238108)

[7.2.1 Laboratory Tests 53](#_Toc152238109)

[7.2.2 Electrocardiogram Evaluation 54](#_Toc152238110)

[7.2.3 Physical Examination 55](#_Toc152238111)

[7.2.4 Vital Signs 55](#_Toc152238112)

[7.2.5 Eastern Collaborative Oncology Group (ECOG) Performance Status 55](#_Toc152238113)

[8 Safety Monitoring, Reporting and Medical Treatment 55](#_Toc152238114)

[8.1 Adverse Event (AE) – Definition 55](#_Toc152238115)

[8.2 Serious Adverse Event (SAE) – Definition 55](#_Toc152238116)

[8.3 Collection, Recording, Follow-up Visit and Reporting of Adverse Events 56](#_Toc152238117)

[8.3.1 Time Limit for Collection of Adverse Events 56](#_Toc152238118)

[8.3.2 Follow-up of Unresolved AE 56](#_Toc152238119)

[8.3.3 Elements for Collection of AE 56](#_Toc152238120)

[8.3.4 Collection of Causal Relationship between Study Drug and AE 57](#_Toc152238121)

[8.3.5 Examination- and Test-Based AE 58](#_Toc152238122)

[8.3.6 Progression of Disease 58](#_Toc152238123)

[8.3.7 Management of Death Case 58](#_Toc152238124)

[8.3.8 Adverse Event of Special Interest (AESI) 59](#_Toc152238125)

[8.3.9 Reporting of SAE 59](#_Toc152238126)

[8.3.10 Pregnancy 59](#_Toc152238127)

[8.3.11 Overdose 60](#_Toc152238128)

[8.4 Management of Toxicity Related to the Study Drug 60](#_Toc152238129)

[8.4.1 Management of Non - hematological toxicity 60](#_Toc152238130)

[8.4.2 Management of Hematologic Toxicity 61](#_Toc152238131)

[8.5 Data Monitoring Committee (DMC) 65](#_Toc152238132)

[9 Statistic Analysis 65](#_Toc152238133)

[9.1 Analytical Data Set 66](#_Toc152238134)

[9.2 Demographics, Medical History, Baseline Characteristics and Combined Medication 66](#_Toc152238135)

[9.3 Efficacy Analysis 66](#_Toc152238136)

[9.3.1 Primary Efficacy Endpoint 66](#_Toc152238137)

[9.3.2 Secondary and Exploratory Efficacy Endpoint 67](#_Toc152238138)

[9.4 Safety Analysis 67](#_Toc152238139)

[9.5Sample Size 67](#_Toc152238140)

[10 Management of Clinical Trial 68](#_Toc152238141)

[10.1 Declaration 68](#_Toc152238142)

[10.2 Ethics 68](#_Toc152238143)

[10.3 Review of Original Records 69](#_Toc152238144)

[10.4 Quality Assurance and Audit 69](#_Toc152238145)

[10.5 Informed Consent Form (ICF) 69](#_Toc152238146)

[10.6 Modification to Clinical Research Protocol 69](#_Toc152238147)

[10.7 Case Report Form (CRF) 70](#_Toc152238148)

[10.8 Inspection 70](#_Toc152238149)

[10.9 Confidentiality Agreement and Privacy of Patients 70](#_Toc152238150)

[11 Publication of Papers 71](#_Toc152238151)

[12 Archiving of Materials 71](#_Toc152238152)

[13 Reference 71](#_Toc152238153)

[14 Annexes 73](#_Toc152238154)

[Annex 1 Drugs Considered as a Sensitive Substrate of CYP1A2 73](#_Toc152238155)

[Annex 2 ECOG Scale of Performance Status 73](#_Toc152238156)

[Annex 3 User Manual for Response Evaluation Criteria in Solid Tumors Version 1.1 (RECIST 1.1) 73](#_Toc152238157)

[1. Overview of evaluation process 73](#_Toc152238158)

[2. Measurement of tumor at baseline 73](#_Toc152238159)

[2.1 Measurement of Lesions 73](#_Toc152238160)

[3. Tumor response evaluation 76](#_Toc152238161)

[3.1 Assessment of overall tumor burden and measurable disease 76](#_Toc152238162)

[3.2 Tumor Response criteria 77](#_Toc152238163)

[3.2.1 Evaluation of Target Lesions 77](#_Toc152238164)

[3.2.2 Special Considerations for Evaluation of Target Lesions 77](#_Toc152238165)

[3.2.3 Evaluation of non-target lesions 79](#_Toc152238166)

[3.2.4 Special notes on assessment of progression of non-target lesions disease 79](#_Toc152238167)

[3.2.5 Tumor imaging evaluation for patients without any lesion (either measurable or non-measurable) at baseline 80](#_Toc152238168)

[3.2.6 New lesions 80](#_Toc152238169)

[3.3 Considerations during response assessment 81](#_Toc152238170)

[3.4 Missing assessments and inevaluable confirmation 82](#_Toc152238171)

[3.5 Assessment of tumor remission status at specific time points 82](#_Toc152238172)

**Directory of Tables**

[Table 1 – Schedule for Study Visits 8](#_Toc152237227)

[Table 2 – Summary of TEAEs in NOVA Study 24](#_Toc152237228)

[Table 3 – Summary of Common AEs (all grades) and CTCAE Grade-3/4 AEs in NOVA study 24](#_Toc152237229)

[Table 4 – Common AEs Effectively Controlled Through Dose Adjustment in NOVA Study 25](#_Toc152237230)

[Table 5 - PFS of Ovarian Cancer Patients in NOVA Study Based on Independent Center Review (ITT Population) 27](#_Toc152237231)

[Table 6 – Interpretation of gBRCA Test Results and Randomization Stratification Basis 30](#_Toc152237232)

[Table 7 – Dose Adjustment Scheme for ZL-2306 (Niraparib) 43](#_Toc152237233)

[Table 8 – Drugs for Study 43](#_Toc152237234)

[Table 9 – Laboratory Tests 54](#_Toc152237235)

[Table 10 – Dose Modification due to Non-hematologic Toxicity 61](#_Toc152237236)

[Table 11 – Schedule for Safety Monitoring after Medication is Restored from a Suspension due to Hematologic Toxicity 65](#_Toc152237237)

[Table 12 – Censoring Rules for Analysis of Primary Endpoint (PFS) 67](#_Toc152237238)

**Abbreviations and Terms**

| **Abbreviations** | **Full Form** |
| --- | --- |
| ADP | Adenosine diphosphate |
| AE | Adverse event |
| ALT | Alanine aminotransferase |
| AML | Acute myeloid leukemia |
| ANC | Absolute neutrophil count |
| APTT | Activated partial thromboplastin time |
| AST | Aspartate transaminase |
| ATC | Anatomical Therapeutic Chemical Classification System |
| ATM | Ataxia telangiectasia mutated |
| AUC | Area under the curve |
| BCRP | Breast cancer resistance protein |
| BER | Base excision repair |
| BRCA | Breast cancer susceptibility gene |
| CBC | Complete blood count |
| CFI | Chemotherapy-free interval |
| CI | Confidence interval |
| C_max_ | Maximum concentration |
| CR | Complete response |
| CRF | Case report form |
| CT | Computer tomography |
| CTCAE | Common terminology criteria for adverse events |
| ECG | Electrocardiogram |
| ECOG | US Eastern Collaborative Oncology Group |
| eCRF | Electronic case report form |
| EMA | European Medicines Agency |
| ESMO | European Society of Medical Oncology |
| FDA | Food and Drug Administration |
| g*BRCA*mut | Germline BRCA mutation |
| GCP | Good Clinical Practice |
| GGT | Glutamyl transpeptidase |
| HDPE | High-density polyethylene |
| HIV | Human immunodeficiency virus |
| HR | Hazard ratio |
| HRD | Homologous recombination defects |
| ICF | Informed consent form |
| DMC | Data monitoring committee |
| EC | Ethics committee |
| ICH | International Conference on Harmonization |
| INR | International normalized ratio for prothrombin time |
| ITT | Intent-to-treat population |
| IWRS | Interactive Web Response System |
| MCH | Mean corpuscular hemoglobin |
| MCV | Mean corpuscular volume |
| MDS | Myelodysplastic syndrome |
| NHEJ | Non-homologous end joining |
| MedDRA | Medical Dictionary for Regulatory Activities |
| MRI | Magnetic resonance imaging |
| NCCN | National Comprehensive Cancer Network |
| NCI | National Cancer Institute |
| OCT1 | Organic cation transporter 1 |
| OS | Overall survival |
| PARP | Poly(ADP-ribose) polymerases |
| PD | Progression of disease |
| PFS | Progression-free survival |
| P-gp | P-glycoprotein |
| PP | Per-protocol population |
| PR | Partial response |
| QD | Once a day |
| RECIST | Response Evaluation Criteria in Solid Tumors |
| SAE | Severe adverse events |
| SAP | Statistical analysis plan |
| SD | Stable disease |
| SUSAR | Suspected unexpected serious adverse reaction |
| t_1/ 2_ | Half life |
| TEAE | Treatment emergent adverse event |
| T_max_ | Time to peak |
| TMZ | Temozolomide |
| ULN | Upper limit of normal |
| Vd/F | Apparent volume of distribution |
| WHO | World Health Organization |

# 1. Introduction

## 1.1 Background

### 1.1.1 Ovarian Cancer

Ovarian cancer (OC) is one of the most common malignant tumors of the female genital organs and is also one of the major causes of death of gynecological malignancies in China. In 2011, 45,223 new cases of ovarian cancer were diagnosed in China, and 18,430 deaths were caused by ovarian cancer^1^.

Early stage of ovarian cancer is usually asymptomatic, and about 75% OC patients have advanced disease at the time of diagnosis. The standard therapies of ovarian cancer mainly include surgical resection (cytoreductive surgery or debulking) and combination chemotherapy of paclitaxel and platinum. However, most patients will encounter following problems within 2 years after the above therapies, including recurrence of disease, drug resistance to platinum-containing therapy, decrease of the toxicity tolerance to the chemotherapy drugs, reduction of progression-free survival period, decrease of subsequent effective treatment options, and gradual worsening of prognosis. Patients with distant metastasis of ovarian cancer have a very low 5-year survival rate (only 29%)^2^.

The treatment of recurrent ovarian cancer relies on the interval between the last medication date of the previous line of treatment and the recurrence date. Platinum-sensitive recurrent ovarian cancer (PSROC) is a kind of recurrent cancer, tumor progression occurs ≥6 months after the last dose of prior platinum-based chemotherapy^3, 4^. The National Comprehensive Cancer Network (NCCN) and the European Society of Medical Oncology (ESMO) guidelines recommend platinum-based chemotherapy for these patients.

Though there are limited effective treatment options for PSROC, disease relapse is still inevitable. The goal of treatment for such patients is to prolong disease control and delay the inevitable disease progression. However, prolonging the treatment duration of chemotherapy will lead to the accumulation of toxicity with limited additional effect, so it is only a choice after disease progression. Although bevacizumab is approved as a treatment option in the EU, its efficacy is limited, and it must be used in combination with chemotherapy, accompanied by increased toxicity. Furthermore, if bevacizumab is used as a first-line drug, it could no longer be used for subsequent treatment. Olaparib has been approved as a maintenance option in the EU, but its target population is small, limited to patients with breast cancer susceptibility gene (*BRCA*) mutations.

Bevacizumab has not been approved for ovarian cancer in China; it is expensive and requires expertise to administrate the drug and to monitor its safety, therefore bevacizumab is rarely used in ovarian cancer clinical practice. In addition, since no PARP inhibitor has been approved in China, there is a complete lack of effective subsequent option after 2^nd^ line of treatment for patients with PSROC, so they have to wait for the inevitable disease recurrence. In China, there is an urgent need for new drugs that can prolong disease control and delay the recurrence of ovarian cancer.

### 1.1.2 BRCA mutation, PARP and PARP inhibitor ZL-2306 (Niraparib)

*BRCA* gene is a tumor suppressor gene, which plays an important role in DNA damage repair and normal cell growth, etc. Significant mutations in this gene can inhibit the ability of DNA to repair correctly after damage, such as causing homologous recombination DNA double-strand repair deficiency (HRD)—the loss of BRCA function makes the repair of double-strand DNA breaks impossible through homologous recombination DNA repair pathway, and the damaged cells have to use the low-fidelity compensatory backup alternative DNA repair pathways such as non-homologous end joining (NHEJ) to complete the repair. The low-fidelity compensatory alternative DNA repair may lead to accumulation of cell mutations, and eventually result in the occurrence of ovarian cancer, breast cancer and other malignancies. Complete loss of function or complete inactivation of the two alleles of BRCA is common in g*BRCA* mutant female carriers, and patients with this gene mutation have a significantly increased risk of ovarian cancer or breast cancer, of which the risk of ovarian cancer is 10-30 times^5^ higher than that of individuals with wild-type *BRCA*.

Poly(ADP-ribose) polymerases (PARP-1 and PARP-2) are zinc-finger DNA binding enzymes whose function is to detect DNA single-strand damage and promote repair. PARPs detect DNA damage and convert it into intracellular signals, which then activate the base excision repair (BER) pathway^6^. This pathway is particularly important for the cells with defect in the high-fidelity double-strand DNA repair mechanism (HRD). As mentioned above, inactivation of some genes such as *BRCA*1/2 gene and ataxia telangiectasia mutated (*ATM)* gene can lead to HRD. In the context of a defect in DNA double-strand repair, cell DNA damage repair will depend more on single-strand damage repair pathways; therefore, when PARP inhibitor is used in ovarian cancer patients with extensive existence of HRD caused by *BRCA* gene abnormality and other reasons, PARP inhibitor may cause the blockage and inhibition in DNA single-strand damage repair, which, combined with DNA double-strand repair defect caused by HRD and other reasons, may result in a synergistic and enhanced induction of cell death^7^; meanwhile, PARPi itself has the cell death induction mechanism, implying that application of such an inhibitor may also cause the anti-tumor effect in the ovarian cancer patients with non-*BRCA* abnormalities. Pre-clinical in vitro and in vivo studies have confirmed the availability of the target. Therefore, therapy targeting PARP has a solid scientific basis in ovarian cancer patients.

In recent years, a number of clinical studies have shown that PARP inhibitor is effective in treatment of recurrent ovarian cancer^8, 9, 10^. Niraparib is a potent and highly selective PARP-1/2 inhibitor developed by TESARO, undergoing the clinical development phase in the world (except China). In a recent phase III, randomized, placebo-controlled, international multicenter clinical trial, Niraparib as maintenance treatment significantly prolonged the PFS of patients with PSROC. This is by far the first completed Phase III confirmatory study that has confirmed the clinical efficacy of the PARP inhibitor for recurrent ovarian cancer; more importantly, Niraparib has showed significant efficacy in ovarian cancer patients both with and without gBRCA mutation. Based on the strong clinical supportive data from this phase III clinical trial, in September and December of 2016, the US FDA granted the application fast track designation and priority review, respectively; and in March of 2017, the drug was rapidly approved by FDA for marketing 3 months in advance as maintenance treatment for platinum-sensitive recurrent ovarian cancer. In September 2016, Zai Lab obtained the exclusive right to research, develop, produce and distribute Niraparib in China, and numbered it as ZL-2306.

### 1.1.3 Pre-clinical Research of Niraparib

#### 1.1.3.1 Pharmacodynamics

In vitro enzymology experiments have shown, IC50s of Niraparib for inhibiting PARP-1 and PARP-2 are 3.8 nM and 2.1 nM, respectively. Compared with other PARP family members, it exhibited at least a 100-fold higher selectivity. In vitro cell experiments have confirmed that Niraparib can functionally inhibit poly (ADP) ribosylation and repair of single-strand DNA breaks, and IC50 and IC90 in Jurkat cells are 22 nM and 56 nM, respectively. In addition, Niraparib can selectively inhibit the growth of *BRCA*1/2-deficient tumor cell lines. For *BRCA1*-deficient HeLa cells, the concentration of Niraparib required to reduce the cell growth rate by 50% is 34 nM, much lower than that for *BRCA*1wt HeLa cells (852 nM).

In vivo studies using xenograft models have shown that Niraparib has antitumor activity in both the *BRCA1* mutant ovarian cancer cell line and those derived from untreated high-grade serous ovarian cancer patients.

#### 1.1.3.2 Pharmacokinetics

**Absorption:** Niraparib p-toluenesulfonate monohydrate has good permeability; after oral use, it can be absorbed rapidly in rats and canines, and the time to peak (T_max_) is 2 hours and 0.5 hours respectively; but in rhesus monkeys, the absorption is slower, and T_max_ is 4 to 8 hours.

**Distribution:** In rats and dogs, the apparent distribution volume is relatively large, 6.9 and 12.3 L/kg respectively, indicating that Niraparib can be widely distributed into various tissues of rats and canines, which is consistent with the high apparent volume of distribution (Vd/F: 1220 and 2170 L/kg) measured in human body in clinical trials, suggesting that it may have a better effect on solid tumors. Niraparib has a relatively high dissociation ratio in plasma of various genera and species, with a protein binding ratio is of 71.6~84.3%, and is less concentration dependent, indicating that a higher effective blood drug concentration can be achieved at a lower dose, while the risk of toxicity caused by the increase of free drug concentration due to the competitive protein binding is relatively small. Niraparib can penetrate the blood-brain barrier, with the AUC0-24h ratios in rat brain tissue and plasma being 0.85 (10 mg/kg) and 0.88 (30 mg/kg) respectively; and in cerebrospinal fluid and plasma of rhesus monkeys, the AUC_(0-inf)_ and C_max_ are 19% and 31% respectively, suggesting that Niraparib has a potential therapeutic effect on brain metastases.

**Metabolism:** Niraparib is very stable in human liver microsomes and hepatocytes, producing only traces of amide hydrolysis and oxidation products. The amount of all metabolites produced by metabolism in rats is less than 5% of the amount administered, and the main metabolites are acid hydrolysates of amides and their glucuronic acid conjugates. In both in vitro canine hepatic cell and the in vivo experiments, acid hydrolysates of amides are the predominant metabolites (~50%). Studies have shown that carboxylesterase is the main metabolic enzyme that promotes the production of acid hydrolysates of amides; and CYP1A1/2 and CYP3A4/5 are the major metabolic enzymes that promote the production of oxidative metabolites, whereas CYP2D6 is a secondary metabolic enzyme. Niraparib is a substrate for P-glycoprotein (P-gp) and breast cancer resistance protein (BCRP), nonetheless, it shows a high permeability, indicating that the efflux effect of P-gp and BCRP on Niraparib is limited, therefore, P-gp and BCRP will not affect the absorption and distribution of Niraparib. Niraparib is an inhibitor for BCRP, with a half-inhibitory concentration (IC50) of 5.8 μM; Niraparib is a weak inhibitor for organic cation transporter 1 (OCT1) with an IC50 of 34.1 μM; and Niraparib also slightly inhibits P-gp, with an IC50 of 131-191 μM. Since the plasma C_max_ of free Niraparib at the clinically effective dose is only 0.68μM, slightly greater than 10% (11.7%) IC50 of Niraparib for BCRP transporter, the possibility of causing clinical inhibition is relatively low. Similarly, the value is far less than 10% IC50 of Niraparib for OCT1 (1.99%) and P-gp (<0.52%). Therefore according to the CFDA guidelines, no further research on the inhibition of OCT1 and P-gp are required at the clinical stage. Niraparib does not interact with other major CYP metabolic enzymes, as well as liver and kidney efflux and uptake transporters, so Niraparib is unlikely to trigger drug interaction clinically.

**Excretion:** In rats, Niraparib is mainly excreted as original drug (~45%), and its major metabolites include M1 (amide hydrolyzate metabolite), M10 (glucuronic acid conjugate of M1) and other oxidation products; the amount of all metabolites produced is less than 5% of the amount administered. The excretion route is bile, urine and feces, each accounting for 1/3. In canines, Niraparib is mainly excreted as M1 (~ 52%), through urine (~ 53%), bile (18%) and feces (9%) routes.

#### 1.1.3.3 Toxicology

Overseas toxicology research projects completed include oral repeated-dose toxicity studies, genotoxicity studies and phototoxicity studies in dogs and rats. Niraparib is a potent and highly selective PARP-1 and PARP-2 inhibitor, and given that expression of PARP-1 and PARP-2 genes is essential for early embryonic development, it is expected to have embryo-fetal toxicity. Repeated-dose toxicity studies have also confirmed that Niraparib p-toluenesulfonate monohydrate leads to a reduction in the number of spermatogenic epithelium in dogs and the number of testicular sperm cells in rats. It is foreseeable that Niraparib p-toluenesulfonate monohydrate has reproductive and developmental toxicity. Additionally, Niraparib has gained the positive result in the genetic toxicology study completed. Therefore, according to the technical guidelines for preclinical research of antitumor drugs, no research on reproductive and developmental toxicity has been carried out.

Repeated-dose toxicity studies in rats and dogs demonstrated compound-related changes were dose-dependent, consistent and repeatable among animal species, and all observed changes were reversible. Bone marrow was the main target organ of Niraparib. Bone marrow suppression could reduce the amount of white blood cells and change the number of platelets, and even lead to death and early execution. In a 7-day repeated-dose toxicity study in rats, the blood platelet count showed only a brief reduction. In the 30-day and 90-day repeated-dose studies in the rats, the blood platelet count showed an increasing trend, indicating that the animals themselves adjusted for platelet production, and platelet count increased significantly during the recovery period. The platelet count in the dogs was relatively stable, and the increase in platelet count was not observed until the recovery period after the end of administration. Bone marrow suppression and loss of lymphocytes led to infection and sepsis.

The results of Ames showed that Niraparib has no mutagenic effect: in the in vitro chromosomal aberration assay, Niraparib induced the concentration-dependent chromosome structural aberration, but did not cause aberrations in the number of chromosomes. Bone marrow micronucleus induction test results of oral gavage were positive. This result is consistent with the pharmacological activity of Niraparib in inhibition of DNA repair.

In summary, Niraparib has clear action target and mechanism, significant in vivo anti-tumor effect and clear pharmacodynamic-pharmacokinetic relationship; it has good in vivo and in vitro pharmacokinetic properties. It does not cause serious toxic and side effects in or outside the body. The toxic reactions are reversible, measurable and controllable.

A detailed description on the pre-clinical study of Niraparib can be found in the Investigator's Brochure (IB).

### 1.1.4 Clinical Research of Niraparib

#### 1.1.4.1 Clinical Trials Completed and Ongoing

The Table below shows the clinical trials of Niraparib that have completed or are Ongoing:

| **Phase I** | |
| --- | --- |
| PN001 | Phase I clinical study on Niraparib in patients with advanced solid tumors or hematological malignancies (completed) |
| PN008 | Phase I clinical study on treatment with combination of Niraparib and carboplatin in patients with advanced solid tumors (terminated due to non-safety reasons) |
| PN011 | Phase I clinical study on treatment with combination of Niraparib and adriamycin in patients with advanced solid tumors (terminated due to non-safety reasons) |
| PN014 | Phase I clinical study on treatment with combination of Niraparib and temozolomide in patients with advanced solid tumors (completed) |
| PR-30-5015-C | Phase I clinical study on absorption, metabolism and excretion of Niraparib in patients with advanced solid tumors (ongoing) |
| **Phase I/II and Phase II** | |
| TOPACIO | Phase I/II clinical study on treatment with combination of Niraparib and Pembrolizumab in patients with breast cancer or ovarian cancer (ongoing) |
| QUADRA | Phase II, single-arm, open-label clinical trial on Niraparib in recurrent ovarian cancer patients who have received at least 3 lines of treatment (ongoing) |
| 64091742PC  R2001  (Janssen) | Phase II, single-arm, open-label clinical trial on Niraparib in patients with castration resistant prostate cancer (CRPC) (ongoing) |
| **Phase III** | |
| NOVA | Phase III, randomized, double-blind study to evaluate the efficacy and safety of Niraparib versus placebo as maintenance treatment of patients with platinum-sensitive recurrent ovarian cancer (completed) |
| NOVA_QT_C_ study | NOVA sub-study to evaluate effect of Niraparib treatment dosage on QTc (completed) |
| NOVA_FE study | NOVA sub-study to evaluate effect of food on pharmacokinetics of Niraparib (completed) |
| BRAVO | Phase III, open-label, randomized, controlled study to evaluate the efficacy and safety of Niraparib versus physician-selected monotherapy in ovarian cancer patients with Her2- negative and gBRCA mutation (ongoing) |
| PRIMA | Phase III, randomized, double-blind study to evaluate the efficacy and safety of Niraparib versus placebo in first-line maintenance treatment of patients with stage III/IV ovarian cancer (ongoing) |

A detailed description on the clinical study of Niraparib can be found in the Investigator's Brochure (IB).

#### 1.1.4.2 Clinical Safety Data

As of August of 2016, there were 6 phase I, 1 phase I/II, 2 phase II and 3 phase III (including 2 sub-studies) clinical trials of Niraparib around the world, and totally 854 patients received Niraparib treatment, including 751 cases of ovarian cancer, detailed as below:

- 367 cases from NOVA studies
- 50 cases from PN001 study
- 291 cases from QUADRA study
- 26 cases from NOVA QTc study
- 17 cases from NOVA FE study

In the PN001 Phase I clinical study, 104 tumor patients received Niraparib in the dose range of 30 mg to 400 mg; the maximum tolerated dose (MTD) of Niraparib is 300 mg QD; dose-limiting toxicity (DLT): CTCAE grade-4 thrombocytopenia in 2 of 6 patients at 400 mg dose level, CTCAE grade-3 fatigue in 1 patient at 30 mg dose level, and CTCAE Grade-3 pneumonitis in 1 patient at 60 mg dose level.

Common AEs (> 20%) in this study include nausea (56.7%), anemia (49.0%), vomiting (39.4%), constipation (38.5%), thrombocytopenia (34.6%), anorexia (33.7%), headache (25.0%), neutropenia (24.0%), hyponatremia (23.1%), cough (22.1%), dyspnea (21.2%) and diarrhea (21.2%).

In the NOVA study, 367 and 179 ovarian cancer patients were treated with Niraparib or placebo at an initial dose of 300 mg. Treatment emergent adverse events (TEAE) in this study are summarized in [**Table 2**](#_Hlk486521225). Common AEs (all grades) and CTCAE grade-3/4 AEs are summarized in [**Table 3**](#表3CTC). Most non-hematologic adverse events were mild to moderate; although CTCAE grade-3/4 abnormalities in hematology laboratory test results were common at the beginning of the Niraparib treatment, there were no serious clinical consequence (such as bleeding etc.), and the proportion of treatment discontinuation due to these AEs was very low. After the first three cycles of dose modification, the incidence and severity of the above-mentioned adverse events were significantly reduced, indicating that the dose adjustment regimen can effectively manage the adverse effects of the drug (seeing [**Table 4**](#表4)).

Table 2 – Summary of TEAEs in NOVA Study

| **Reported by patients** | **Niraparib**  **(N =367)** | **Placebo**  **(N=179)** |
| --- | --- | --- |
| Any TEAE | 367 (100.0) | 171 (95.5) |
| Any TEAE related to the treatment | 358 (97.5) | 127 (70.9) |
| Any CTCAE grade-3/4 TEAE | 272 (74.1) | 41 (22.9) |
| Any CTCAE grade-3/4 TEAE related to the treatment | 237 (64.6) | 8 (4.5) |
| Any serious TEAE | 110 (30.0) | 27 (15.1) |
| Any serious TEAE related to the treatment | 62 (16.9) | 2 (1.1) |
| Any TEAE leading to treatment interruption | 253 (68.9) | 9 (5.0) |
| Any TEAE leading to dose reduction | 244 (66.5) | 26 (14.5) |
| Any TEAE leading to treatment discontinuation | 54 (14.7) | 4 (2.2) |
| Any TEAE leading to death | 0 | 0 |

Table 3 – Summary of Common AEs (all grades) and CTCAE Grade-3/4 AEs in NOVA study

| **Events, no (%)** | **All grades** | | **Grade 3/4** | |
| --- | --- | --- | --- | --- |
|  | **Niraparib**  **(n=367)** | **Placebo**  **(n=179)** | **Niraparib**  **(n=367)** | **Placebo**  **(n=179)** |
| Nausea | 270(73.6) | 63(35.2) | 11(3.0) | 2(1.1) |
| Thrombocytopenia | 225(61.3) | 10(5.6) | 124(33.8) | 1(0.6) |
| Fatigue | 218(59.4) | 74(41.3) | 30(8.2) | 1(0.6) |
| Anemia | 184(50.1) | 12(6.7) | 93(25.3) | 0 |
| Constipation | 146(39.8) | 36(20.1) | 2(0.5) | 1(0.6) |
| Vomiting | 126(34.3) | 29(16.2) | 7(1.9) | 1(0.6) |
| Neutropenia | 111(30.2) | 11(6.1) | 72(19.6) | 3(1.7) |
| Headache | 95(25.9) | 17(9.5) | 1(0.3) | 0 |
| Decreased appetite | 93(25.3) | 26(14.5) | 1(0.3) | 1(0.6) |
| Insomnia | 89(24.3) | 13(7.3) | 1(0.3) | 0 |
| Abdominal pain | 83(22.6) | 53(29.6) | 4(1.1) | 3(1.7) |
| Dyspnea | 71(19.3) | 8(4.5) | 4(1.1) | 2(1.1) |
| Hypertension | 71(19.3) | 8(4.5) | 30(8.2) | 4(2.2) |
| Diarrhea | 70(19.1) | 37(20.7) | 1(0.3) | 2(1.1) |
| Dizziness | 61(16.6) | 13(7.3) | 0 | 0 |
| Cough | 55(15.0) | 8(4.5) | 0 | 0 |
| Back pain | 49(13.4) | 21(11.7) | 2(0.5) | 0 |
| Arthralgia | 43(11.7) | 22(12.3) | 1(0.3) | 0 |
| Dyspepsia | 42(11.4) | 17(9.5) | 0 | 0 |
| Nasopharyngitis | 41(11.2) | 13(7.3) | 0 | 0 |
| Urinary tract infection | 38(10.4) | 11(6.1) | 3(0.8) | 2(1.1) |
| Palpitations | 38(10.4) | 3(1.7) | 0 | 0 |
| Dysgeusia | 37(10.1) | 7(3.9) | 0 | 0 |
| Myalgia | 30(8.2) | 18(10.1) | 1(0.3) | 0 |
| Abdominal distention | 28(7.6) | 22(12.3) | 0 | 1(0.6) |

Table 4 – Common AEs Effectively Controlled Through Dose Adjustment in NOVA Study

| **Events/no (%)** | **Any grade**  **Leading to reduction of dose (N=367)** | **Grade 3/4**  **Incidence of event after the third cycle (N=296)** | **Any grade**  **Leading to discontinuation of treatment (N=367)** |
| --- | --- | --- | --- |
| Thrombocytopenia | 148(40.3) | 7(2.4) | 12(3.3) |
| Anemia | 68(18.5) | 50(16.9) | 5(1.4) |
| Neutropenia | 32(8.7) | 8(2.7) | 7(1.9) |
| Fatigue | 20(5.4) | 9(3.0) | 12(3.3) |
| Hypertension | 5(1.4) | - | 1(0.2) |

The major risks known in the Niraparib clinical development program include myelosuppression (thrombocytopenia, anemia, neutropenia, various types of hemocytopenia) and hypertension; and the potential major risk of Niraparib is MDS/AML, and according to the NOVA study its incidence was not significantly different between the Niraparib group (5 of 367 patients, 1.4%) and the placebo arm (2 of 179 patients, 1.1%).

No deaths were reported during the treatment in the NOVA study. During the follow-up period after treatment, 3 deaths caused by MDS/AML were reported, including 1 patient treated with Niraparib and 2 patients treated with placebo. Two of the deaths were assessed as treatment-related. One patient in the Niraparib group died eight months after the last dose of Niraparib, and one patient in the placebo group died about one year after the discontinuation of placebo. Of the 384 ovarian cancer patients treated with niraparib in other open-label studies, five TEAEs that end with death were reported, including:

- Four in the QUADRA study - Two were treatment-related: one was due to gastrointestinal hemorrhage, The duration of Niraparib treatment was 12 days, with existence of confounding factors (long-term use of bevacizumab, history of myelosuppression, active gastritis/gastric ulcer and disease progression); the other was due to acute respiratory distress syndrome, related to disease progression or Niraparib. The remaining two were septicemia and hyperbilirubinemia, not related to treatment.
- One in the FE study, owing to disease progression, not related to treatment.

In summary, there is an overall consistency in safety profile of niraparib among different clinical studies; its managable safety and good tolerance meet the expectations of the drug's mechanism of action, and is comparable to similar drugs.

#### 1.1.4.3 Pharmacokinetics Data

The pharmacokinetics (PK) of Niraparib has been evaluated in the following studies:

- Single- and multi-dose PK (PN001)
- AME (PR-30-5015-C)
- Two sub-studies included in the NOVA study: QT/QTc (PR-30-5015-C1-QTc) and food effects (PR-30-5015-C2-FE)

The pharmacokinetic characteristics of Niraparib are summarized as below.

Within the dosage range of 30 mg-400 mg, Niraparib is rapidly absorbed after oral administration (time to peak [T_max_], about 2 to 4 hours); the average half-life (t_1/2_) is 48 to 60 hours in most cases; the dose-exposure (area under blood concentration-time curve [AUC] and maximum concentration [C max]) reveals a linear proportion relationship; the accumulation of Niraparib is dose-independent at steady state (accumulation coefficient R, about 1.5-4); and there is an overall consistency in Tmax and t_1/2_ at all dose levels, suggesting that drug absorption and clearance are dose-independent. The coefficient of variation among individuals for the main PK parameters is 30-70%.

Niraparib binds to human plasma protein at moderate level (83.0%), and its apparent volume of distribution (Vd/F) is 1220 L, suggesting that Niraparib is widely distributed in body tissues.

Hepatic clearance and renal excretion are the main ways to eliminate this drug product from the human body. The metabolic clearance of Niraparib in the liver is mainly achieved through its hydrolysis by non-CYP enzymes, especially carboxylesterase, to form inactive carboxylic acid metabolite (M1). M1 in turn undergoes further metabolism, mainly glucuronidation. The elimination of the compound is relatively slow; no products metabolized by the CYP enzymes has been found. a non-CYP enzyme substrate, Niraparib is unlikely to interact with various CYP enzymes, therefore, concomitant use of CYP enzyme inhibitor or inducer will not change the PK characteristics of Niraparib. In addition, the probability of interaction between niraparib and drug transporters, especially all major uptake transporters and efflux transporters, is low. Therefore, Niraparib can be used in combination with other drugs without dose adjustment for fear of drug interactions. Population pharmacokinetic studies have showed that there is no need to adjust the dose in patients with mild to moderate liver dysfunction, and mild and moderate renal impairment has no significant effect on the pharmacokinetics of Niraparib.

Niraparib has a highly oral bioavailability (~73%); and its absorption is not significantly affected by a high-fat diet, so it can be taken either before or after meals.

Research on QTc demonstrated that therapeutic dose of Niraparib did not prolong the QT interval. The baseline change in QTc was not associated with the plasma exposure level of Niraparib.

#### 1.1.4.4 Clinical Efficacy Data

The NOVA study has enrolled 553 patients with platinum-sensitive recurrent ovarian cancer from 107 sites around the world, including 203 patients in the g*BRCA* mutation cohort, and 350 patients in the non-g*BRCA* mutation cohort, among which 162 patients had tumor homologous recombinant deficiency-positive (HRDpos), 134 had HRD-negative (HRDneg) cancer, and 54 were HRD status unknown (HRDnd). Maintenance treatment with Niraparib 300 mg QD significantly prolonged PFS of patients compared with those in the placebo arm. [Table 5](#_Hlk486521526) shows that Niraparib had statistical and clinical significance in PFS, the primary efficacy endpoint, in three primary efficacy populations.

Table 5 - PFS of Ovarian Cancer Patients in NOVA Study Based on Independent Center Review (ITT Population)

| **Statistic items** | **g*BRCA*mut cohort** | | **non-g*BRCA*mut cohort** | | | |
| --- | --- | --- | --- | --- | --- | --- |
|  |  |  | **HRDpos** | | **All** | |
|  | **Niraparib**  **(N=138)** | **Placebo**  **(N=65)** | **Niraparib**  **(N=106)** | **Placebo**  **(N=56)** | **Niraparib**  **(N=234)** | **Placebo**  **(N=116)** |
| Median PFS (month) | 21 | 5.5 | 12.9 | 3.8 | 9.3 | 3.9 |
| (95%CI) | (12.9, NE) | (3.8, 7.2) | (8.1, 15.9) | (3.5, 5.7) | (7.2, 11.2) | (3.7, 5.5) |
| P value | <0.0001 | | <0.0001 | | <0.0001 | |
| Hazard ratio | 0.27 | | 0.38 | | 0.45 | |
| (95%CI) | (0.173, 0.410) | | (0.243, 0.586) | | (0.338, 0.607) | |

*Abbreviation – BRCA= Breast cancer susceptibility gene; CI= confidence interval; gBRCAmut= Germline BRCA mutation; HRDpos=Homologous recombination defect positive; ITT= Intent-to-treat population; NE=not evaluated; non-gBRCAmut= Non-germline BRCA mutation; PFS=progression-free survival*

Data from the patient-reported outcome shows that there was no significant difference in life-related quality indicators between Niraparib and placebo; Niraparib treatment did not affect the effectiveness of subsequent treatment, and the secondary effective indicator PFS2 (interval from the randomization of NOVA study to second disease progression on next-line anti-cancer treatment after the completion of study treatment) indicated the continuation of the effectiveness of Niraparib: median PFS2 is 22.3 months (95% CI: 18.6, NE) and 17.6 months (95% CI: 12.9, NE) in Niraparib group and placebo group, respectively, and the hazard ratio is 0.65 (95% CI: 0.372, 1.124) (p=0.1200); additionally, though the overall survival (OS) of Niraparib treatment was not mature, it was not negative.

See IB for further information of Niraparib.

## 1.2 Research Principle and Risk/Benefit Evaluation

Most patients with ovarian cancer will relapse within 2 years after treatment with platinum-based regimens. Patients with platinum-sensitive recurrent ovarian cancer (PSROC) will usually continue to receive platinum-based chemotherapy, and then passively wait for the almost inevitable disease recurrence. Currently no effective maintenance treatment has been approved for this patient population in China, so there is an urgent need for new treatment options for PSROC patients to improve duration of remission after the platinum-based treatment.

According to phase I/II clinical studies, PARP inhibitor is an effective maintenance treatment for g*BRCA*mut PSROC patients, and an effective single agent treatment for g*BRCA*mut recurrent ovarian cancer patients who have received at least 3 lines of chemotherapy^9, 10^.

The NOVA study is the first completed pivotal phase III clinical trial of PARPi, which confirmed that Niraparib as maintenance therapy could significantly prolong PFS in patients with PSROC; the drug was extensively beneficial to PSROC patients, both with g*BRCA* mutation and non-g*BRCA*mutation^11^. Based on these data, the US FDA granted the drug qualifications for fast track approval and priority review in September and December of 2016; and in March of 2017, the drug was rapidly approved by FDA for marketing 3 months in advance as maintenance treatment for platinum-sensitive recurrent ovarian cancer.

According to NOVA study, maintenance treatment with Niraparib had significantly prolonged PFS and reduced the risk of disease progression or death in PSROC patients; it had no significant adverse effect on the quality of life and subsequent anti-tumor therapy; the drug had overall manageable safety and good tolerability, and common adverse reactions of system organ classification (including gastrointestinal disorders and hematological abnormalities) could be effectively controlled by symptomatic supportive care and/or dose modification. In this study, rigorous safety monitoring, effective dose modification and other applicable measures will be performed to reduce the potential risk of the drug.

Based on the above benefit-risk assessment of ZL-2306 (Niraparib) in the target population of platinum-sensitive recurrent, high-grade serous ovarian cancer patients, we believe that it is appropriate to conduct this phase III, randomized, double-blind, multicenter clinical study to evaluate the efficacy and safety of Niraparib versus placebo as maintenance treatment for patients with platinum-sensitive recurrent ovarian cancer in China.

# 2 Study Objectives

## 2.1 Primary Objective

The primary objective of this study is to evaluate the efficacy of ZL-2306 (Niraparib) as maintenance treatment for PSROC by comparing the difference of tumor progression-free survival (PFS) between ZL-2306 (niraparib) and placebo.

## 2.2 Secondary Objective

The secondary objective of this study is to evaluate other clinical benefits compared to placebo, including chemotherapy-free interval (CFI), time to first subsequent treatment (TFST), overall survival (OS) and safety and tolerability.

# 3 Study Plan

## 3.1 Study Design

**Figure 1 – Design and Flowchart**


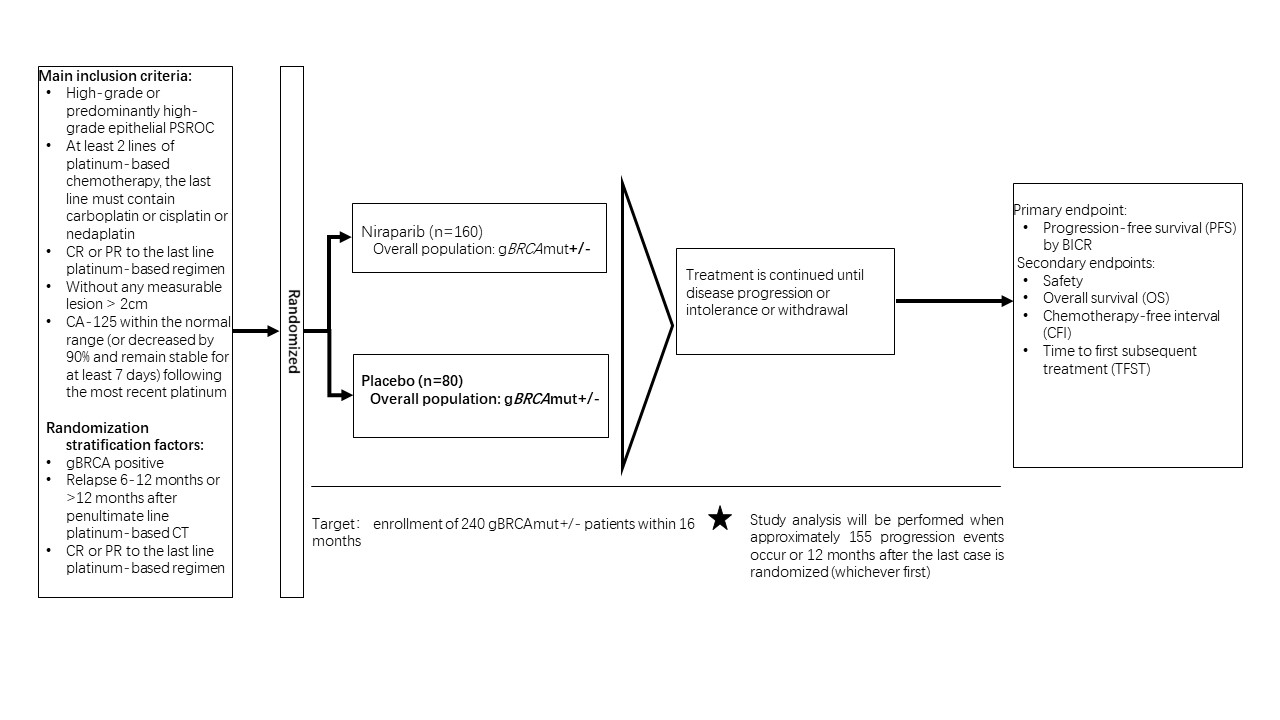


This phase III, randomized, double-blind, placebo-controlled, multicenter clinical trial is designed to evaluate the efficacy and safety of ZL-2306 (Niraparib) versus the placebo as maintenance treatment in patients with platinum-sensitive recurrent ovarian cancer. The subjects enrolled shall meet the following inclusion criteria:

1. Histologically diagnosed high-grade serous or predominantly high-grade serous epithelial OC (no histological restrictions for OC patients harboring germline BRCA mutations);
2. Having received at least 2 lines of platinum-based chemotherapy and achieved response to the penultimate line platinum-based regimen (CR or PR) but relapsed after 6 months, i.e. platinum sensitive recurrence;
3. Having received at least 4 cycles of last-line (the most recent line) platinum-based (must be carboplatin or cisplatin or nedaplatin) combination after the relapse and achieved response (CR or PR), without any measurable lesion > 2cm (eligibility of lesion > 2cm shall be discussed with the sponsor based on evidence of PR), and CA-125 shall be within the normal range (or decreased by 90% and remain stable for at least 7 days) following the most recent platinum-based chemotherapy.

Approximately 240 PSROC patients will be enrolled in this study (regardless of g*BRCA* mutation, but g*BRCA* mutation will be taken as one of the factors for randomized stratification). They will be randomized assigned in a 2:1 ratio to receive ZL-2306 (niraparib) and placebo once daily.

The primary endpoint of this study is PFS, as assessed by an independent review committee. The analysis of all patients enrolled (about 240) will be performed at the time of about 155 progression events or 12 months after the randomization of the last case (whichever occurs first). Type I error is set at 0.05 (two-sided), and the overall false positive rate controlled at 0.05.

The sample size is calculated based on a hazard ratio of 0.54 for all subjects (The median PFS in ZL-2306 [Niraparib] group and placebo arm are assumed to be 9.3 months and 5.0 months respectively).

Subjects are required to submit a blood sample to the designated central laboratory for gBRCA testing before enrollment. The patient's gBRCA status must be known.

The interpretation of g*BRCA* test results and the basis of randomization stratification are shown in [Table 6](#_Hlk486575989).

Table 6 – Interpretation of gBRCA Test Results and Randomization Stratification Basis

| **Results Reported** | **Randomization stratification basis** |
| --- | --- |
| Pathogenic mutation | g*BRCA* mutation positive |
| Suspected pathogenic mutation | g*BRCA* mutation positive |
| Mutation of unknown clinical significance | Non-g*BRCA* mutation positive |
| Suspected benign mutation | Non-g*BRCA* mutation positive |
| No mutation detected or benign polymorphism | Non-g*BRCA* mutation positive |

Randomization stratification factors include:

- g*BRCA* mutation positive
- Relapse 6-12 months or ≥12 months after penultimate platinum-based therapy
- Response to the most recent platinum-based chemotherapy (CR or PR).

After double-blind randomization, the patients will begin to receive maintenance treatment with ZL-2306 (Niraparib) or matched placebo. The initial dose is determined based on the subject's baseline body weight or baseline platelet count (but dose modification after initiation will not be based on body weight):

- For patients with baseline body weight ≥77 kg **and** baseline platelet count ≥150 × 10^3^/µL, the starting dose is ZL-2306 (niraparib) 300 mg (3× 100 mg capsules) or matched dose of placebo (3 capsules);
- For patients with baseline body weight <77 kg or baseline platelet count <150 × 103/µL, the starting dose is ZL-2306 (niraparib) 200 mg (2× 100 mg capsules) **or** matched dose of placebo (2 capsules). For patients with a starting dose of 200 mg (2 capsules), if there is no dose interruption or reduction during the first two cycles of treatment, the dose can be adjusted to 300 mg (3 capsules).

Every 28 days is a treatment cycle. Outpatient visit will be conducted once a week in the first cycle, and then once every cycle (4 week±5 days). The patients will continue to receive the assigned treatment until disease progression (RECIST v1.1), death, withdrawal of consent or lost to follow-up. Dose interruption or reduction due to any intolerable toxicity is allowed according to the principle of dose modification prescribed in the protocol:

- For patients with an initial dose of 300mg/d, the dose can be reduced to 200mg/d or further reduced to 100mg/d, which is the minimum dose and no further reduction is allowed (unless the investigator believes that the patient may continue to benefit from further reduction and the reduction requires permission from the sponsor’s medical monitor);
- For patients with an initial dose of 200mg/d, it can be reduced to 100mg/d which is the minimum dose and no further reduction is allowed (unless the investigator believes that the patient may continue to benefit from further reduction, and the reduction requires permission from the sponsor’s medical monitor).

Patient may interrupt treatment if toxic reactions occur, but interruption over 28 days warrant withdrawal from the study.

CT or MRI scans of the subject's abdomen / pelvis and clinically indicated sites will be performed and evaluated based on the RECIST v1.1 guidelines. The imaging evaluations are scheduled at the end of every 2 cycles (8 weeks ± 7 days) till the end of the 14^th^ cycle (56 weeks), and then at the end of every 3 cycles until disease progression. The tumor imaging evaluation shall be performed in accordance with the time frames specified above, and the counting of treatment cycles will not be affected by any treatment interruption. If the treatment termination is not due to disease progression, death, withdrawal of consent or lost to follow-up, the tumor imaging evaluation is still to be performed according to the pre-defined interval, until disease progression, or subsequent anti-cancer treatment. Patients with disease progression will be followed up for their survival status by telephone visit at least once every 3 months until the data cut-off date of the overall survival analysis or patient death.

A Data Monitoring Committee (DMC) will be established to systematically review and evaluate safety to safeguard the benefit and safety of the subjects. The DMC will consist of 3 members independent of the Sponsor, including 1 biostatistician and 2 physicians. It will regularly provide the sponsor with recommendations to continue or terminate the study based on periodic assessment of safety information. The DMC's membership, key responsibilities and related operating procedures are detailed in the DMC Charter.

## 3.2 Discussion on Design of Research

This phase III, randomized, double-blind, placebo-controlled, multicenter clinical study is designed to evaluate the efficacy and safety of ZL-2306 (Niraparib) as maintenance treatment for patients with platinum-sensitive recurrent ovarian cancer (PSROC). In view of the good clinical efficacy data of Niraparib in phase III clinical trials around the world, enrolled patients will be randomized to receive ZL-2306 (niraparib) and placebo in a 2:1 ratio so that more patients may benefit from the study treatment, and sufficient safety data can be obtained. Given that currently there is no drug approved as maintenance therapy for PSROC in China, the study will adopt placebo as the control arm.

**Selection of Patient Population:**

Enrolled patients shall have received at least 2 lines of platinum-based chemotherapy and achieved response to the penultimate line platinum-based regimen but relapsed, and achieved CR or PR to the most recent line of platinum-based chemotherapy. It is planned in this patient population to evaluate the efficacy and safety of ZL-2306 (Niraparib) as maintenance treatment. The NOVA study has shown that this agent can improve PFS (the primary endpoint), regardless of g*BRCA* mutation status. As currently there is no approved effective maintenance therapy for this patient population in China, this study will fill the gap in this area. Given that the patients with g*BRCA* mutation is more sensitive to PARR inhibitor, this study will prospectively detect g*BRCA* mutation status, and take g*BRCA*mut as a randomization stratification factor, making it possible to conduct a further research in patients with positive g*BRCA* mutation, on the basis of a broad patient population.

In ovarian cancer (OC) patients, the main and primary drug treatment is platinum-based chemotherapy, whose mechanism of action is to cause extensive intrastrand cross-linking of DNA. The effective repair of this type of damage requires a normal homologous recombination pathway. Defects in this pathway in many OC patients due to germline or somatic BRCA mutations are also the molecular basis of their sensitivity to PARP inhibitors. Platinum sensitivity and PAPR inhibitor sensitivity may share common characteristics in the homologous recombination pathway. So platinum-sensitivity may be useful in the enrichment of patients who are sensitive to PARP inhibitor^12,13^. The hypothesis that PARP inhibitor is more effective for platinum-sensitive than platinum-resistant OC, is supported by the data from the NOVA study and clinical trials on similar drugs, such as Olaparib^14^. Therefore, platinum-sensitivity can be used as a potential clinical indicator to enrich patients who are sensitive to PARPi, i.e. PSROC patients (relapse ≥6 months after platinum-based chemotherapy, and response to the most recent line platinum), as required in this study.

**Choice of Efficacy Endpoints:**

This study will use PFS rather than overall survival (OS) as the primary endpoint. OS may be affected by many confounding effects such as subsequent multiple or cross-over treatments, requires a long follow-up period, and may be complicated by many non-cancer cause of death. In contrast, PFS has been extensively recognized and accepted by FDA, EMA and other agencies as the primary endpoint in the studies on maintenance treatment of ovarian cancer, especially in the double-blind study design, it can provide sufficient evidence for clinical benefits^15,16^. In this double-blinded study all images and clinical information will be assessed by blinded independent central review (BICR) to determine disease progression and PFS. Secondary endpoints will include chemotherapy-free interval (CFI), time to first subsequent treatment (TFST) to evaluate the effect of Niraparib maintenance on subsequent therapy. OS will also be included as a secondary endpoint to investigate if there is a diminishing effect on survival. Given that the median OS for this patient population is more than 2 years, follow-up will be continued to collect OS information. The decision whether to conduct OS analysis will be based on survival data from clinical studies of this study drug and/or similar products. If the decision to perform OS analysis is made, the final OS analysis will be performed when approximately 135 deaths have occurred in the ITT population (at least 50% data maturity) or at least 48 months after the primary analysis readout, whichever comes first.

## 3.3 End of study

After the primary analysis results are available in this study, unblinding will be performed for all patients. At this point, the main study ends, and after assessment by the investigator and discussion with the sponsor, ZL-2306 (niraparib) will be provided to eligible patients.

During the period when patients continue receiving ZL-2306 (niraparib), adverse events of special interest (AESIs) must be reported in accordance with the procedures and timelines specified in Section 8.3.8. SAEs must be reported in accordance with the procedures specified in Section 8.3.9 until 30 days after the last dose. After discontinuation of ZL-2306 (niraparib), survival follow-up will be conducted every 90 days (±7 days), collecting information on the first subsequent anticancer treatment, including chemotherapy. The sponsor has the right to request further data if necessary.

End of study is defined as the occurrence of progressive disease in the last subject or any other situation requiring termination of the study drug treatment or a decision by the sponsor to terminate the study.

# 4 Study Population

## 4.1 Inclusion Criteria

Enrolled patients must meet all of the following criteria:

1. Written informed consent before any study-related procedure.
2. Agree to receive g*BRCA* mutation test (result must be known before randomization).
3. Women aged 18 years or older.
4. Histologically confirmed epithelial ovarian cancer, fallopian tube cancer or primary peritoneal cancer.
5. High-grade (Grade 3) serous or predominantly high-grade serous ovarian cancer (no histological restrictions for patients with ovarian cancer carrying germline BRCA mutations).
6. Having received at least two lines of platinum-based chemotherapy, and meet following criteria:
   1. After the penultimate platinum-based chemotherapy, the patient shall meet the following requirements:
   - Clinically CR or PR to this line of chemotherapy.
   - Relapse after this course of chemotherapy must be PSROC, i.e., disease does not progress until 6 months after the course of chemotherapy (on medical records 6-12 months or ≥ 12 months).
   1. After the most recent line of platinum-based chemotherapy, the patient shall meet the following requirements:
   - Having received at least 4 cycles of platinum-containing chemotherapy (must be carboplatin or cisplatin or nedaplatin).
   - Clinical response to this line of chemotherapy must be CR (i.e., after this line of chemotherapy, there is no imaging measurable or unmeasurable lesion according to RECIST v1.1, and CA-125 is within the normal range) or PR (after this line of chemotherapy at least 30% reduction in total sum of diameter of targe lesion compared to the baseline before chemotherapy).
   - CA-125 level is within the normal range after the chemotherapy, or decreases > 90% during the course of chemotherapy and remains stable for at least 7 days (increase in CA-125 level before enrollment should not exceed 15% relative to the level after chemotherapy).
   - No measurable lesion> 2cm (measurable disease >2cm shall be discussed with the sponsor based on evidence of PR).
   1. The patient shall be randomized within 8 weeks after completion of most recent line platinum-containing chemotherapy.
7. ECOG performance status 0 or 1.

8. Adequate organ function, including

- 1. Neutrophil count ≥1500/µL
  2. Platelet count ≥100,000/µL
  3. Hemoglobin ≥10g/dL
  4. Serum creatinine ≤ 1.5 × ULN, or creatinine clearance rate ≥ 60mL/min (using Cockcroft-Gault equation)
  5. Total bilirubin ≤1.5 × ULN, or direct bilirubin ≤1.0 × ULN.
  6. AST and ALT ≤ 2.5 × ULN, or ≤ 5 × ULN in case of hepatic metastases.

9. Women of childbearing age with a negative pregnancy test at the time of enrollment and have promised to take adequate and effective contraceptive measures or abstinence during the study period and within 3 months after the last dose of study drug. Or women without potential fertility, defined as below:

a. Having received surgical sterilization operation (such as hysterectomy, bilateral ovariectomy, or bilateral tubectomy); or

b. ≥60 years of age; or

c. ≥40 and <60 years of age, more than 12 months of menopause, and follicle-stimulating hormone (FSH) are within the post-menopause reference range.

10. Ability to comply with the protocol.

11. The level of any toxicity due to previous chemotherapy has decreased to ≤CTCAE grade 1 or baseline level, except for ≤CTCAE grade 2 stable sensory neuropathy or alopecia.

## 4.2 Exclusion Criteria

A patient shall be excluded from the study if any one of following criteria is met:

1. Known hypersensitivity to active or inactive components of ZL-2306 (niraparib) or drugs with similar chemical structures.
2. Previous treatment with PARP inhibitor.
3. Drainage of ascites during last 2 cycles of last chemotherapy prior to enrollment
4. Symptomatic uncontrollable brain or leptomeningeal metastases. No imaging scan is required to confirm the absence of brain metastases; patients with spinal cord compression who have received targeted treatment and evidence of clinical stability of the disease for at least >28 days can still be considered for enrollment (in case of controlled central nervous system metastasis, relevant treatment such as radiotherapy or chemotherapy must be given at least 1 month before the study; no new CNS-related symptoms or symptoms indicating disease progression, and either are taking a stable dose of steroid, or do not need hormone therapy).
5. Having received a major surgery 3 weeks before entering the study, or not yet recovered from the surgical effect.
6. Having received a palliative radiotherapy covering >20% of bone marrow within 1 week prior to enrollment.
7. Invasive cancers other than ovarian cancer within 2 years prior to enrollment (except for fully treated basal or squamous cell skin cancer).
8. A previous or current diagnosis of myelodysplastic syndrome (MDS) or acute myeloid leukemia (AML).
9. Other serious or uncontrolled diseases, including but not limited to:

a. Uncontrollable nausea and vomiting, inability to swallow study drug, and any gastrointestinal disease that may interfere with the absorption and metabolism of the drug

b. Active viral infections, such as human immunodeficiency virus, hepatitis B virus, hepatitis C virus and so on

c. Uncontrolled ventricular arrhythmias and recent myocardial infarction within 3 months

d. Uncontrolled major seizure disorder, unstable spinal cord compression, superior vena cava syndrome or any other psychiatric disorders that prohibits obtaining informed consent

e. Immunodeficiency (except for splenectomy), or other diseases that investigators believe may expose patients to high-risk toxicity.

1. Any previous or current disease, treatment or lab abnormality that might interfere with the results of the study, the patient’s full participation during the study, or the investigator believes that the patient is not suitable for participating in this study; platelet or red blood cell transfusion is not allowed within four weeks before initiation of study treatment.
2. Pregnancy or breast feeding, or planning a pregnancy during the study.

## 4.3 Previous and Concomitant Medication

Any medication other than the study treatment, including prescription, over-the-counter and herbal medicine and dietary supplements, etc., is considered as a concomitant medication. Previous medication refers to all the medications used before the start of the study medication (medications terminated before the first dose of study drug). Concomitant medication refers to any drug treatment other than the study treatment, which starts on or after the first dose of study drug, including those that started before the trial and continue to be used after the trial starts.

All previous and concomitant medications must be recorded in the eCRF, including the following information related to the medication: generic name, route of administration, start and end dates, dosage and indications. Any change in the dosage or drug used of a concomitant medication must also be recorded in the eCRF. At screening, the patients will be asked what medications have taken in the past 30 days. At each subsequent study visit, the patients will also be asked what concomitant medications they are currently taking.

### 4.3.1 Restrictions on Concomitant Treatment

Sexually active patients with fertility potential and their partners must take two effective form of contraception from the beginning to the end of the study and within 3 months after the last dose of study treatment.

1. Spermicide-containing condom and 1 of the following:

- Oral contraceptive or hormone therapy (e.g., hormone implants)
- Placement of an intra-uterine contraceptive device（IUD）

Acceptable non-hormonal birth control methods include:

1. Total sexual abstinence. The sexual behaviour must be abstained during the entire study period, that is, from the moment when the ICF is signed to 90 days after the last dose of the study drug;
2. Male sexual partners who have undergone vasectomy and are currently using male condoms with spermicides, and need to confirm that there is no sperm after vasectomy.
3. Fallopian tube occlusion plus male condom with spermicide.
4. Intrauterine device (IUD) plus male condom with spermicide.

Acceptable hormone-based methods include:

1. Etonogestrel implants (e.g., Implanon, Norplan), combined with male condom with spermicide.
2. Normal and low dose combined oral pill, combined with male condom with spermicide.
3. Methyl progesterone/ ethinyl estradiol (EE) transdermal system, combined with male condom with spermicide.
4. Intra-vaginal device (e.g. EE and etonogestrel), combined with male condom with spermicide.

During the study, no other anti-cancer therapy (including Chinese herbal medicine with anti-cancer effect) is allowed. ZL-2306 (Niraparib) has the potential to induce CYP1A2, so caution shall be taken when taking CYP1A2-sensitive substrates (seeing [Appendix 1](#附件1)). Since ZL-2306 (Niraparib) may result in thrombocytopenia, so anticoagulant or antiplatelet drugs shall also be used with caution. In the absence of disease progression, palliative radiotherapy is allowed for small areas of painful metastases that exists before the enrollment and cannot be managed with by local or systemic analgesics (but pelvic radiotherapy and/or palliative radiotherapy covering more than 20% of the bone marrow within 1 week before the enrollment are not allowed); preventive use of cytokines (granulocyte colony-stimulating factor [GCSF], etc.) is not allowed in the first treatment cycle of the study, but it can be administered after the first cycle per local clinical practice. Conventional chemotherapy drugs increase the risk of infection when vaccinated with live-virus or bacterial vaccines. Effect of ZL-2306 (Niraparib) are unknown, so patients are not allowed to be inoculated with live-virus/bacterial vaccines in this study.

## 4.4 Handling of Subjects Enrolled Incorrectly

Patients who do not meet the inclusion criteria are not allowed to enter this study, and there are no exceptions to this rule. Subjects who are found to be ineligible after enrollment are not allowed to receive any study treatment and must withdraw from the study. In the event that a study drug treatment is mistakenly administered to an ineligible subject, the investigator shall notify the Sponsor immediately, and both parties shall discuss whether the patient's study treatment should be terminated immediately or continued, and the decision shall be documented in detail.

## 4.5 Discontinuation from Treatment

### 4.5.1 Discontinuation from Treatment

Patients may be discontinued from treatment for the following reasons:

- Adverse event
  - Any treatment-related CTCAE grade-3/4 adverse events, which have not reverted to CTCAE grade 1 or lower within 4 weeks (28 days) of dose interruption
  - Dose reduction is at the investigator's discretion, and must comply with the dose interruption regulations (≤28 days); the maximum number of 2 does reduction is allowed. If any CTCAE grade-3/4 adverse events still recur at the lowest allowable dose, the investigator must discuss with the sponsor whether treatment should be discontinued according to the patient's condition.
  - If the adverse event is thrombocytopenia, and the platelet count have not reverted to >100,000/µL within 4 weeks (28 days) of dose interruption, the patient must be discontinued;
- Disease progression as assessed according to RECIST 1.1 criteria
- Risk to patient as judged by the investigator and/or Sponsor
- Serious violation of the study protocol as judged by the investigator and/or Sponsor
- Patient request
- The patient becomes pregnant

The patients who discontinue from the treatment due to reasons listed above will continue to receive follow-up assessments (such as CFI, TFST and OS), unless the patients discontinued from the study because of the conditions listed below.

### 4.5.2 Withdrawal from Study

All subjects may discontinue from this study at any time, regardless of whether a reason is provided or not; and they will not be discriminated or unfairly treated, and their medical treatment will not be affected.

Patients who discontinue from the study under one of following conditions will not be subject to the further data collection:

- Withdrawal of informed consent
- Death by any death
- Lost to follow-up

Subjects should complete the end-of-therapy visit (EOT) when they discontinue study treatment or withdraw from the study. The research center shall immediately inform the inspector of the Sponsor and record the date and cause of treatment discontinuation and the withdrawal from study.

If a patient withdraws voluntarily early and does not want to return to the site to complete the final visit, the investigator should make every effort to contact the patient. The investigator shall ask the reason for the early withdrawal, and if feasible, request them to return all unused study drugs and supplies as much as possible and to complete the last visit, as well as follow up and treat any unresolved adverse events.

In any case, the patient's final condition shall be recorded as comprehensively as possible, especially the patient's survival status (secondary study endpoint).

If a patient withdraws from the trial and requests not to disclose their future information, no examination is necessary. However, the Sponsor may still retain and use the pre-existing information.

The study will not replace subjects in the event of any early withdrawal.

### 4.5.3 Unblinding

This is a randomized, double-blind, phase III placebo-controlled clinical trial. In order to ensure the integrity and objectivity of the data, the study design and implementation will be conducted in accordance with the standard operation of double-blind controlled study.

Before the primary analysis results are available in this study, subjects and investigators shall be blinded, expect for medical emergency such as treatment-related adverse event (AE)/ serious adverse event (SAE). The study physician of Sponsor may at any time assist the investigator in making the patient unblinded; and once the blind is broken, the investigator shall document the reason in the electronic case report form (eCRF) page and the unblinded patient will withdraw from the study permanently.

After the primary analysis results are available in this study, unblinding will be performed for all subjects. For subjects who remained on ZL-2306 (niraparib), based on the investigator’s assessment and discussion with the sponsor, if there is clinical benefit, the patient may continue taking ZL-2306 (niraparib). For subjects who remained on the placebo, based on the investigator’s assessment and discussion with the sponsor, if there is a possibility of clinical benefit from taking ZL-2306 (niraparib), the patient were allowed to receive ZL-2306 (niraparib).

After unblinding, all patients who continue receiving ZL-2306 (niraparib) will undergo safety monitoring and efficacy assessment according to the medical practices of their respective hospitals until progressive disease or the occurrence of intolerable adverse events or other circumstances requiring treatment discontinuation as specified in the study protocol (see Section 4.5.1 of this protocol). The sponsor has the right to request safety or efficacy data if necessary.

## 4.6 Identification Number of Subjects and Randomized Assignment

### 4.6.1 Identification Number of Subjects

All subjects entering the screening period of the study (defined as the point at which the patients signs of the informed consent) will receive a unique patient identification number from the Interactive Web Response System (IWRS). This number will be used to identify the subjects during the study and record all study documents related to the patient. The number must be consistent throughout the entire study, and it must not be changed at the time of enrollment or randomization. This number will not necessarily be the same as randomization number for the study.

### 4.6.2 Randomization Scheme

Subjects will be randomized to receive ZL-2306 (Niraparib) or placebo in a 2:1 ratio on the same day of or 72 hours prior to the first dose. The randomization stratification factors include g*BRCA* mutation status, time to relapse following penultimate platinum therapy (relapse after 6-12 months, or ≥12 months) and response to the most recent platinum-based chemotherapy (CR or PR).

The randomization schedule is prepared by IWRS supplier, through a validated standard procedure. Subjects who have completed all study screening assessments and met all eligibility criteria will receive a random number generated by IWRS, which will link them to the designated treatment group (ZL-2306 [Niraparib] group or placebo group), and can be used as the reference to determine the amount of study products needed to be distributed. The randomization number of the subject who withdrew from the study for any reason will be retained. IWRS staff will only prepare the randomization schedule and will not involve in any trial operation.

## 4.7 Planned Numbers of Patients and Study centers

PFS is the primary endpoint of this study. It will be analyzed based on approximately 240 patients (including g*BRCA*mut carriers and non-g*BRCA*mut carriers). These patients will be randomized to receive ZL-2306 (Niraparib) or placebo in a ratio of 2:1, and the enrollment is planned to complete within 16 months. Studies have shown that the proportion of patients carrying gBRCA mutations in patients with platinum-sensitive recurrent ovarian cancer (PSROC) is about 20%-28%. In order to ensure that the population of patients enrolled in this study can truly reflects the PSROC patient population, real-time monitoring will be performed during the enrollment process; and if necessary, it may be possible to adjust the enrollment speed of patients with non-g*BRCA* mutations to ensure that the proportion of g*BRCA*mut carriers is not less than 20% of the enrolled patients. Therefore, at least 240 patients will be enrolled, and the study is planned to be conducted in 30 qualified research centers.

# 5 Study Treatment

The study drug is ZL-2306 (Niraparib) oral capsule (100 mg/cap), and a matched placebo with similar appearance.

## 5.1 Study Treatment Regime

The patients will be randomized to receive ZL-2306 (niraparib) or matched placebo in a 2: 1 ratio. The initial dose is determined based on the subject's baseline body weight or baseline platelet counts (but dose modification after initiation will not be based on body weight):

- For patients with baseline body weight ≥77 kg and baseline platelet count ≥150,000 /µL, the starting dose is ZL-2306 (niraparib) 300 mg (3 × 100 mg capsules) or matched dose of placebo (3 capsules);
- For patients with baseline body weight <77 kg or baseline platelet count <150,000/µL, the starting dose is ZL-2306 (niraparib) 200 mg (2 × 100 mg capsules) or matched dose of placebo (2 capsules). For patients with a starting dose of 200 mg (2 capsules), if there is no dose interruption or reduction during the first two cycles of treatment, the dose can be adjusted to 300 mg (3 capsules).

Each subject will orally take the prescribed dose of ZL-2306 (Niraparib) or matched placebo once a day; the number of capsules taken can be adjusted accordingly for any dose modification during the study. It is recommended to take the medicine at the same time every day, preferably in the morning, with water or food. The entire capsule shall be swallowed, not broken, chewed or opened; eating and drinking are allowed before and after taking the medicine. The patients should be informed that if they missed the planned dose (for example, if they forgot to take it) or vomited after taking the drug, they should not take the drug again, but take it at the next scheduled time. Any study drug intteruption/discontinuation and dose reduction as well as their reasons shall be recorded in detail in the eCRF.

In the event of an adverse event caused by the trial treatment, the investigator can modify the dosage according to the dose adjustment principle specified in this protocol, that is, temporary interruption (up to 28 days) or dose reduction (as shown in [**Table 7**](#_Hlk486577230)).

- For patients with an initial dose of 300 mg/d, the dose can be reduced to 200 mg/d or further reduced to 100 mg/d, which is the minimum dose and no further reduction is allowed (unless the investigator believes that the patient may continue to benefit from further reduction, and it requires permission from the sponsor’s medical monitor);
- For patients with an initial dose of 200 mg/d, it can be reduced to 100 mg/d which is the minimum dose and no further reduction is allowed (unless the investigator believes that the patient may continue to benefit from further reduction, and it requires permission from the sponsor’s medical monitor).

Table 7 – Dose Adjustment Scheme for ZL-2306 (Niraparib)

| **Dose level** | **Starting dose is 300 mg QD** | **Starting dose is 200 mg QD** |
| --- | --- | --- |
| Initial dose level | 300 mg QD | 200 mg QD^1^ |
| Initial dose level -1 | 200 mg QD^3^ | 100 mg QD^2,3^ |
| Initial dose level -2 | 100 mg QD^2,3^ | NA |

^1^ For patients with a starting dose of 200 mg, on the premise that there is no dose interruption or dose reduction due to adverse reactions in the first two cycles of treatment, the starting dose may be increased to 300 mg.

^2^ If an adverse reaction requiring dose modification occurs at the 100 mg dose level, unless the investigator believes that the patient may continue to benefit from continued medication or further reductions, and permission from the sponsor’s medical monitor is required.

^3^ After reduction of the study drug from 200 mg QD to 100 mg QD, raising dose to 200 mg QD can be considered if well tolerated for at least one treatment cycle. After the study drug is reduced from the initial dose of 300 mg QD to 200 mg QD, the dose may be raised back to 300 mg QD if well tolerated for at least 2 cycles. Any increase in the dose of study drug after the dose reduction should be discussed with the sponsor’s medical monitor.

## 5.2 Properties of Study Drugs

Both ZL-2306 (Niraparib) and placebo will be provided in 100 mg capsule form. Drugs shall be stored at a 2-8^o^C condition. The research drug information used in this study is listed in [**Table 8**](#_Hlk486577276). Please refer to IB for more detailed information (including research drug ingredients).

Table 8 – Drugs for Study

| Drugs for study | Dose | Appearance | Administration mode | Manufacturer |
| --- | --- | --- | --- | --- |
| ZL-2306 (Niraparib) | 100 mg | Pink capsule | Oral | WuXi AppTec Co., Ltd.  Zai Lab (Suzhou) Co., Ltd. |
| Placebo | 100 mg | Pink capsule | Oral |  |

## 5.3 Package and Label

The 100 mg capsules are packed in aluminum-plastic blister packs. Each blister contains 15 capsules. The contents of the middle box label include: product name, specification, packaging, manufacturer, lot number, expiration, clinical protocol number and usage, etc.

## 5.4 Treatment Compliance

Patients are required to record study drug administration information in the research logs provided by the Sponsor. Patients will bring back the logs and all unused study drugs and empty boxes at each study visit, and the investigator and/or research staff will assess adherence to treatment through counting unused drugs and information provided by the patients or caregiver. At each visit, the study drug administration information (including planned administration dose, actual administration dose, dose change, dose suspension, etc.) must be recorded in the source file and administration dose eCRF. The investigator and other researchers are responsible for evaluating and analyzing the causes of potential poor adherence to treatment (AE or missed dose) with the medical monitor of Sponsor before and during the study, and consider and implement in advance proper strategies to improve the compliance.

## 5.5 Study drug reception, storage and management responsibilities

The investigator shall bear the responsibility for the management of all study drugs and equipments provided by the Sponsor. The investigator shall ensure that the study drugs and supplies are accurately recorded and meet the supply of test needs at any time, and keep, store, distribute, use and dispose of them, according to the study protocol and national laws and regulations. During the clinical trial, the investigator shall designate a responsible person to receive, store, dispense and return all the study drugs provided by the Sponsor and to accurately record the dispensing information of the study drugs. The drug dispensing management form should include the subject’s identification number, randomization number, the amount of dispensed drugs and vials, and the amount of drugs returned (need to be marked as "returned" to separate them from undistributed medicines).

All distribution and management records shall be available to the Sponsor for review. The research inspector will verify the amount of stored drugs against the drug dispensing management form. The designated drug manager will distribute the study drugs according to the study protocol and drug management manual, and assist the inspector to conduct their verification on time.

The subjects will be given the study drug on the first day of the first cycle as well as of every subsequent cycle, until the subject terminates the treatment or the study completes.

All study drugs must be stored at 2-8^o^C according to the manufacturer's instructions. They will be kept in a place which is controlled by the authority-based locking system, that meets the conditions of storage and comprehensive safety protection measures according to the requirements of the Sponsor.

## 5.6 Access to Study Drug upon Completion of Trial

The endpoint of this study and the treatment discontinuation criteria are disease progression, so there is no plan to provide patients with study drugs or other research interventions after the disease progression or after any early withdrawal for any reason.

After the end of the study, the Sponsor will assess the feasibility of continuing to provide ZL-2306 (Niraparib) to the patients who remained on the treatment, based on the evaluation of primary endpoint indicators and safety data collected in the study.

# 6 Visit Schedule and Evaluation

## 6.1 Evaluation of Study Visit

The time schedule of visits in the study is shown in [**Table 1**](#_Hlk486577971). All times should be recorded in a 24-hour format (e.g. 23:20, instead of 11:20 p.m.).

### 6.1.1 Screening period (visit 1, from day -28 to day -1)

The following procedures and tests will be performed at screening:

- The informed consent must be obtained from the patient prior to the commencement of any related process.
  - Routine examination results for ovarian cancer diagnosis & treatment (CT/MRI, CA-125, etc.) completed within the corresponding time limit required by the protocol (e.g., post-chemotherapy CT/MRI is performed within 28 days of the screening window period, and CA-125 is 7days before the first dose of study drug), and met all the testing requirements specified in the protocol, may be used for screening evaluation, if they are performed before informed consent.
- Demographics
- Medical history
  - Medical and surgical history
  - Detailed history of bone marrow suppression (thrombocytopenia, neutropenia, leukopenia or anemia) within 1 year before informed consent.
  - History of cancer diagnosis & treatment: date of first diagnosis, tumor type, disease stage at first diagnosis, histology and grading at first diagnosis, histology and grading of the most recent biopsy (if any), genotype including germline or somatic BRCA status, history of neoadjuvant chemotherapy and history of intraperitoneal chemotherapy. For patients with OC in first platinum-sensitive relapse, collect the following information: the start time of first treatment (1^st^ line treatment), the drugs used for the first treatment, the date of last dose in the first treatment, acceptance of or not maintenance therapy after first treatment and the drugs used, the start time of the 2^nd^ line treatment, the drugs used in the 2^nd^ line treatment, the date of the last dose in the 2^nd^ line treatment, best response to the 1st/2nd line treatment and the date of relapse after the 1^st^ line treatment. For patients who have received more than 2 lines of platinum-based chemotherapy, collect information on all lines of chemotherapy; collection of information about the penultimate/last line platinum-based chemotherapy include: the start time of the penultimate/last line platinum-based therapy, the drugs used in the penultimate/last line platinum-based therapy, the date of the last dose in the penultimate/last line platinum-based therapy, acceptance of or not maintenance after the penultimate line platinum-based therapy and best response to the penultimate/last line platinum-based therapy.
- Whole blood sample collection: whole blood samples will be collected and sent to the central laboratory designated by the Sponsor for g*BRCA* mutation test, the result must be obtained before randomization; previous g*BRCA* mutation may be used for randomization stratification before the central laboratory results are obtained, after discussion with the Sponsor.
- Serum or urine pregnancy test (within 7 days prior to first dose of study drug) (only for women of childbearing age)
- Physical examination (comprehensive assessment)
- Vital signs (breath, blood pressure, pulse, body temperature) and body weight
- Height
- Eastern Collaborative Oncology Group (ECOG) performance status
- Complete blood count (CBC) test
- Coagulation test (activated partial thromboplastin time [APTT], International normalized ratio [INR])
- Serum biochemistry
- Serum CA-125
- Urinalysis (must including: specific gravity, leukocyte esterase, nitrite, occult blood, protein, glucose, ketone body, urobilinogen and bilirubin)
- 12-lead ECG
- RECIST v1.1 tumor assessment - Abdomen/pelvic cavity and other clinically indicated sites
- Chest CT / MRI - if the site is not included in the RECIST v1.1 assessment, a chest CT/MRI scan is required; in the absence of any (suspected) lesion on chest CT/MRI at the time of screening that requires follow-up, there is no need to repeat the chest imaging examination in subsequent tumor assessment.
- Inquiry about AE
- History of previous and concomitant medication (detailed information on medication within 30 days prior to signing of the main ICF)
- Randomization (randomization is performed after all screening assessments have been completed and the eligibility of patients enrolled has been confirmed, and it will be completed within 72 hours prior to the first dose of study drug)

### 6.1.2 First Cycle

#### 6.1.2.1 Day 1 of the first cycle (C1D1)

- Physical examination (to assess any change from the screening period)
- Vital signs (breath, blood pressure, pulse, body temperature) and body weight
- ECOG performance status
- CBC (there is no need to repeat this test if it is done within 7 days before the study)
- Coagulation tests (there is no need to repeat these tests if they are done within 7 days before the study)
- Serum biochemistry (there is no need to repeat this test if it is done within 7 days before the study)
- Serum CA-125 (there is no need to repeat this test if it is done within 7 days before the study)
- 12-lead ECG (there is no need to repeat this examination if it is completed within 72 hours before randomization, and in the absence of related discomfort)
- Inquiry about AEs
- Inquiry about concomitant medications
- Distribution of study drug and medication log; the first dose is taken at the site

#### 6.1.2.2 Day 8 of the first cycle (C1D8)

- CBC (can be performed at local hospital)

#### 6.1.2.3 Day 15 of the first cycle (C1D15)

- Physical examination (changes since the previous assessment)
- Vital signs (breathing, blood pressure, pulse, body temperature) and body weight
- CBC
- Coagulation tests and serum biochemistry
- Inquiry about AEs
- Inquiry about concomitant medications

#### 6.1.2.4 Day 18 of the first cycle (C1D18)

- CBC (can be performed at local hospital)

If platelet is <100,000/μL at C1D15, the study drug must be interrupted, and this visit (C1D18) is not necessary, but all subsequent visits are still required, including C1D22.

#### 6.1.2.5 Day 22 of the firs cycle (C1D22)

- CBC (can be performed at local hospital)

### 6.1.3 Day 1 of the second cycle (C2D1)

- Physical examination (changes since the previous assessment)
- Vital signs (breathing, blood pressure, pulse, body temperature) and body weight
- ECOG performance status
- CBC
- Coagulation tests and serum biochemistry
- 12-lead ECG, before taking the medicine
- Inquiry about AEs
- Inquiry about concomitant medications
- Distribution and retrieval of study drugs and medication logs

### 6.1.4 Day 1 of subsequent cycles

- Physical examination (changes since the previous assessment)
- Vital signs (breathing, blood pressure, pulse, body temperature) and body weight
- ECOG performance status
- CBC (If the dose is interrupted or reduction due to hematotoxicity, a weekly CBC is required until the AE is relieved; if the study treatment is resumed after interruption for AE or the dose is increased, CBC must be performed at least once a week as shown in Table 11, at days 8, 15,18, 22, 29, then once every 4 weeks again. CBC can be performed at local hospitals, but other examinations and tests still need to be performed in the site)
- Coagulation tests and serum biochemistry
- Serum CA-125 - in conjunction with the tumor imaging assessment, once every two cycles until the end of the 14th cycle and then every three cycles until disease progression
- RECIST v1.1 assessment - Tumor assessment of the abdomen/pelvis and other clinical indicated sites will be performed according to RECIST 1.1 once every 2 cycles until the end of the 14^th^ cycle and then every 3 cycles.
- Inquiry about AEs
- Inquiry about concomitant medication
- Distribution and retrieval of study drugs and medication logs

## 6.2 Discontinuation of Study Treatment (Visit will be made within 7 days of last dose)

- Physical examination (changes since the previous assessment)
- Vital signs (breathing, blood pressure, pulse, body temperature) and body weight
- ECOG performance status
- CBC
- Coagulation tests and serum biochemistry
- Serum CA-125 - once every two cycles until the end of the 14^th^ cycle and then every three cycles until disease progression
- 12-lead ECG
- RECIST v1.1 assessment - Tumor assessment of the abdomen/pelvis and other clinical indicated sites will be performed according to RECIST v1.1 once every 2 cycles until the end of the 14^th^ cycle and then every 3 cycles. For patients who terminate treatment for a reason other than disease progression, the imaging assessment will continue to be performed as scheduled, until disease progression or withdrawal from the study.
- Inquiry about AEs (unrelieved or newly-emerged AEs from this visit to 30 days after the last dose of study drug must still be assessed and can be done by telephone; serious AEs or treatment-related toxicities must be followed up until AEs were recovery or stabilization)
- Inquiry about concomitant medications
- Distribution and retrieval of study drugs and medication logs

## 6.3 Post Study Treatment Assessments (subjects are not required to return to the site to complete the visit)

- Information on first subsequent anti-cancer treatment (including chemotherapy) after the study treatment will be collected every 90 (±7) days.
- Follow-up visit will be made every 90 (±7) days after study treatment discontinuation, to collect survival information, including information on new-onset malignant tumor.

## 6.4 Unplanned Follow-up Visit

Bone marrow aspiration/biopsy and whole blood sample collection for diagnostic analysis must be performed in case of any suspected signs of MDS/AML during the study treatment or post-treatment follow-up.

# 7 Efficacy & Safety Assessment and Exploratory Research

## 7.1 Efficacy Assessment

Contrast-enhanced CT or MRI scans of the abdomen, pelvis and other clinically indicated areas must be performed at baseline and during follow up. All baseline tumor imaging assessments must be performed within 28 days prior to the initiation of study medication. Results of routine imaging examinations before the informed consent can be used for study analysis if they are performed within the time range specified in the protocol and meet the image data collection requirements.

The imaging examinations of the abdomen, pelvis and other clinically indicated areas shall be performed once every 2 treatment cycles (8 weeks), until disease progression; if the disease does not progress by the end of the 14th cycle, the examination shall be performed once every 12 weeks, until disease progression. Prior to the analysis of the primary endpoint, all imaging data shall be assessed by blinded independent central review (BICR).

Tumor assessments will be performed strictly according to the plan. In the event that an imaging evaluation is performed at an unplanned time and no disease progression is found, the subsequent tumor evaluations shall still follow the original plan.

In this study, the tumor imaging evaluation will be performed based on RECIST v1.1 (mRECIST1.1) (Appendix 3) criteria, and the objective tumor responses will be classified as below:

The objective tumor responses based on the evaluation to target lesion include: complete response (CR), partial response (PR), stable disease (SD), progression of disease (PD) and not evaluable (NE).

The objective tumor responses based on the evaluation to non-target lesion include: complete response (CR), non-complete response/non-disease progression (NN), progression of disease (PD) and not evaluable (NE).

For tumor imaging evaluation of patients with no baseline lesion (neither measurable lesions nor immeasurable lesions), the objective tumor responses include: no disease lesion (ND), progression of disease (PD) and not evaluable (NE).

The determination of disease progression (PD) shall meet any of the following criteria by evaluating target lesions, and/or non-target lesions, and/or new lesions:

For target lesions: When lesions ‘fragment’, the individual lesion diameters should be added together to calculate the target lesion sum. The minimum of the sum during the study (including the sum at baseline, if it is the minimum) is taken as a reference. The sum must be increased by at least 20% relatively and 5mm absolutely.

For non-target lesions: there must be clear evidence of disease progression.

- - If the patient has a concomitant measurable disease: a “significant disease progression” conclusion based on non-target lesion is drawn only when there is a substantial deterioration of the non-target lesion, with a significantly increased overall tumor load, even if the target lesion is still at SD or PR. Generally, a small “increase” in one or more non-target lesions is not enough to be considered as a significant progression of disease. Therefore, a conclusion of overall progression is seldom made only based on the change in non-target lesion, when the target lesion is at SD or PR.
  - If the patient has only unmeasurable disease: when the increase in overall tumor burden assessed by the change in unmeasurable lesions is comparable to the increase in the volume of measurable lesions required to be evaluated as PD (e.g., an increase in tumor burden manifests as an increase of 73% in its “volume”, is equivalent to a 20% increase in the diameter of the measurable lesion), it is considered to have disease progression. Similar examples also include the increase of pleural or peritoneal effusion from “trace” to “large”, significant increase of omental cake (disease progression of omental nodules leads to a significant increase in the thickness of omentum), change of lymph-vessel disease from local to disseminated, or the worsening of disease sufficient to require a change in therapy. Once “a significant progression of disease” is observed, the patient is considered to have an overall progression of disease at the time of evaluation.

For new lesions: The occurrence of a new malignant lesion typically indicates a disease progression; however, the evidence of new lesions shall be clear and definite: it cannot be attributed to differences in scanning techniques or changes in imaging examination patterns, nor can it be a suspected non-neoplastic lesions (such as some "New" bone lesions may only be the recovery or deterioration of the original lesions). If the new lesion is difficult to identify (e.g., it is too small), it can be clarified by subsequent treatment and follow-up. If it is confirmed to be a new lesion by subsequent scans, the patient shall be evaluated as having disease progression, and the time of progression shall be recorded as the scan date when the lesion was first discovered.

If disease progression is confirmed, the subject shall stop study treatment, and be followed up in accordance with the trial protocol for survival status, etc.

### 7.1.1 Primary Study Endpoint: PFS

The primary endpoint is PFS, defined as the time from randomization to occurrence of disease progression or death by any cause (whichever occurs first); and the disease progression shall be assessed by blinded independent, central review (BICR) of imaging examination, according to RECIST v1.1 criteria (Appendix 3).

The investigators will also evaluate objective response to tumor treatment and disease progression by assessing the target lesions, non-target lesions, and new lesions according to the mRECIST 1.1 criteria (Appendix 3). The date of disease progression will be determined as the earliest day when a tumor progression is observed as assessed by RECIST v1.1. The diagnostic basis of disease progression shall be recorded in the eCRF.

The diagnosis of disease progression will not be made based on only CA-125 progression but no imaging progression in line with the RECIST1.1 criteria. Such patients shall continue to receive study treatment until disease progression meeting the RECIST 1.1 criteria.

If the diagnosis of disease progression is based on an additional diagnostic examination, rather than on the existing (baseline) CT/MRI examination, the date of additional diagnostic examination shall be taken as the time of disease progression. However, in the event that a new lesion is just the one that previously suspected but could not be confirmed, the date of disease progression shall be the date when the lesion was first discovered.

#### 7.1.1.1 Blinded, independent, central image review

1. Once a patient is determined to have a disease progression by the investigator and/or their treatment is discontinued, all their imaging material shall be submitted for BICR.
2. The RECIST imaging evaluation will be performed by two independent imaging experts, an adjudicator will join in if necessary.
3. Once the BICR is completed, the results about disease progression will be notified to the site.
4. In case that a patient is determined by the investigator to have a disease progression, which is not supported by BICR, the patient may continue to receive study treatment if it is considered to be safe and meets other treatment criteria. However, the patient should continue to undergo a pre-arranged imaging evaluation until disease progression is BICR confirmed, regardless whether the patient continues to receive study treatment.

#### 7.1.1.2 Determination of Disease Progression Date

In view of the fact that the primary endpoint of the study is PFS, and the disease progression is determined based on blinded, independent, central image review (BICR), so the date of disease progression determined based on the imaging evaluation will depend on BICR.

### 7.1.2 Secondary Study Endpoints

The secondary study endpoints are indicators of other clinical benefits, including CFI, TFST and OS.

#### 7.1.2.1 Chemotherapy-free Interval (CFI)

Chemotherapy-free interval (CFI) refers to the interval from the last dose of platinum-containing treatment to the start of the next anti-cancer treatment (excluding maintenance treatment).

#### 7.1.2.2 Time to First Subsequent Treatment (TFST)

Time to first subsequent treatment (TFST) is defined as the time from the date of randomization in the study to the date when the first subsequent anti-cancer treatment starts.

#### 7.1.2.3 Overall Survival (OS)

Overall survival (OS) refers to the time from the date of randomization to death by any cause.

## 7.2 Safety Evaluation

### 7.2.1 Laboratory Tests

All laboratory tests will be performed at the time points as specified in Schedule for Study Visits ([Table 1](#表1)). All the following lab tests (as shown in [**Table** 9](#_Hlk486578389)) will be done in the local laboratories.

During the screening period, once the patients have signed the ICF, their blood samples will be collected and sent to the central laboratory designated by the sponsor for gBRCA mutation testing. The test results will be used as one of the random stratification factors (for patients who have been previously tested for gBRCA mutation, the results may be used for randomization if they are recognized by the investigator and the Sponsor, but the patient shall still need to submit the blood samples for central tests). Women of childbearing age need to undergo a pregnancy test. If the serum biochemistry, complete blood count, coagulation function and other tests are performed within 7 days (screening period) before the start of the study treatment, and the patient's condition has not changed or there are no new complications, it will be not necessary to repeat the test in the second visit (Day 1 of Cycle 1). Lab tests (excluding g*BRCA* mutation test) will be performed at the local research centers or laboratories nearby.

All lab tests will be performed according to the study visit schedule, but an increased frequency may be adopted if the investigator deems it necessary. All examination results shall be filed in the original case report and recorded in the eCRF.

Table 9 – Laboratory Tests

| **Complete blood count (CBC)** | **Clinical biochemistry (serum) and coagulation function** | **Urinalysis** |
| --- | --- | --- |
| Red blood cell count | Blood urea nitrogen, creatinine | Specific gravity |
| Hemoglobin | Albumin, total protein | Leukocyte esterase |
| Hematocrit | Amylase, LDH, blood glucose | Occult blood |
| WBC count and differential count (neutrophils, basophils, eosinophils, lymphocytes, monocytes) | Alkaline phosphatase (ALP), GGT, ALT, AST, total bilirubin | Glucose |
| Platelet count | Sodium, potassium, calcium, magnesium, chlorine | Protein, ketone body |
| Mean platelet volume (MPV) (if available) | CA-125 | Urobilinogen and bilirubin |
|  | Activated partial thromboplastin time (APTT), International normalized ratio for prothrombin time (INR) | Nitrite |

In the event that a patient is suspected to have MDS/AML, bone marrow smear and biopsy test must be completed by a local hematologist, whole blood sample will also be collected for cytogenetic analysis (mutations of select myeloid-associated genes). The tests shall be performed in accordance with the clinical standard, subject to the approval from the medical monitor of the Sponsor. The bone marrow smear/biopsy reports issued by the hematological specialist must include the World Health Organization classification and MDS/AML test results. Results reported will be entered into eCRF, and the original reports shall be maintained in the site.

### 7.2.2 Electrocardiogram Evaluation

12-lead ECG examination will be performed in accordance with the study visit schedule shown in Table 1, or at any time when any cardiac-related adverse event has been developed during the trial. The patient shall have a rest in the supine position at least for 10 minutes before the specified time point, prior to ECG examination. The same body position shall be adopted in each examination, and all ECG examination results shall be recorded properly. Three ECG records shall be gained in each time point, with a time interval of 2 minutes. A single standardized electrocardiograph shall be used as possible throughout the study period.

Each ECG evaluation will include the following parameters, such as heart rate, QRS wave group, QT interval, QTc and RR interval, etc. ECG results will be evaluated by the investigator or a specialist physician, and they will be recorded in the eCRF. If a clinical abnormality has been found in baseline (screening period) examination, the abnormality should be recorded in the patient's history; if a clinically significant abnormality has been reported during treatment, it will be reported as an AE.

### 7.2.3 Physical Examination

Standard physical examination will be performed in accordance with the study visit schedule shown in [**Table 1**](#_Hlk486577971) (including the appearance, skin, eyes, ENT, head and neck, heart, chest and lungs, abdomen, limbs, lymph nodes, musculoskeletal, nerves, and other body systems, if applicable). Height will be measured only in the screening visit.

### 7.2.4 Vital Signs

Examination of vital signs will be performed in accordance with the study visit schedule shown in [**Table 1**](#_Hlk486577971) (including respiratory rate, heart rate, blood pressure and body temperature). The patient shall have a rest for 10 minutes before examination, keeping the sitting position during measurement of vital signs.

### 7.2.5 Eastern Collaborative Oncology Group (ECOG) Performance Status

ECOG performance status evaluation will be performed in accordance with the study visit schedule stated in [Appendix 2](#_Hlk486579334).

# 8 Safety Monitoring, Reporting and Medical Treatment

## 8.1 Adverse Event (AE) – Definition

Adverse event is any untoward medical occurrence that occurs in a patient or clinical investigation subject administered an investigational drug and which does not necessarily have a causal relationship with the treatment. An AE can therefore be any unfavourable and unintended sign (including an abnormal laboratory finding, for example), symptom, or disease temporally associated with the use of an investigational drug, whether or not considered related to the investigational drug. Adverse events include serious adverse events (SAEs) and non-serious adverse events.

## 8.2 Serious Adverse Event (SAE) – Definition

SAE is defined as any untoward medical occurrence:

- results in death
- is life-threatening (This means that the patient is at immediate risk of death of the subject at the time of the event)
- requires inpatient hospitalization or prolongation of existing hospitalization
- results in persistent or significant disability/incapacity; results in a congenital anomaly/birth defect
- is an important medical event that may jeopardize the health of the patient or may require medical intervention to prevent one of the above outcomes.

The following events shall **not be reported as a** SAE:

- Any results expected by the investigator of the disease progression, including death.
- Hospitalization or extended hospital stay occurs due to financial problems or reimbursement issue.

## 8.3 Collection, Recording, Follow-up Visit and Reporting of Adverse Events

### 8.3.1 Time Limit for Collection of Adverse Events

Adverse events will be collected and recorded throughout the study, from signing of the informed consent (ICF) up to completion of the study (30 days after last dose of study drug). AE and SAE occurring in the period from signing of the informed consent up to the first dose of study drug shall be recorded in the pre-treatment symptom and sign form of the eCRF.

### 8.3.2 Follow-up of Unresolved AE

The investigator will continue to follow up any adverse event that still has not been resolved at the last visit of the study, until no clinical sign can be found or patients initiate a new anticancer therapy (whichever occurs first). Follow-up may not be recorded into the eCRF, but the Sponsor will have the right to ask the investigator to provide further information, if required

### 8.3.3 Elements for Collection of AE

- Wording of AE report- Medical terms shall be used, and it shall give the priority to using the name of the diagnosed disease as an adverse event name. If the diagnosis is unclear, symptoms and signs can be used. When the disease could be diagnosed later, the record could be updated with the diagnosed disease name. It shall be ensured that each AE refers to a single event, diagnosis result, sign and/or symptom. If a patient shows both vomit and diarrhea symptoms, for example, “diarrhea” and “vomiting” shall be recorded as two AEs respectively.
- Start date/time of AE – Generally, the time when the symptom occurs, rather than the time when the disease is diagnosed, shall be taken as the start time of AE, so as to avoid missing any safety information or underestimating any potential safety risk.
- Severity of AE (CTCAE grade 1-5) should be distinguished from “Serious AE” – The severity of AE shall be classified into 5 levels (grade 1-5), in accordance with the common terminology criteria for adverse events (CTCAE); if the severity of an AE could be classified into mild, moderate and severe levels, rather than five grades, it shall convert the AE classified in such pattern into the AE with 5 grades, with reference to the criteria recommended in CTCAE. It is noteworthy to distinguish a serious adverse event (SAE) from a severe AE, as it is necessary to determine “whether an AE is a SAE or not” based on the definition specified in Section [8.2](#_8.2_Serious_Adverse), whereas the severity of an AE refers to the intensity or level of the AE, which could be evaluated based on CTCAE generally. A severe AE is not necessarily a SAE, such as a vomit lasting for several hours, which could be considered as severe, but not a SAE. In contrast, a stroke that has caused the limited disability may only be classified as the stroke of mild level, but it is a SAE indeed.
- The investigator's assessment of the causal relationship between the study drug and AE (relevant or irrelevant, Y or N) – see Section [8.3.4](#_8.3.4_Collection_of) for details.
- Measures taken to the study drug (e.g. whether an AE leads to patient’s suspension of medication or reduction of dose etc.)
- Concomitant medication - It shall be clearly stated that the concomitant medication is to deal with a particular adverse event or serious adverse event, or to meet the clinical routine treatment needs, and they must be clearly distinguished in the SAE report.
- Consequence and outcome of AE – The outcome of AE includes cured/recovered, recovering/improving, unrecovered/unimproved, recovered/improved with a sequel, death and unknown.

### 8.3.4 Collection of Causal Relationship between Study Drug and AE

The investigator will assess the causality relationship between the AE and the study drug, and give a “Yes” or “No” answer to the question “Is there a reasonable possibility of the study drug causing the AE?” The question will be answered by taking account of following concerns:

- Whether there is a time relationship between the AE and the medication
- Whether it is possible to infer the occurrence of symptoms and signs from the ingredients contained in the study drug
- Whether it can be explained with other reasons
- Whether the symptoms/signs are mitigated or relieved after dose reduction or discontinuation
- Whether the symptoms/signs occur again or become worse after the medication is resumed
- Whether there are similar reports in domestic and foreign literature

If the available information is limited, conservative measures can be taken, and the relationship can be assessed as likely to be relevant, meanwhile indicating the reason.

### 8.3.5 Examination- and Test-Based AE

Laboratory tests, vital signs, ECG and other safety measurement indicators as designated in the study will be summarized in the clinical study report. The deterioration of these indicators, compared to the baseline, will only be reported as an AE when the criteria defined for SAE have been met, or when it has caused study treatment interruption, dose adjustment and discontinuation, or when the investigator believes that the abnormality needs to be reported as an adverse event.

When the deterioration of laboratory tests, vital signs, ECG and other safety measurement indicators is correlated with a clinical symptom, the clinical symptom will be reported as an AE, and the deterioration of above indicators can be considered as the additional information for the AE. The investigator shall use a clinical term (such as anemia) as possible, rather than a laboratory term (such as hemoglobin reduction).

### 8.3.6 Progression of Disease

Any event caused by progression of the disease will not be reported as an AE or SAE.

### 8.3.7 Management of Death Case

The investigator will report all deaths that have occurred during the study, including the safety follow-up period (30 days after the last dose), as follows:

- In the event of a death clearly caused by disease progression, the investigator will inform the inspector of the Sponsor at the next inspection visit, and enter it into the corresponding module of the eCRF, but will not report the death as a SAE.
- If it is not clear whether the death is caused by disease progression, the investigator will report the AE as a SAE, and inform the inspector and send a report to the interested party within 24 hours (also seeing Section [8.3.9](#_8.3.9_Reporting_of)); In the SAE report, the investigator will assess whether the disease progression has contributed to the death of the patient, as appropriate, and indicates the primary cause of death and other relevant factors.
- In the event of an unexplained death, the investigator must report it as a SAE within the time limit specified, and shall endeavor to find the cause as possible. Autopsy may be helpful to assess the cause of death, and if the autopsy has been done, the autopsy report will be sent to the sponsor.
- For deaths occurring after the safety follow-up period, if the death is related to the study drug based on the investigator’s judgement, the cause of death should be reported as an SAE.

### 8.3.8 Adverse Event of Special Interest (AESI)

The following adverse events that occur after administration of the study drug require special attention:

- Myelodysplastic syndrome (MDS) or acute myeloid leukemia (AML)
- Secondary cancer (newly occurring malignant tumors other than MDS/AML
- Non-infectious pneumonia (pneumonitis)
- Embryo-fetal toxicity

MDS/AML and other secondary malignancies must be reported until the patient dies or is lost to follow-up; non-infectious pneumonia (pneumonitis) is reported within 90 days of the last dose of study drug; pregnancy is reported within 180 days of the last dose of study drug. The investigator should report the AESI to the sponsor within 24 hours after awareness (if the AESI event meets the SAE standard, then report it directly according to the SAE process).

### 8.3.9 Reporting of SAE

Any SAE that occurs during the trial shall be reported in accordance with the SAE reporting procedure of the relevant regulatory authority or the independent ethics committee, regardless of whether it is related to the treatment or not.

The investigator shall adopt following measures:

1. Take appropriate medical measures immediately, if necessary;
2. Record serious adverse events in the adverse event form of eCRF, SAE report form and source file.
3. Send the signed SAE report form to the following three parties immediately (within 24 hours after being aware of such an event), including (A) the Independent Ethics Committee, (B) the Drug Safety Department of the Sponsor (via fax or e-mail listed below) and (C) the food and drug administration at provincial level in the jurisdiction (through the web system or fax as specified by the authority).

Please send SAE to the public email box as follows:

Drug Safety Department, Zai Lab Pharmaceuticals Co., Ltd. (the Sponsor): saereporting@ zailaboratory.com

Fax: +86 21 61633171

1. The investigator shall follow up and record the process of the event until it disappears or returns to the baseline level, or reaches a clinically stable state.

### 8.3.10 Pregnancy

Patients who have been enrolled in this study will be required to take contraceptive measures. Once a patient has been found pregnant after taking the study drug, the patient will discontinue the study drug and withdraw from the study. The investigator will report to the sponsor by telephone or fax within 24 hours after becoming aware of pregnancy and will follow up the pregnancy event up till the end of pregnancy and inform the Sponsor of the pregnancy outcome. If the pregnancy outcome meets the SAE classification criteria (e.g. spontaneous abortion or therapeutic abortion [any congenital abnormality detected in the aborted fetus will be recorded in such a case], stillbirth, neonatal death or congenital abnormality), the investigator will inform the Sponsor or designee by submitting an complete SAE report within 24 hours after becoming aware of the outcome via email or fax.

### 8.3.11 Overdose

So far there is no report about excessive use of ZL-2306 (Niraparib). There is no antidote of special effect for ZL-2306 (Niraparib). In the event of overdose, it is necessary to seek help from a physician, who will provide the appropriate supporting treatment based on the signs and symptoms.

## 8.4 Management of Toxicity Related to the Study Drug

### 8.4.1 Management of Non - hematological toxicity

A patient could interrupt the treatment and/or reduce the dose of the study drug at any time during the trial, if the patient could not tolerate the toxicity effect of any level. If it is determined that a CTCAE grade 3/4 non-hematologic toxicity is correlated with the treatment, the treatment must be interrupted. If the toxicity is resolved to the baseline or CTCAE grade 1 or less within 28 days ([**Table 10**](#_Hlk486578780)), the patient may restart treatment with the study drug, and if there is no effective preventive measure, the dose of medication will be reduced. If the event recurs at the similar or worse grade, treatment should be interrupted again, and the dose must be reduced after the treatment is resumed. No more than 2 dose reductions will be permitted, i.e. the minimum dose is 100 mg OD. If the toxic response has not been completely relieved or resolved to CTCAE grade 1 within 28 days after dose interruption, or if the dose has already been reduced twice to a level of 100 mg OD, the patient must permanently discontinue the treatment with ZL-2306 (Niraparib) or the matched placebo.

Table 10 – Dose Modification for Non-hematologic Toxicity

| CTCAE ≥Grade 3 non-hematologic treatment-related adverse events, or other adverse events of clinical significance or intolerable toxicity, with effective preventive measures for these adverse events | Interrupt the study drug and provide symptomatic supportive treatment;  Within 28 days, if toxicity recovers to baseline or ≤CTCAE grade 1 or less, and preventive measures can be taken, the study drug should be restarted at the same initial dose |
| --- | --- |
| CTCAE ≥Grade 3 non-hematologic adverse events related to treatment, or other adverse events of clinical significance or intolerable toxicity, without effective preventive measures for these adverse events | Interrupt the study drug and provide symptomatic supportive treatment;  If toxicity recovers to baseline or ≤CTCAE grade 1 or less within 28 days, the study drug should be restarted with a reduced dose. The reduced dose level should follow the specifications in Table 7 of the study protocol. |
| If CTCAE Grade ≥3, and non-hematologic treatment-related adverse events persist at Grade >1 for more than 28 days | Discontinue treatment |

### 8.4.2 Management of Hematologic Toxicity

The management of hematological toxicity will follow the following principles:

- If the hematologic toxicity fails to recover to the specified level during 4 weeks (28 days) dose interruption period, study drug treatment must be permanently discontinued
- If hematologic toxicity requiring drug reduction recurs at the minimum dose level (100 mg QD), discontinuation of study treatment should be discussed with the sponsor.
- If the patient is diagnosed to have MDS or AML, study drug treatment must be terminated.

Detailed criteria for dose interruption and modification regarding different hematologic toxicities are shown in [Figure 2](#图2) - [4](#图4).

Figure 2. Management of thrombocytopenia


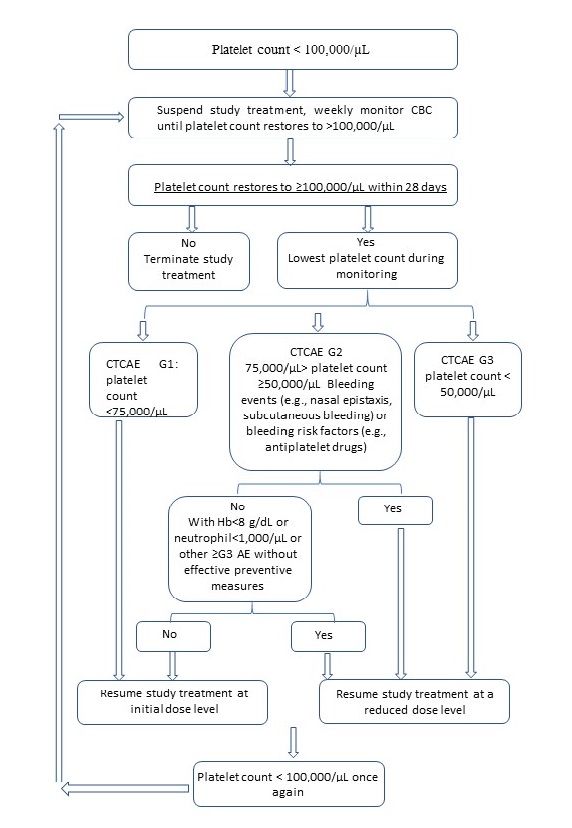


Figure 3 Management of neutropenia


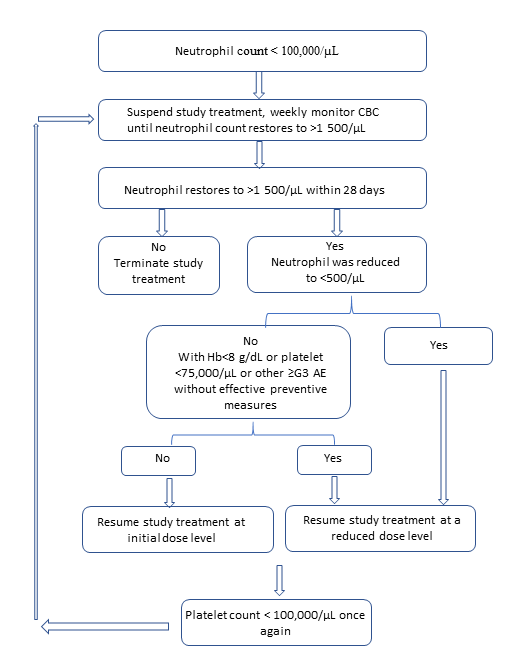


Figure 4. Management of anemia


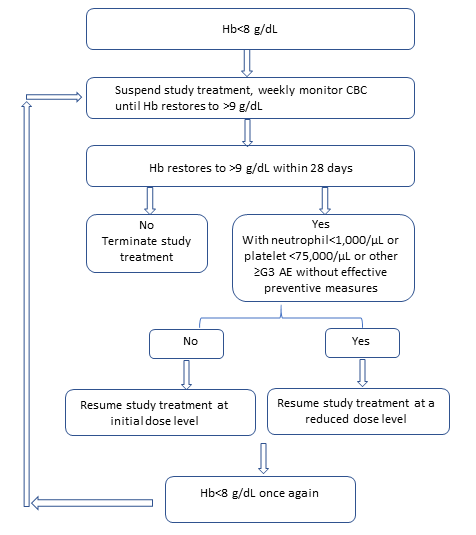


In the presence of thrombocytopenia, if platelet count recovers to the specified level after interruption, the same dose or a lower level can be used when the study treatment is restarted (Figure 2); if platelet count fails to recover to ≥100,000 /μL within 28 days after interruption, the treatment must be discontinued.

In the event of dose interruption due to hematologic toxicity, weekly CBC shall be performed until the AE is relieved; when the blood cell counts recover to the specified level, the medication could be restarted (at initial dose or a lower level). In order to ensure the dose safety after the medication is restarted, CBC monitoring shall be performed according to the frequency shown in [**Table 11**](#图11), taking the date when the medication is restarted as the first day. If the resumption is hematologically tolerated after consecutive 4 weeks, the test frequency may return to once a month. CBC can be performed at local hospital, but the results should reported to the investigator in a timely manner to ensure the safety. For patients who meet the criteria for dose increment after dose reduction, CBC test shall also be performed at least once a week after the new dose is started.

Table 11 – Schedule for Safety Monitoring after Medication is Restored from a Suspension due to Hematologic Toxicity

|  | Medication resumed | 8^th^ day  (±2days) | 15^th^ day  (±2days) | 18^th^ day  (±1day) | 22^nd^ day  (±2days) | 29^th^ day  (±2days) | Return to the frequency shown in Table 1 |
| --- | --- | --- | --- | --- | --- | --- | --- |
| CBC | X | X | X | X | X | X | X |

Platelet transfusion, red blood cell transfusion, hematopoietic cell growth factor or colony stimulating factor (GCSF) can be administered as supportive therapy acccording to clinical practice of each site.

Once a patient is diagnosed to have MDS or AML by a hematologic specialist, she must permanently discontinue from the study treatment.

If a patient receives a major surgery on treatment, it is allowed to interrupt the study treatment for up to 28 days.

After dose reduction from 200 mg/d to 100 mg/d, if the drug is well tolerated for at least one cycle, re-escalation to 200 mg/d can be considered. If the drug is well tolerated for at least 2 cycles after dose reduction from initial 300mg/d to 200mg/d, the dose may be re-escalated to 300 mg QD. Any dose increment of study drug after the dose reduction must be discussed with the sponsor’s inspector.

All dose interruptions or dose reductions (including missing doses) and the reasons are to recorded in the eCRF form.

## 8.5 Data Monitoring Committee (DMC)

Given that this study is a randomized, double-blind, placebo-controlled trial, a data monitoring committee (DMC) will be established shortly after the commencement of the trial, and review and assessment of safety data in a systematic manner will be provided to safeguard interest and safety of the participating patients. The DMC will consist of 3 independent individuals, including 1 biostatistician and 2 physicians. It is responsible for reviewing and assessing the safety information, and making recommendations to the sponsor whether the study should be continued or stopped, based on its expertise and experience.

The responsibilities of the DMC's membership and related operating procedures are defined in the DMC section.

# 9 Statistic Analysis

Detailed statistic information could be found in the Statistic Analysis Plan (SAP), which will be finalized before the database is locked. All changes to pre-defined analytical methods will be described in the final Clinical Study Report (CSR).

## 9.1 Analytical Data Set

Intent-to-treat population (ITT) –defined as all subjects randomized into the trial, and the ITT population will be taken as the major population for analysis. Analysis based on the ITT population will be performed per group to which the subjects were randomized.

Per-protocol population (PP) – defined as randomized subjects with no major protocol deviation that may significantly affect the efficacy evaluation. The specific definition of the PP population will be established before the database is locked. PP-based analysis will be served as the supportive analysis for ITT-based analysis, and it will be performed based on the actual treatment group.

Safety analysis population (Safety) – defined as all subjects who have received at least one dose of study drug. The safety analysis population is the primary population for safety analysis, and it will be analyzed based on the actual treatment group.

## 9.2 Demographics, Medical History, Baseline Characteristics and Combined Medication

The descriptive statistical method will be used to summarize the demographics, baseline characteristics, medical history and concomitant medication prior to administration of study drug and during the study.

## 9.3 Efficacy Analysis

The primary endpoint of this study is PFS, and the overall false positive rate will be controlled at the level of 0.05. The study analysis will be performed when approximately 155 progression events have occurred or 12 months after the randomization of the last patient (whichever occurs first). Detailed information on statistical analysis of the efficacy endpoints (including the sensitivity analysis to be conducted) can be found in the Statistic Analysis Plan (SAP).

### 9.3.1 Primary Efficacy Endpoint

The primary endpoint is PFS, defined as the time from randomization to occurrence of disease progression or death from any cause: the disease progression will be determined through the blinded independent central imaging assessment based on RECIST v1.1. PFS data will be censored in accordance with Table 12, for the primary analysis.

Table 12 – Censoring Rules for Primary Endpoint Analysis (PFS)

| **Situation** | **Censoring date** |
| --- | --- |
| No baseline radiologic assessment | Date of Randomization |
| No post-baseline radiologic assessment (and no death before the first scheduled radiologic assessment) | Date of Randomization |
| No disease progression or death during the study | Date of the last radiologic assessment |
| Documented disease progression or death after 2 or more consecutive missing radiologic assessments | Date of the last radiologic assessment |
| Having received new anti-tumor treatment before disease progression | Date of the last radiologic assessment before the new anti-tumor treatment |

The primary endpoint PFS analysis will be performed using log-rank test, and adjusted for randomization stratification factors. In addition, the Cox proportional hazard model that takes account of treatment group and stratification factors will be used to estimate the hazard ratio and its 95% confidence interval. PFS will also be descriptively summarized using Kaplan-Meier methodology.

The exploratory subgroup analyses will be performed for the primary endpoint in the study, based on the baseline characteristics, including age, new adjuvant chemotherapy, intraperitoneal chemotherapy, disease progression prior to enrollment, overall best response (CR and PR) to the last platinum-based treatment regimen, the number of previous chemotherapy lines (if the sample size is sufficient) and type of *BRCA* mutation etc.

See SAP for detailed information.

### 9.3.2 Secondary and Exploratory Efficacy Endpoint

The log-rank test, adjusted by the stratification factors, will be adopted for analysis of the secondary endpoints, such as PFS, TFST and OS. In addition, the hazard ratio and its 95% confidence interval will be estimated using a Cox proportional hazard model that takes account of the treatment and stratification factors. OS will be descriptively summarized using Kaplan-Meier method.

## 9.4 Safety Analyses

The treatment emergent adverse events (TEAE) and concomitant treatment will be summarized by treatment group. Clinical laboratory parameters, vital signs and ECG will be summarized by treatment group and study visits. The observed values from each visit in the test and their changes from the baseline will be described in the analysis. Additionally, a thresholds of marked abnormalities for specific safety parameters will be predefined. Incidence of marked abnormalities and shift tables will be presented.

## 9.5 Sample Size

The following statistical test hypothesis will be used in the trial:

H0: PFS (t) placebo = PFS (t) ZL-2306 (Niraparib)

Ha: PFS (t) placebo < PFS (t) ZL-2306 (Niraparib)

PFS (t) represents the progression free survivorship function at the time, t. PFS will be analyzed once in the test. The overall false positive rate will be controlled at 0.05 level.

Although it is anticipated that the treatment effect may be better in the gBRCA mutation group, the sample size calculation is based on an overall patient hazard ratio of 0.54 (the assumed median PFS for the niraparib and placebo group is 9.3 months and 5.0 months, respectively), an enrollment time of approximately 16 months, minimum follow-up time of approximately 7 months, and a dropout rate of 20%. Approximately 240 patients (160 in the ZL-2306 (Niraparib) group and 80 in the placebo group) will be randomized to obtain 155 PFS events, the study will provide a 95% power with a 2-sided significance level of 0.05.

The assumption for sample size calculation as above is mainly from a phase III, randomized, double-blind, multicenter global clinical study in which Niraparib vs placebo as maintenance treatment were evaluated in 553 patients with platinum-sensitive recurrent ovarian cancer. The hazard ratio of overall non-gBRCAmut patients was 0.45, with 95% confidence interval of 0.34 to 0.61, and the median PFS for Niraparib group and placebo arm was 21.0 months and 5.5 months respectively. In view of small proportion of Asian patients in previous clinical studies and the difference between the diagnosis and treatment of ovarian cancer in China and European and American countries, a more conservative hazard ratio assumption is adopted in this trial. The HR in the overall population is assumed to be 0.54.

# 10 Management of Clinical Trial

## 10.1 Declaration

This study will be conducted in accordance with the Declaration of Helsinki, Good Clinical Practice (GCP) and laws and regulations for clinical trials of drugs in China.

## 10.2 Ethics

It is the responsibility of the investigator to provide the Ethics Committee with the clinical trial protocol, informed consent form and information to the patient, in order to obtain an independent approval document for the implementation of the clinical study.

The approval of the Ethics Committee must be obtained before the start of the study, which will be issued to the investigator in written, and then the investigator must send a copy of such an approval to the Sponsor of the study. The approval document of the Ethics Committee shall be attached with a list of all the members of the Committee who have participated in the approval discussion and their respective responsibilities.

During the study, the investigator should inform the Ethics Committee of any questions related to the safety of clinical research in time, such as changes in clinical study protocol or patient informed consent and serious adverse events developed in the clinical study. The Ethics Committee should also be informed of the completion of the clinical study or early termination (if any).

## 10.3 Review of Original Records

The investigator must properly deal with all data obtained during the clinical study, to protect the rights and privacy of the patients involved in the clinical study. The investigator shall agree that the supervisor/auditor/inspector could review and check the clinical research data as required, in order to verify the accuracy of the original data and to understand the progress of the study. If the original records cannot be verified, the investigator should agree to assist the supervisor/auditor/inspector to further confirm the quality of the data.

## 10.4 Quality Assurance and Audit

All drugs and materials used in the clinical study must be subject to quality control. The Sponsor, its designee and the relevant medical management authority all have the right to conduct an audit to the clinical study, in order to ensure the integrity of the data recorded in the clinical study and the compliance with the requirements of the clinical study protocol. Patients involved in the clinical study will be informed of that an audit will be conducted by relevant person during the trial, but their privacy and data will be strictly protected.

## 10.5 Informed Consent Form (ICF)

It is the responsibility of the investigator to explain to each patient the purpose, method, benefits and potential risks of this clinical trial. The written informed consent form (ICF) must be obtained from the patient prior to commencement of any operation procedure related to the clinical trial. The ICF will be presented in written. The ICF must be dated and signed by the patient. In the event that the patient could not sign the ICF personally due to any reason, the ICF must be signed by the patient’s parents, legal guardian or protector. The investigator will maintain the original signed ICF in a good condition, and shall record the information on the signed ICF into the Case Report Form and relevant original trial recording document.

By signing the ICF, the patient shall also agree the Sponsor, regulatory authority, auditor and/or inspector to check source documents related to the clinical study, and the auditor must follow the confidentiality statement.

## 10.6 Protocol Amendment

After the study protocol is finalized, any amendments to the protocol must be documented in detail, and will be signed jointly by the investigator and the Sponsor, indicated with version number and date.

Protocol amendment requires the involvement of both the investigator and the Sponsor. The amended protocol will be submitted to the Ethics Committee for its approval, which could be implemented only after being approved.

## 10.7 Case Report Form (CRF)

The data of this study will be managed by the Data Department of CRO, as commissioned by the Sponsor, to ensure the integrity, completeness, privacy and traceability of the clinical trial data.

Information will be entered into the eCRF by the investigator or a person authorized by the investigator, and the evaluation on clinical/safety conclusion could only be signed by the investigator who has been granted with the medical license. Any changes made to the eCRF will be automatically recorded in the system after the investigator or the authorized person has completed the entry of original data.

## 10.8 Inspection

The Sponsor will designate an inspector for on-site inspection. The inspector shall operate according to the Standard Operation Procedure (SOP) of the company. The inspector will make a periodic visit from the start to end of the study.

The Inspector could access relevant raw data for this clinical study and review the CRF in accordance with the SOP, to confirm the completeness, accuracy and consistency with the original version of the information.

CRF, copies of laboratory data and medical test results must be readily available to the clinical inspector, auditor and health authorities. The inspector will be required to review all CRF and ICF.

## 10.9 Confidentiality Agreement and Privacy of Patients

The investigator must ensure to protect the confidentiality of information related to the Sponsor and the study drug, which is provided or disclosed for the purpose of cooperation in clinical research, and ensure to use such information only with authorization.

This commitment is independent, effective and persistent, until the permission of the sponsor is obtained.

The investigator will promise not to disclose to the third party any confidential information obtained from the company or product, or any confidential information provided or disclosed under the currently contractual relationship, and should use such information only to the agreed extent.

The investigator must ensure to protect the privacy of patients in the clinical trial. In all documents submitted to the Sponsor, only the patient’s number designated in the clinical trial, rather the name and admission number of the patient, could be used for referring to a patient. The investigator must properly keep the information on names and addresses of patients enrolled in the clinical trial, as well as the enrollment form that is corresponding to the designated numbers of patients enrolled in the clinical trial. These enrollment forms will be maintained by the investigator strictly according to the confidentiality principle.

# 11 Publication of Papers

Zai Lab (Shanghai) Co., Ltd. as the Sponsor, owns the exclusive right to this study. The author(s) and manuscript will reflect the collaboration among investigators, research institutions and the staff of Zai Lab (Shanghai) Co., Ltd. The author(s) shall be determined before the manuscript is prepared. Since a number of research institutions have participated in this study, individuals are not allowed to publish any article before the final report of the multicenter study is completed, unless otherwise agreed by Zai Lab (Shanghai) Co., Ltd. Additionally, Zai Lab (Shanghai) Co., Ltd. has the right of final decision with regard to the preparation and publication of the manuscript.

# 12 Archiving of Materials

The investigator will properly maintain the original records of clinical research, in accordance with relevant regulations. Copies of all clinical study documents must be retained for at least 5 years after the drug has been approved for marketing application. It is the responsibility of the Sponsor to inform the investigator/research institution about when the materials are no longer needed to be kept.

# 13 Reference

1. Wei KR, Li YM, Zheng RS et al. Ovary cancer in cadence and mortality in China. 2011. Chinese Journal of Cancer Research. 2015; 27(1): 38-43.
2. Fotopoulou C. Limitations to the use of carboplatin-based therapy in advanced ovarian cancer. European Journal of Cancer. Suppl. 2014; 12(2):13-16.
3. Gore ME, Fryatt I, Wiltshaw E, Dawson T. Treatment of relapsed carcinoma of the ovary with cisplatin or carboplatin following initial treatment with these compounds. Gynecologic Oncology. 1990; 36(2):207-211.
4. Markman M, Rothman R, Hakes T, et al. Second-line platinum therapy in patients with ovarian cancer previously treated with cisplatin. Journal of Clinical Oncology. 1991; 9(3):389-393.
5. NCI. BRCA1 and BRCA2: cancer risk and genetic testing. National Cancer Institute, 29 May 2009. https://www.cancer.gov/about-cancer/causes-prevention/genetics/brca-fact-sheet
6. Amé J-C, Spenlehauer C, de Murcia G. The PARP super-family. BioEssays. 2004; 26(8):882–93.
7. Kaelin WG, Jr. The concept of synthetic lethality in the context of anticancer therapy. Nature Review Cancer. 2005; 5(9):689-698.
8. Ledermann J, Harter P, Gourley C, et al. Olaparib maintenance therapy in patients with platinum-sensitive relapsed serous ovarian cancer: a preplanned retrospective analysis of outcomes by BRCA status in a randomised phase 2 trial. Lancet Oncology. 2014; 15(8):852-861.
9. Kaufman B, Shapira-Frommer R, Schmutzler RK, et al. Olaparib monotherapy in patients with advanced cancer and a germline BRCA1/2 mutation. Journal of Clinical Oncology. 2015; 33(3):244-250.
10. Ledermann J, Harter P, Gourley C, et al. Olaparib maintenance therapy in platinum-sensitive relapsed ovarian cancer. New England Journal of Medicine. 2012; 366(15):1382-1392.
11. Mirza MR, Monk BJ, Herrstedt J, Oza AM, Mahner S, Redondo A, et al. ZL-2306 (Niraparib) Maintenance Therapy in Platinum-Sensitive, Recurrent Ovarian Cancer. New England Journal of Medicine. 2016; October 7, online version.
12. Cancer Genome Atlas Research N. Integrated genomic analyses of ovarian carcinoma. Nature. 2011; 474 (73):609-615.
13. Farmer H, McCabe N, Lord CJ, et al. Targeting the DNA repair defect in BRCA mutant cells as a therapeutic strategy. Nature. 2005; 434(7035):917-921.
14. Matulonis UA, Harter P, Gourley C, et al. Olaparib maintenance therapy in patients with platinum-sensitive, relapsed serous ovarian cancer and a BRCA mutation: Overall survival adjusted for postprogression poly (adenosine diphosphate ribose) polymerase inhibitor therapy. Cancer. 2016; 122 (12):1844-1852.
15. FDA. Guidance for Industry: Clinical Trial Endpoints for the Approval of Cancer Drugs and Biologics. . In: CDER, ed2007.
16. Oza AM, Castonguay V, Tsoref D, et al. Progression-free survival in advanced ovarian cancer: a Canadian review and expert panel perspective. Current Oncology. 2011; 18 Suppl 2:S20-27.
17. GCIG. Definitions for response and progression in ovarian cancer clinical trials incorporating RECIST 1.1 and CA 125 agreed by the Gynecological Cancer Intergroup (GCIG). International Journal of Gynecologic Cancer. 2011; 21 (2): 419-23.

# 14 Appendices

## Appendix 1 Drugs Considered as a Sensitive Substrate of CYP1A2

| **Sensitive substrates of CYP1A2** |
| --- |
| Alosetron; Caffeine; Duloxetine; Melatonin; Ramelteon; Tacrine; Tizanidine |
| A sensitive substrate of CYP refers to a drug whose plasma AUC values have been shown to increase 5‐fold or higher when co-administered with a known CYP inhibitor or when the ratio of AUC in poor metabolizers vs extensive metabolizers is greater than 5-fold. |

## Appendix 2 ECOG Scale of Performance Status

| Description | Grade |
| --- | --- |
| Fully active, able to carry on all pre-disease performance without restriction | 0 |
| Restricted in physically strenuous activity, but ambulatory and able to carry out work of a light or sedentary nature, e.g., light house work, office work | 1 |
| Ambulatory and capable of all selfcare but unable to carry out any work activities; up and about more than 50% of waking hours | 2 |
| Capable of only limited selfcare; confined to bed or chair more than 50% of waking hours | 3 |
| Completely disabled; cannot carry on any selfcare; totally confined to bed or chair | 4 |

## Appendix 3 User Manual for Response Evaluation Criteria in Solid Tumors Version 1.1 (RECIST v1.1)

# 1. Overview of evaluation process

Tumor load will be recorded at baseline. First, the reviewer will determine which lesions are suitable for repeated quantitative evaluation (these are called "measurable" lesions). The reviewer will select a group out of the measurable lesions for quantitative follow-up throughout the study (these are called "target" lesions). RECIST v1.1 recommends using the method that maximizes the reproducibility or reliability of the measurement to measure the target lesion and calculate the tumor burden (sum of diameters) of the target lesion. All tumor lesions not selected for quantitative evaluation will be recorded and qualitatively followed up as "non-target" lesions.

The target lesions will be evaluated at each follow-up visit: the lesions will be appropriately measured to calculate the sum of their diameter; the sum at each visit will be compared the sum at baseline (to observe partial remission) and with the previous minimum (to observe disease progression). Reviewers will qualitatively evaluate non-target lesions and look for new lesions. The overall efficacy of a patient at a particular visit is assessed based on the information about target, non-target and new lesion. The relevant efficacy endpoints such as disease progression will be evaluated based on the sequential visit evaluation.

# 2. Measurement of tumor at baseline

## 2.1 Measurement of Lesions

All measurements should be recorded in metric notation, using calipers if clinically assessed. All baseline assessments should be performed as close as possible prior to the study start and within 4 weeks prior to treatment.

Measurements of lesions other than lymph nodes were performed using CT or MRI scans to measure the maximum diameter. Usually measured in the axial plane. Lymph nodes, even if not affected by tumors, are normal anatomical structures visible on imaging. At baseline and subsequent visits, only the short axis of lymph nodes is used to calculate the sum of the diameters of target lesions.

At baseline, tumor lesions/lymph nodes will be categorized measurable or non-measurable as follows:

Measurable Tumor lesions

Must be accurately measured in at least one dimension (longest diameter in the plane of measurement is to be recorded) with a minimum size of:

- CT: 10 mm by CT scan (CT scan slice thickness no greater than 5 mm) or twice the slice thickness (if slice thickness greater than 5 mm)
- MRI:10 mm or twice the scan layer thickness plus any gap thickness, whichever is greater
- 10 mm when assessed by caliper by clinical exam (lesions which cannot be accurately measured with calipers should be recorded as non-measurable).
- 20 mm by when assessed by chest X-ray

Malignant lymph nodes: To be considered pathologically enlarged and measurable, a lymph node must be ≥15 mm in short axis when assessed by CT scan (CT scan slice thickness recommended to be no greater than 5 mm). At baseline and in follow-up, only the short axis will be measured and followed. Nodal size is normally reported as two dimensions in the plane in which the image is obtained (for CT scan this is almost always the axial plane; for MRI the plane of acquisition may be axial, saggital or coronal). For example, an abdominal node which is reported as being 20 mm ×30 mm has a short axis of 20 mm and qualiﬁes as a malignant, measurable node. In this example, 20 mm should be recorded as the node measurement

Non-measurable Tumor lesions

Non-measurable Tumor lesions, including small lesions (longest diameter <10 mm or pathological lymph nodes with ≥10 to <15 mm short axis) as well as truly non-measurable lesions. Lesions considered truly non-measurable include: leptomeningeal disease, ascites, pleural or pericardial effusion, inﬂammatory breast disease, lymphangitic involvement of skin or lung, abdominal masses/abdominal organomegaly identiﬁed by physical exam that is not measurable by reproducible imaging techniques.

Pathological lymph nodes (with ≥10 to <15 mm short axis) should be considered non-measurable lesions. Lymph nodes that have a short axis <10 mm are considered non-pathological and should not be recorded or followed.

Special considerations regarding lesion measurability

Bone lesions, cystic lesions, and lesions previously treated with local therapy require particular comment:

**Bone lesions:**

- Bone scan, PET scan or plain ﬁlms are not considered adequate imaging techniques to measure bone lesions. However, these techniques can be used to conﬁrm the presence or disappearance of bone lesions.
- Lytic bone lesions or mixed lytic-blastic lesions, with identiﬁable soft tissue components, that can be evaluated by cross sectional imaging techniques such as CT or MRI and meet the deﬁnition of measurability above can be considered as measurable lesions
- Blastic bone lesions are non-measurable.

**Cystic lesions:**

- Simple cysts that meet the criteria for radiographically deﬁned should not be considered as malignant lesions (neither measurable nor non-measurable) since they are simple cysts by definition.
- “Cystic lesions” thought to represent cystic metastases can be considered as measurable lesions, if they meet the deﬁnition of measurability above. However, if non-cystic lesions are also present in the same patient, these are preferred as target lesions.

**Lesions with prior local treatment:**

- Tumor lesions situated in a previously irradiated area, or in an area subjected to other loco-regional therapy, are usually not considered as measurable unless it progressed. Study protocols should detail the conditions under which such lesions would be considered measurable.

Explanation of measurability of assessment methods

The same method of assessment and the same technique should be used for each identiﬁed and reported lesion at baseline and during follow-up. Imaging based evaluation should always be done rather than clinical examination unless the lesion(s) being followed cannot be imaged but are assessable by clinical exam.

**CT, MRI:** CT is the best currently available and reproducible method to measure lesions selected for response assessment. This guideline has deﬁned measurability of lesions on CT scan based on the assumption that CT slice thickness is 5 mm or less. When CT scans have slice thickness greater than 5 mm, the minimum size for a measurable lesion should be twice the slice thickness. MRI is also acceptable in certain situations (e.g. for body scans). MRI scan layer thickness refers to the distance between the top of one slice to the top of the next slice

If a patient experiences an allergic reaction to iodinated contrast agents during the study, the same method should be used (non-contrast CT for the chest, and MRI for the abdomen and pelvis). It should be noted that changes in the imaging modality may render lesions deemed as unevaluable beyond this time point.

**Clinical lesions:** Clinical lesions can only be considered as measurable lesions when they are superficial and have a diameter of ≥10 mm when assessed by caliper. For skin lesions, it is recommended to use a color photo of the lesion with a ruler or length standard as an archive.

**Chest X-ray:** Because CT is more sensitive than X-ray examination (especially to confirm new lesions), chest CT is preferred over chest X-ray. A chest X-ray lesion surrounded by inflatable lung tissue and clearly demarcated can be used as a measurable lesion. Nonetheless CT is always better than X-ray, even if contrast is not used.

**Ultrasound:** The method of ultrasound is not used in the assessment of the size of the lesion, and this detection method is not suitable for measuring the lesion.

# 3. Tumor response evaluation

## 3.1 Assessment of overall tumor burden and measurable disease

To assess objective response or disease progression, it is necessary to access the overall tumor burden at baseline and use this as a comparator for subsequent measurements

Baseline documentation of ‘target’ and ‘non-target’ lesions

Measurable disease is deﬁned by the presence of at least one measurable lesion. When more than one measurable lesions are present at baseline, all lesions (up to a maximum of ﬁve lesions in total, and a maximum of two lesions per organ) representative of all involved organs should be identiﬁed as target lesions and will be recorded and measured at baseline (this means in instances where patients have only one or two organ sites involved a maximum of two and four lesions respectively will be recorded). The other lesions of each organ are recorded as unmeasurable lesions (even on CT scan >10mm). Target lesions should be selected on the basis of their size (lesions with the longest diameter), be representative of all involved organs, but in addition should be those lend themselves to reproducible repeated measurements. It may be the case that, on occasion, the largest lesion does not lend itself to reproducible repeated measurement in which circumstance the nest largest lesion which can be measured reproducibly should be selected. Paired organs (lung, kidney, etc.) should be considered as a single organ. All lymph nodes are also regarded as a single organ.

A sum of the diameters (longest for non-nodal lesions, short axis for nodal lesions) for all target lesions will be calculated and reported as the baseline sum diameter. If lymph nodes are to be included in the sum, then as noted above, only the short axis is added into the sum. **The baseline sum diameters will be used as reference to further characterize any object tumor regression in the measurable dimension of the disease.**

All other lesions (or sites of disease) including pathological lymph nodes should be identified as non-target lesions and should also be recorded at baseline. Measurements are not required and these lesions should be followed as “resent”, “absent”, or in rare cases “unequivocal progression”. in addition, it is possible to record multiple non-target lesions involving the same organ as a single item on the case report form (e.g., “multiple enlarged pelvic lymph nodes” or “multiple liver metastases”).

## 3.2 Tumor Response criteria

### 3.2.1 Evaluation of Target Lesions

This section provides the definition used to determine objective tumor response for target lesions:

- Complete Response (CR): Complete disappearance of all target lesions. Any pathological lymph nodes (whether target or non-target) must have reduction to <10 mm in short axis.
- Partial Response (PR): At least a 30% decrease in the sum of diameters of target lesions, taking as reference the baseline sum diameters.
- Progressive Disease (PD): At least a 20% increase in the sum of diameters of target lesions, **taking as reference the smallest sum on study (this includes the baseline sum if that is the smallest on study).** In addition to the relative increase of 20%, the sum must also demonstrate an absolute increase of at least 5 mm.
- Stable Disease (SD): Neither sufﬁcient shrinkage to qualify for PR nor sufﬁcient increase to qualify for PD, taking as reference the smallest sum of diameters while on study

### 3.2.2 Special Considerations for Evaluation of Target Lesions

**Lymph nodes:** For lymph nodes identified as target lesions, the actual short-axis measurement value should always be recorded (measured in the same anatomical plane as the baseline examination), even if the lymph nodes shrink to <10 mm during the trial. This indicates that, because normal lymph nodes are defined as having a short axis <10 mm, if a lymph node is identified as a target lesion, even if it meets the criteria for complete response (CR), the sum of the lesions may not be 0. Therefore, when designing case report forms or using other data collection methods, data for lymph nodes as target lesions should be collected in a separate module/section, and if the CR criteria are met, all short-axis measurements of lymph nodes must be <10 mm. For PR, SD, and PD assessment criteria, the actual short-axis measurements of lymph nodes will be included in the sum of the diameters of target lesions.

**“Too small to measure” target lesions:** During the study, all target lesions (lymph nodes or non-lymph nodes) recorded at baseline should be documented in actual size (even if the lesions are very small, e.g., 2 mm) at each subsequent assessment. However, in some cases, lesions recorded as target lesions at baseline (including lymph node target lesions) may become unclear at subsequent CT scans, and radiologists may have difficulty determining an accurate measurement value, and may report it as “too small to measure”. In this case, a numerical value must still be recorded in the CRF, and the specific recording rules are as follows:

- If the radiologist believes the lesion may have disappeared, the measurement value should be recorded as 0 mm.
- If it is believed that the lesion still exists but looks unclear and too small to measure, it should be recorded as the default value of 5 mm and marked as BML (below measurable limit) (Note: Because the size of normal lymph nodes is usually determined, and retroperitoneal lymph nodes are usually enveloped in fat, this rule may not be suitable for lymph nodes; however, if it is believed that lymph nodes exist but look unclear and too small to measure, in this case, it will also be recorded as the default value of 5 mm and should be marked as BML). This default value is derived from the CT scan slice thickness (but this value does not change with different CT slice thicknesses). Since measurements of these lesions may be non-reproducible, providing this default value helps prevent tumor response or progression based on measurement errors. However, it needs to be emphasized that if the radiologist can provide an actual measurement value, even if it is <5 mm, it should be recorded, and not marked as BML (BML corresponds to <).

**Treatment-induced lesion splitting and fusion:** If non-lymph node lesions undergo splitting, the sum of the longest diameters of each split part should be calculated into the sum of the target lesions. Similarly, if lesion fusion (merging) occurs, and if there is an interface retained between the lesions, the sum of the longest diameters of each lesion should be calculated into the sum of the target lesions. If lesions completely fuse, the vector of the longest diameter should be taken as the maximum longest diameter of the “fusion lesion”.

### 3.2.3 Evaluation of non-target lesions

This section provides the criteria used to determine the tumor response for non-target lesions. While some non-target lesions may actually be measurable, they need not to be measured and instead should be assessed only qualitatively at the time points specified in the protocol.

- Complete Response (CR): Complete disappearance of all non-target lesions and normal tumor marker level. All lymph nodes must be non-pathological in size (<10 mm short axis).
- Non-CR/Non-PD: Persistence of any non-target lesion(s) and/or tumor marker level above the normal limits.
- Progressive Disease (PD): Unequivocal progression of pre-existing non-target lesions. The appearance of one or more new lesions indicate disease progression).

### 3.2.4 Special notes on assessment of progression of non-target lesions

The concept of progression of non-target lesions requires additional explanation as follows:

**When the patient also has measurable disease.** In this setting, to achieve ‘unequivocal progression’ on the basis of the non-target disease, there must be an overall level of substantial worsening in non-target disease such that, even in presence of SD or PR in target disease, the overall tumor burden has increased sufficiently to merit discontinuation of therapy (see examples in Appendix II and further details below). A modest ‘increase’ in the size of one or more non-target lesions is usually not sufficient to quality for unequivocal progression status. The designation of overall progression solely on the basis of change in non-target disease in the face of SD or PR of target disease will therefore be extremely rare.

**When the patient has only non-measurable disease.** This circumstance arises in some phase III trials when it is not a criterion of study entry to have measurable disease. The same general concepts apply here as noted above, however, in this instance there is no measurable disease assessment to factor into the interpretation of an increase in non-measurable disease burden. Because worsening in non-target disease cannot be easily quantified (by definition: if all lesions are truly non-measurable) a useful test that can be applied when assessing patients for unequivocal progression is to consider if the increase in overall disease burden based on the change in non-measurable disease is comparable in magnitude to the increase that would be required to declare PD for measurable disease: i.e. an increase in tumor burden representing an additional 73% increase in ‘volume’ (which is equivalent to a 20% increase diameter in a measurable lesion). Examples include an increase in a pleural effusion from ‘trace’ to ‘large’, an increase in lymphangitic disease from localized to widespread, or may be described in protocols as ‘sufficient to require a change in therapy’. If ‘unequivocal progression’ is seen, the patient should be considered to have had overall PD at that point. While it would be ideal to have objective criteria to apply to non-measurable disease, the very nature of that disease makes it impossible to do so, therefore the increase must be **substantial**.

### 3.2.5 Tumor imaging evaluation for patients without any lesion (either measurable or non-measurable) at baseline

Objective tumor response can be categorized as ND, PD and NE.

### 3.2.6 New lesions

The appearance of any new malignant lesions indicate disease progression; therefore, some comments on detection of new lesions are important. There are no specific criteria for the identification of new radiographic lesions; however, the finding of a new lesion should be unequivocal: i.e. not attributable to differences in scanning technique, change in imaging modality or findings thought to represent something other than tumor (for example, some ‘new’ bone lesions may be simply healing or ﬂare of pre-existing lesions).

This is particularly important when the patient’s baseline lesions show partial or complete response. For example, necrosis of a liver lesion may be reported on a CT scan report as a ‘new’ cystic lesion, which it is not.

A lesion identified on a follow-up study visit in an anatomical location that was not identified at baseline is considered a new lesion and will indicate disease progression. An example of this is the patient who has visceral disease at baseline and has a brain CT or MRI on study which reveals metastases. The patient’s brain metastases are considered to be evidence of PD even if he/she did not have brain imaging at baseline.

If a new lesion is equivocal, for example because of its small size, continued therapy and continued assessment will clarify if it represents truly new disease. If subsequent scans conﬁrm there is definitely a new lesion, then progression should be recorded using the date of the initial assessment.

**(18) Fluorodeoxyglucose Positron Emission Tomography (FDG-PET)**

While FDG-PET response assessments need additional study, it is sometimes reasonable to incorporate the use of FDG-PET scanning to complement CT scanning in assessment of progression (particularly possible ‘new’ disease). New lesions on the basis of FDG-PET imaging can be identified according to the following algorithm:

- Negative FDG-PET at baseline, with a positive FDG-PET at follow-up is a sign of PD based on a new lesion.
- No FDG-PET at baseline and a positive FDG-PET at follow-up:
- If the positive FDG-PET at follow-up corresponds to a new site of disease confirmed by CT, this is PD.
- If the positive FDG-PET at follow-up is not confirmed as a new site of disease on CT, (existing lesions or presence of lesions at sites of indeterminate PET positivity) additional follow-up CT scans are needed to determine if there is truly progression occurring at that site (if so, the date of PD will be the date of the initial abnormal FDG-PET scan).
- If the positive FDG-PET at follow-up corresponds to a pre-existing site of disease on CT that is not progressing on the basis of the anatomic images, this is not PD.

## 3.3 Considerations during response assessment

When lymph node lesions are included in the sum of target lesions and lymph nodes are reduced to a physiological size (< 10mm), measurement data may still appear in the scan results. Even if the lymph nodes appear normal, this measurement should be recorded to avoid exaggeration of disease progression based on lymph node enlargement. This means that the sum of tumor diameters in a CR patient may not be "0" in the case report form (eCRF) records.

If the patient's overall health deteriorates and the treatment must be discontinued, but there is no objective evidence of PD at that time point, the patient should be reported as "worsening of symptoms". Objective progression should be recorded even after treatment is terminated. Worsening of symptoms is only a cause of termination of study treatment, but not a technical term for assessing objective response. Objective response in such cases should be determined by a comprehensive assessment of target, non-target, and new lesions as listed in Tables 1 and 2.

In some cases, it is difficult to distinguish between residual foci and normal tissue. When the assessment of complete response depends on the result, it is recommended that residual disease should be assessed (using fine needle aspiration/biopsy) before being classified as CR.

For those who are uncertain of disease progression (e.g., very small and inexact new lesions, cystic changes or necrosis in existing lesions, etc.), treatment can be continued until the next scheduled evaluation. If disease progression is identified at the next planned assessment, the date of progression should be the date when disease progression is previously suspected.

## 3.4 Missing assessments and inevaluable confirmation

When no imaging/measurement is done at all at a particular time point, the patient is not evaluable (NE) at that time point. If only a subset of lesion measurements are made at an assessment, usually the case is also considered NE at that time point, unless a convincing argument can be made that the contribution of the individual missing lesion(s) would not change the assigned time point response. This would be most likely to happen in the case of PD.

For example, if a patient had a baseline sum of 50 mm with three measured lesions and at follow-up only two lesions were assessed, but those gave a sum of 80 mm, the patient will have achieved PD status, regardless of the contribution of the missing lesion. If one or more target lesions are not evaluated due to a lack of scanning or cannot be evaluated due to poor image quality or hindrance to observation, as the patient is considered unevaluable, the response status of the target lesions is deemed “Not Evaluable”. Similarly, if one or more non-target lesions are “Not Evaluated”, the response status of non-target lesions should be “Not Evaluable” (except when there is clear progression). If the response status of target lesions or non-target lesions is “Not Evaluable”, the overall response status will be “Not Evaluable” (except when there is clear evidence of progressive disease), as this is equivalent to being unevaluable at that time point.

## 3.5 Assessment of tumor remission status at specific time points

Assessment of tumor remission status is required at each time point specified in the protocol. Table 1 provides a summary of the tumor's overall remission status at each time point for patients with measurable disease at baseline.

Table 2 is used if the patient has only non-measurable (non-targeted) lesions.

**Table 1 Time point response: patients with target (+/- non-target) disease**

| **Target lesions** | **Non-target lesions** | **New lesions** | **Overall response** |
| --- | --- | --- | --- |
| CR | CR | No | CR |
| CR | Non-CR/non-PD | No | PR |
| CR | Not evaluated | No | PR |
| PR | Non-PD or not all evaluated | No | PR |
| SD | Non-PD or not all evaluated | No | SD |
| Not all evaluated | Non-PD | No | NE |
| PD | Any | Yes or No | PD |
| Any | PD | Yes or No | PD |
| Any | Any | Yes | PD |

CR = complete response, PR = partial response, SD = stable disease, PD = progressive disease, and NE = not evaluable.

**Table 2 Time point response: patients with non-target disease only**

| **Non-target lesions** | **New lesions** | **Overall response** |
| --- | --- | --- |
| CR | No | CR |
| Non-CR/non-PD | No | Non-CR/non-PD^a^ |
| Not all evaluated | No | NE |
| Unequivocal PD | Yes or No | PD |
| Any | Yes | PD |
| CR = complete response, PD = progressive disease, and NE = not evaluable.  a “Non-CR/non-PD” is preferred over “stable disease” for non-target disease since SD is increasingly used as endpoint for assessment of efficacy in some trials so to assign this category when no lesions can be measured is not advised. | | |

**Reference:**

Eisenhauer EA, Therasse P, Bogaerts J, et al. New response evaluation criteria in solid tumors: Revised RECIST guideline (version 1.1). Eur J Cancer. 2009 Jan; 45(2):228-47.
